# Understanding the factors governing the ammonia oxidation reaction by a mononuclear ruthenium complex†

**DOI:** 10.1039/d4sc02360a

**Published:** 2025-03-19

**Authors:** Guo Chen, Xiao-Lv Ding, Piao He, Tao Cheng, Yang Chen, Jian Lin, Xi Zhang, Shan Zhao, Na Qiao, Xiao-Yi Yi

**Affiliations:** a College of Chemistry and Chemical Engineering, Central South University Changsha Hunan 410083 P. R. China xyyi@csu.edu.cn; b School of Chemistry and Chemical Engineering, Southwest University Chongqing 400715 P. R. China; c CAS Key Laboratory of Science and Technology on Applied Catalysis, Dalian Institute of Chemical Physics, Chinese Academy of Sciences Dalian 116023 P. R. China

## Abstract

Precise regulation of the active site of molecular catalysts is appealing because it could provide insights into the catalytic mechanism and possibly provide a new strategy for catalyst design. A ruthenium complex, [Ru(dpp_Me, COMe_)(bipy)(Cl)] (CSU-3), containing –Me and –COMe substituted dipyridylpyrrole as a pincer ligand, was designed and synthesized. The CSU-3 complex featured a Cl^−^ ligand at the axial position as the active site for ammonia oxidation (AO), and is structurally analogous to AO catalyst [Ru(trpy)(dmabpy)(NH_3_)][PF_6_]_2_ (1) bearing a terpyridine ligand, but different from AO catalyst [Ru(dpp)(bipy)(NH_3_)] (CSU-2) containing unsubstituted dipyridylpyrrole as a hemilabile ligand with the active site at an equatorial position. To gain insight into the role of active-site and ligand regulation in the AO reaction, the structure and electrochemical properties of CSU-3 and its catalytic performance and mechanism for the AO reaction were comparably studied. Complex CSU-3 has good selective catalytic performance for the oxidation of ammonia to hydrazine with a turnover frequency (TOF) of 258.8 h^−1^ and N_2_H_4_ formation selectivity of 84.7% at *E*_app_ of 1.0 V. The DFT calculations reveal that N_2_H_4_ as a dominant product is generated *via* an ammonia nucleophilic attack of ruthenium(iv)-imide to form N_2_H_4_ followed by N_2_H_4_-by-NH_3_ substitution.

## Abstract 2

Precise regulation of the active site of molecular catalysts is appealing because it could provide insights into the catalytic mechanism and possibly provide a new strategy for catalyst design.

## Graphical abstract

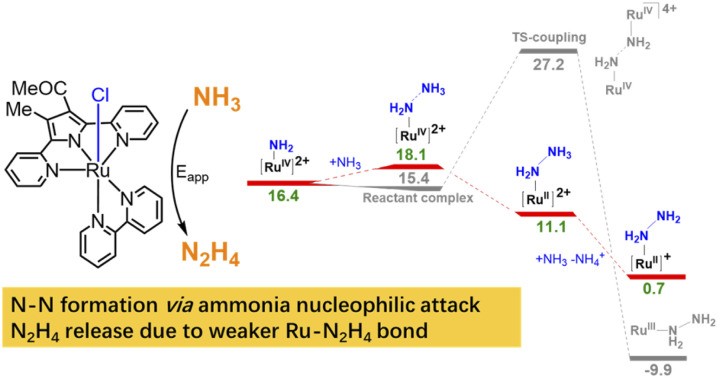

## Introduction

Ammonia (NH_3_) as a carbon-free alternative fuel is considered as one of the most important hydrogen carriers;^1–3^ however, the classical heterogeneous catalytic cracking reaction for NH_3_-to-H_2_ conversion requires a precious metal catalyst and high temperature, and is therefore relatively high cost. Molecular catalytic ammonia splitting is one of the appealing alternative methodologies to produce H_2_.^4,5^ Ammonia oxidation (AO) is a critical half-reaction in NH_3_-to-H_2_ conversion, and due to the involvement of multiple electron and proton transfers it is kinetically demanding. Molecular catalysts based on transition metals for the oxidation of ammonia might offer many attractive attributes, and a myriad of spectroscopic, kinetic, thermodynamic, and electrochemical techniques have been used to gain detailed insights into the bond-breaking and bond-forming processes.^6,7^

**Scheme 1:**
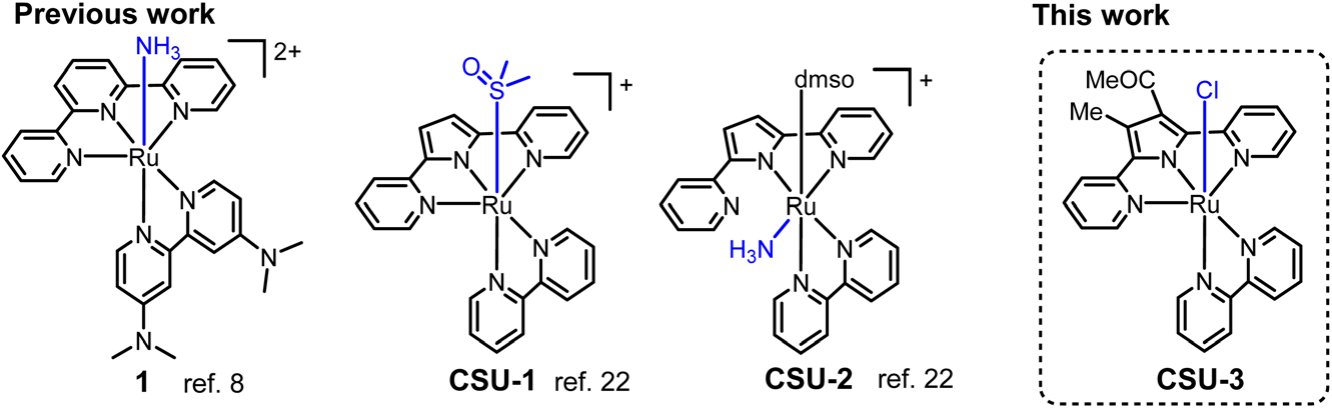
Molecular AO catalysts relevant to this work.

Numerous transition-metal complexes based on Ru,^8–15^ Fe,^16–18^ Cu,^19^ Ni,^20^ and Mn,^21^ with various ligands for catalytic AO have been developed,^22–24^ since the pioneering work using a [Ru(trpy)(dmabpy)(NH_3_)][PF_6_]_2_ (1, Scheme 1, trpy = 2,2′:6′,2′′-terpyridine, dmabpy = 4,4,-bis(dimethylamino)-2,2′-bipyridine) catalyst^8^ for AO was reported by Smith III, Hamann and co-workers. The electron rich –NMe_2_ group in 1 makes the catalyst decrease the onset potential of AO and triggers electrocatalytic oxidation of NH_3_ to generate N_2_*via* an ammonia nucleophilic attack (ANA) mechanism, with a TOF_N_2__ of 0.7 h^−1^. The N_2_H_4_-ligated intermediates were determined through NMR spectra. As shown in Scheme 1, we recently reported a distinct example of a Ru-based catalyst,^25*a*^ [Ru(K^3^-*N*,*N*′,*N*′′-dpp)(bipy)(dmso)][PF_6_] (CSU-1, bipy = 2,2′-bipyridine, Hdpp = 2,5-di(pyridin-2-yl)-1*H*-pyrrole) and [Ru(K^2^-*N*,*N*′-dpp)(bipy)(dmso)(NH_3_)][PF_6_] (CSU-2), which exhibits excellent electrocatalysis activity for the AO reaction to generate N_2_H_4_ with high selectivity (>99%) and high efficiency (TOF_N_2_H_4__ > 100 h^−1^). The mechanism studies show that it benefits from the lower barrier in N_2_H_4_ formation involving a bimolecular coupling of Ru^II^-aminyl or Ru^III^-iminyl intermediates, but is unfavorable for N_2_ formation *via* the ANA mechanism like in complex 1.

The anionic dipyridylpyrrole dpp^−^ ligand in CSU-1 and CSU-2 is structurally analogous with the neutral trpy ligand in 1; however, their ligated ruthenium complexes exhibit significant differences in their spatial configurations, selectivity, catalytic efficiency and even mechanism for AO. The factors governing ammonia oxidation seem very complicated and deserve further in-depth research. To gain an insight into the role of the active-site in catalysis for the oxidation of ammonia, herein we design a mononuclear ruthenium(ii) complex [Ru(dpp_Me, COMe_)(bipy)(Cl)] (CSU-3, Hdpp_Me, COMe_ = 1-(4-methyl-2,5-di(pyridin-2-yl)-1*H*-pyrrol-3-yl)ethan-1-one, Scheme 1) and comparably study its electrocatalysis of the AO reaction in CH_3_CN media.

To avoid the hemilability of the dpp^−^ ligand to form bidentate K^2^-*N*,*N*′-coordination modes like in CSU-2, we chose the dpp_Me, COMe_^−^ ligand in CSU-3. The steric effect of the substituted –Me and –COMe groups causes a smaller interior bond angle (av. 116.3°) between the pyrrole and pyridine in the free dpp_Me, COMe_^−^ ligand (Fig. S1–S4,† crystallographic data in Tables S1 and S2†) compared to the 121.4° in the free dpp^−^ ligand.^23^ This leads to an increase in the binding strength of the Ru–N bond between the Ru and N of dpp_Me, COMe_^−^ in CSU-3, and causes dpp_Me, COMe_^−^ binding to the Ru center with a N^N^N coordination mode, like trpy in 1. Thus, the active site in CSU-3 was regulated to the axial position for a comparative study.

## Results and discussion

### Synthesis and characterization

**Fig. 1:**
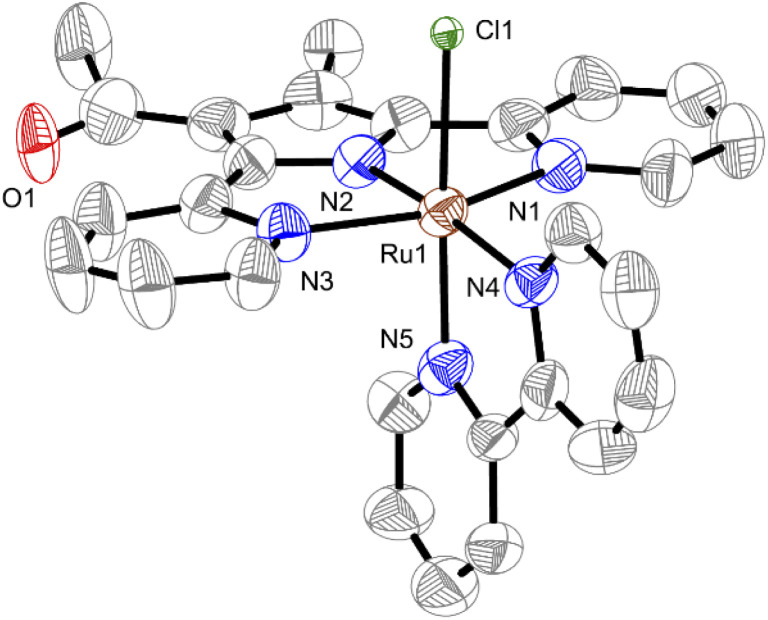
Solid-state structure of CSU-3. The hydrogen atoms are omitted for clarity. Bond distances (Å): Ru1–N1, 2.107(5); Ru1–N2, 1.907(4); Ru1–N3, 2.101(4); Ru1–N4, 2.033(5); Ru1–N5, 2.065(4); Ru1–Cl, 2.4297(14).

Treatment of [Ru(dmso)_4_(Cl)_2_], Hdpp_Me, COMe_ and bipy in the presence of Et_3_N under refluxing toluene gives a red solid, which is redissolved in MeOH and refluxed for 5 d to afford CSU-3 in 23% yield. Complex CSU-3 was fully characterized using NMR, elemental analysis, and infrared spectroscopy (Fig. S5–S8†). The ESI-MS results display a parent peak at *m*/*z* 569.0282 assigned to [M]^+^. Compared to the ^1^H NMR of CSU-1, the resonance signal of ligated dmso is absent in CSU-3. This result is consistent with what was observed in single crystal X-ray diffraction analysis. As shown in Fig. 1, CSU-3 displays a slightly distorted octahedral geometry around the ruthenium center. The Cl^−^ ligand in CSU-3 coordinates with the ruthenium center at the axial position, unlike in CSU-1 with a dmso ligand at the axial position. Due to the steric effect of the substituted –Me and –COMe groups on the pyrrole unit, as mentioned in the introduction section, the dpp_Me, COMe_^−^ ligand in CSU-3 as an N^N^N pincer ligand strongly coordinates to the ruthenium center at the equatorial position. This is mirrored by the shorter bond distance of the ruthenium center and the terminal pyridine of the dpp_Me,COMe_^−^ ligand in CSU-3 with Ru1–N1 of 2.107(5) Å) and Ru1–N3 of 2.101(4) Å compared to that in CSU-1 (2.136(2) Å and 2.137(2) Å). Unlike in CSU-1, in complex CSU-3, the outer donor group is not readily decoordinated to supply a vacant site for coordination of the incoming NH_3_. The NMR and UV-vis monitoring experiments also confirm that CSU-3 is very stable in the presence of a coordinating solvent and even NH_3_ (Fig. S9†) due to the negative charge of the Cl^−^ ligand, which makes it a poor leaving group. Thus, the corresponding NH_3_-ligated complex could not be obtained by direct Cl^−^-by-NH_3_ substitution of CSU-3.

Complex CSU-3 is treated with AgOTf (OTf^−^ = trifluoromethylsulfonate) in CH_3_CN to remove the Cl^−^ ligand, and then ammonia gas is bubbled into the filtrate solution to give NH_3_-ligated complex [Ru(dpp_Me, COMe_)(bipy)(NH_3_)]OTf ([CSU-3-NH_3_]OTf) (Fig. S10–S13†). Its ^1^H NMR spectrum shows a newly added single broad peak at 2.47 ppm due to the incoming NH_3_ (Fig. S10†), which is consistent with what was observed in its ESI-MS spectrum with a parent peak at *m*/*z* 551.1135 for [CSU-3-NH_3_]^+^ (Fig. S12†) and elemental analysis.

### Electrochemical and electrocatalytic performances

The electrochemical behavior of CSU-3 was studied using 0.1 M Bu_4_NPF_6_ in CH_3_CN as the electrolyte, glassy carbon as the working electrode, Pt wire as the counter electrode and Ag/AgCl in saturated KCl aqueous solution as the reference electrode. Unless otherwise specified, all potentials are converted into *E*_1/2_*versus* Cp_2_Fe^+/0^ in CH_3_CN by adding −0.43 V to the measured potential.

**Fig. 2:**
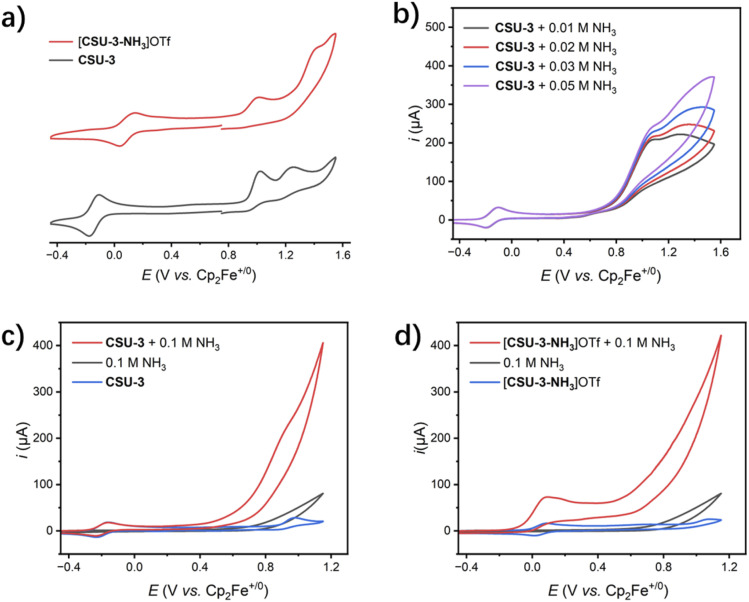
(a) CV of CSU-3 and [CSU-3-NH_3_]OTf in MeCN solution; (b) CV of CSU-3 in MeCN in the presence of NH_3_ with various concentrations (0.01–0.05 mM); (c) CV of CSU-3 in MeCN in the presence of 0.10 M NH_3_; (d) CV of [CSU-3-NH_3_]OTf in MeCN in the presence of 0.10 M NH_3_. Conditions: [Ru]: = 1 mM, rate 0.1 V s^−1^, 0.1 M Bu_4_NPF_6_ as the supporting electrolyte, platinum wire as the working electrode, potential referenced to the Cp_2_Fe^+/0^ redox couple.

As shown in Fig. 2a, the cyclic voltammogram (CV) of CSU-3 displays a reversible wave at −0.14 V followed by two irreversible waves at 0.98 and 1.21 V, which are assigned to Ru^III/II^, Ru^IV/III^ and the ligand oxidation, respectively. The redox potential of Ru^III/II^ is significantly lower than that of 1 (Ru^III/II^ 0.055 V *vs.* Cp_2_Fe^+/0^ in THF), CSU-1 (0.47 V *vs.* Cp_2_Fe^+/0^ in CH_3_CN) and CSU-2 (0.43 V *vs.* Cp_2_Fe^+/0^ in CH_3_CN). Obviously, the redox behavior of the metal center in CSU-3 is sensitive to the electron donor nature of the dpp_Me, COMe_^−^ ligand with a methyl substituent. Compared to CSU-1 and CSU-2 with a π-accepting dmso ligand at the axial position, the π-electron donating Cl^−^ ligand at the axial position is also a possible reason for the significant negative-shift of redox potential in CSU-3.

For complex [CSU-3-NH_3_]OTf, the first reversible wave (0.09 V) and the second irreversible wave (0.97 V) were attributed to continued oxidation of the ruthenium center (Ru^II^ ⟶ Ru^III^ ⟶ Ru^IV^). The third irreversible wave at 1.34 V is due to ligand oxidation. Compared to CSU-3, the redox potential of the first reversible Ru^III/II^ wave in [CSU-3-NH_3_]OTf is positively shifted. This suggests that the electron donating ability of NH_3_ is weaker than that of the negatively charged Cl^−^ ligand. The latter acts as a π donor increasing the electron density of the ruthenium center.

The CV plot of CSU-3 in the presence of NH_3_ with various concentrations (0.01–0.05 M), as shown in Fig. 2b, illustrates that the Ru^III/II^ couple remains unchanged, and a new oxidation wave (∼1.06 V) appears for the Ru^IV^ species. Subsequently, a strong catalytic current (*i*_cat_) is observed (Fig. 2c), suggesting that the Ru^IV^ species triggers the oxidation of ammonia. Obviously, when the ruthenium center of CSU-3 is oxidized to the Ru^IV^ oxidation state, an EC process occurs to generate Ru^IV^–NH_3_*via* Cl-by-NH_3_ substitution of the Ru^IV^ species from 2e^−^ oxidation of CSU-3, which is also supported by the DFT calculations. According to the CV plot of [CSU-3-NH_3_]OTf (Fig. 2d), in addition to the Ru^IV^ species, the Ru^III^ species from [CSU-3-NH_3_]OTf also triggers the oxidation of ammonia, which is very similar to what is observed in the CSU-1, CSU-2 and [Ru(K^3^-*N*,*N*′,*N*′′-dpp)(bpy)(L)]·PF_6_ (L = pyridine; 4-methylpyridine; pyrimidine; isoquinoline) catalyst systems and Ru(K^3^-*N*′,*N*′′-dpp)(trpy)(NH_3_)]·PF_6_.^25*b*,*c*^

As shown in Fig. S14 and S15,† complexes CSU-3 and [CSU-3-NH_3_]OTf exhibit satisfactory stability, corroborated by 100 consecutive cyclic voltammetry cycles, in which no new redox wave appears and the attenuation of the catalytic current is not significant. A post-catalysis and thoroughly rinsed carbon cloth working electrode displayed no catalytic activity (Fig. S16†). This indicates that the catalytic process of CSU-3 and [CSU-3-NH_3_]OTf is homogenous.

Controlled potential coulometry (CPC) experiments were conducted in a sealed Schlenk electrolytic cell with a 0.01 mM ruthenium catalyst solution containing 0.2 M (or 2.0 M) NH_3_ and 0.1 M [Bu_4_N][PF_6_] supporting electrolyte in anhydrous MeCN. The detection method for the possible products (H_2_, N_2_, N_2_H_4_, NO_2_^−^ and NO_3_^−^) and blank experiments is described in the ESI (Fig. S17–S21 and Table S5).† The data of catalytic performance are listed in Table 1 and Fig. S22.†

**Table 1:** The electrocatalytic performances of CSU-3 and [CSU-3-NH_3_]OTf^a^

Entry	Cat.	*c*_NH_3__ (mol L^−1^)	*E*_app_ (V)	TOF_H_2__ (h^−1^) *n*_H_2__ (μmol)	TOF_N_2_H_4__ (h^−1^) *n*_N_2_H_4__ (μmol)	TOF_N_2__ (h^−1^) *n*_N_2__(μmol)	*Q* b (C)	FE_N_2_H_4__^c^(%)	*S*_N_2_H_4__ d(%)
1	CSU-3	0.2	0.2	Trace	Trace	Trace		—	—
2	CSU-3	2.0	0.2	Trace	Trace	Trace		—	—
3	CSU-3	0.2	1.0	141.4	140.3	1.0	25.3	85.6	99.3
				**113.1**	**112.2**	**0.8**			
4	CSU-3	2.0	1.0	276.0	258.5	2.9	47.1	84.7	98.9
				**220.8**	**206.8**	**2.3**			
5	[CSU-3-NH_3_]OTf	0.2	0.2	5.0	4.8	Trace	0.8	87.9	100
				**4.0**	**3.8**				
6	[CSU-3-NH_3_]OTf	2.0	0.2	19.5	19.1	Trace	3.3	89.5	100
				**15.6**	**15.3**				
7	[CSU-3-NH_3_]OTf	0.2	1.0	169.3	165.3	1.8	28.5	89.5	98.9
				**135.4**	**132.2**	**1.4**			
8	[CSU-3-NH_3_]OTf	2.0	1.0	366.9	350.5	3.8	60.7	89.1	98.9
				**293.5**	**280.4**	**3.0**			

a [Cat.] = 0.01 mM; electrolysis time, 1 h; *E*_app_*vs.* Cp_2_Fe^+/0^; carbon cloth (1 cm^2^) as the working electrode; molar ratio of N_2_, N_2_H_4_, and H_2_ determined by taking the average of two tests for the electrolyte in CPC experiments, and the generation of these compounds in the control CPC experiment (Table S5) is subtracted. The maximum relative errors of 1.5%, 2.2% and 3.5% for production of H_2_, N_2_H_4_ and N_2_.

b Charge passed in CPC experiments in 1 h.

c FE_N_2_H_4__ = *n*_N_2_H_4__/*Q* × 100%.

d *S*_N_2_H_4__ = *n*_N_2_H_4__/(*n*_N_2_H_4__ + *n*_N_2_H_4__) × 100%.

For complex CSU-3, the applied potential (*E*_app_) is fixed at 0.2 V (entry 1, 2) to only generate Ru^III^ species, and as expected, in CV studies, no oxidation products of ammonia are detected. When holding the *E*_app_ at 1.0 V for a low concentration ammonia solution (0.2 M) for 1 h, the gas products of H_2_ (113.1 μmol, 141.4 equiv. based on Ru) and N_2_ (0.8 μmol, 1 equiv. based on Ru) in the headspace and N_2_N_4_ (112.2 μmol, 140.3 equiv. based on Ru) in the electrolyte solution are determined (entry 3). When the concentration of ammonia is increased to 2.0 M, the catalytic efficiency of CSU-3 is approximately doubled. In addition, the selectivity of N_2_H_4_ formation and Faraday efficiency (FE) is almost unchanged with changes in ammonia concentration, maintaining a level of 85.6 and 84.7%, respectively. This indicates that NH_3_ is possibly involved in the N_2_H_4_ formation step *via* an ammonia nucleophilic attack mechanism and/or N_2_H_4_ release *via* N_2_H_4_-by-NH_3_ substitution.

In the [CSU-3-NH_3_]OTf catalyst system, only N_2_H_4_ as an anodic product is generated at low electrolytic potential (0.2 V) to only generate Ru^III^ species as the intermediate (entry 5, 6), which is very similar to the results for CSU-1, CSU-2 and [Ru(K^3^-*N*,*N*′,*N*′′-dpp)(bpy)(L)]·PF_6_.^25^ This suggests that a bimolecular coupling mechanism of ruthenium amide is possibly involved. Holding the *E*_app_ at 1.0 V (Ru^IV^ species generated at this potential), the catalytic efficiency is greatly enhanced (entry 7, 8). TOF_N_2_H_4__, FE_N_2_H_4__, and *S*_N_2_H_4__ reach 350.5 s^−1^, 87.9% and 98.9%, respectively.

### Mechanism

**Fig. 3:**
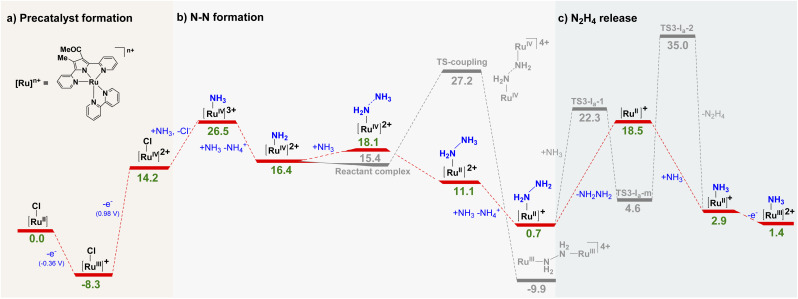
The possible mechanism of AO catalyzed by CSU-3. (a) Precatalyst formation, (b) N–N formation, and (c) N_2_H_4_ release. The free energy changes (Δ*G*) are presented in the individual reaction steps in kcal mol^−1^, with the calculated potentials in parentheses *versus* Cp_2_Fe^+/0^ in CH_3_CN.

The full mechanism of the AO reaction catalysed by CSU-3 is proposed by theoretical calculations. As shown in Fig. 3a, CSU-3 is firstly oxidized to [Ru^III^–Cl]^+^ (Δ*G* = −8.3 kcal mol^−1^). The corresponding calculated Ru^III/II^ redox potential is −0.36 V. Direct Cl-by-NH_3_ substitution of [Ru^III^–Cl]^+^ and CSU-3 to generate [Ru^III^–NH_3_]^2+^ and [Ru^II^–NH_3_]^+^, respectively, is unfavourable due to the high energy barrier (Fig. S25†). For example, two possible substitution pathways, namely the concerted associative pathway (*I*_a_) and dissociative pathway (*D*), are considered. The energy barriers of the *I*_a_ pathway (Δ*G*^‡^ = 26.2 kcal mol^−1^) and *D* pathway (Δ*G*^‡^ = 24.7 kcal mol^−1^) are high enough to hinder direct Cl-by-NH_3_ substitution of [Ru^III^–Cl]^+^, which is in agreement with what is observed in CV studies and synthetic experiments. A subsequent oxidation of [Ru^III^–Cl]^+^ generates [Ru^IV^–Cl]^2+^ (Δ*G* = 22.5 kcal mol^−1^, *E*_cal._ = 0.98 V). The subsequent Cl-by-NH_3_ substitution of [Ru^IV^–Cl]^2+^ to produce [Ru^IV^–NH_3_]^3+^ is an endergonic step with Δ*G* of = 12.3 kcal mol^−1^). Subsequently, deprotonation of [Ru^IV^–NH_3_]^3+^ affords ruthenium(iv)-imido complex [Ru^IV^–NH_2_]^2+^, which is a key intermediate for N_2_H_4_ formation.

Complex [Ru^IV^–NH_2_]^2+^ is nucleophilically attacked by NH_3_ to produce terminal hydrazinium-ligated [Ru^II^–NH_2_NH_3_]^2+^, only overcoming an energy barrier of 1.7 kcal mol^−1^, followed by an energetically favourable deprotonation process to generate terminal hydrazine-ligated Ru^II^-intermediate [Ru^II^–NH_2_NH_2_]^+^ (Δ*G* = −17.4 kcal mol^−1^). Obviously, an N–N bond is readily formed *via* ammonia nucleophilic attack of [Ru^IV^–NH_2_]^2+^ of CSU-3 (Δ*G*^‡^ = 1.7 kcal mol^−1^), unlike 1 and CSU-2*via* ammonia nucleophilic attack of Ru^IV^-imide with higher barriers (Δ*G*^‡^ = 24.1 and 7.7 kcal mol^−1^, respectively). The single-site molecular catalytic pathway of CSU-3 is confirmed by the linear relationship between the catalytic current and concentration of ammonia and catalyst (Fig. S23 and S24†). Furthermore, the pathway of generating hydrazine-bridged bimetallic [Ru^III^–μ*–*N_2_H_4_–Ru^III^]^4+^*via* bimolecular N–N coupling [Ru^IV^–NH_2_]^2+^ (grey line in Fig. 3b) is excluded due to the high energetic barrier (Δ*G*^‡^ = 10.8 kcal mol^−1^).

N_2_H_4_/N_2_ selectivity is usually based on the hydrazine-ligated Ru^II^-intermediate, which could oxidize the ruthenium centre leading to hydrazine oxidation to generate N_2_,^8,17,19*a*,20*a*^ but also could cause N_2_H_4_-by-NH_3_ substitution to produce N_2_H_4_. As shown in Fig. 3c, N_2_H_4_ release through N_2_H_4_-by-NH_3_ substitution to generate [Ru^II^–NH_3_]^+^*via* the *I*_a_ and *D* pathways was considered. Compared to the *I*_a_ mechanism with two transition states with large energetic barriers (Δ*G*^‡^ = 21.6 and 30.4 kcal mol^−1^), the release of N_2_H_4_*via* the *D* pathway is more favourable due to the lower energetic barrier of 17.8 kcal mol^−1^, which is also lower than that in the CSU-2 catalytic AO system (Δ*G*^‡^ = 23.4 kcal mol^−1^).

Orbital interaction and electrostatic force between Ru and hydrazine in [Ru^II^–NH_2_NH_2_]^+^ play a key role in the stabilization of the binding of the dative ligand. The energy of the lowest unoccupied molecular orbital of [Ru^II^]^+^ from CSU-3 shows a higher value of −0.078 au compared to [Ru(trpy)(dmabpy)]^+^ from 1 (−0.093 au), indicating the relatively weaker Ru–N_2_H_4_ bond in [Ru^II^–NH_2_NH_2_]^+^, which is more labile (Fig. S26†). Meanwhile, natural population analysis shows that the partial charge at the ruthenium centre of [Ru^II^–NH_2_NH_2_]^+^ from CSU-3 is more positive than that from 1 (Table S6†), indicating that the influence of the electrostatic interaction is not as large as that of orbital interaction because N_2_H_4_ binds less strongly to the complex, where the partial charge at the ruthenium is larger. After N_2_H_4_-by-NH_3_ substitution, the formed [Ru^II^–NH_3_]^+^ is continuously oxidized to [Ru^III^–NH_3_]^2+^, and the catalytic cycle restarts. Obviously, except for the Cl-by-NH_3_ substitution in the precatalyst formation step, N_2_H_4_-by-NH_3_ substitution (or N_2_H_4_ release) is the rate-determining step for the catalytic oxidation of ammonia to hydrazine. According to the literature,^7^ one-electron metal-based oxidation of [Ru^II^–NH_2_NH_2_]^+^ to [Ru^III^–NH_2_NH_2_]^2+^ in the complex 1 catalytic system is calculated to be the most endergonic step (31.7 kcal mol^−1^) in the AO reaction. This electron transfer step seems to be a key ingredient in NH_3_ conversion into N_2_. Hence, we believe that this very thermodynamically demanding step is the possible reason that N_2_ generation is unfavourable in the CSU-3 catalytic system.

## Conclusions

In summary, a mononuclear ruthenium complex CSU-3 and its selective catalysis for ammonia oxidation is reported. The dpp_Me, COMe_^−^ as a pincer ligand coordinates to the ruthenium center, and the Cl^−^ ligand occupies the axial position. The redox potential of Ru^III/II^ in CSU-3 is negatively shifted to about 0.14 V and 0.61 V due to the electron donor nature of the dipyridylpyrrole ligand and the Cl^−^ axially coordinated ligand, compared to structurally analogous complex 1 and CSU-1, respectively. Complex CSU-3 could selectively catalyze the oxidation of ammonia to generate N_2_H_4_ as a dominant product *via* ammonia nucleophilic attack of the ruthenium(iv) imide forming a N–N bond, followed by an N_2_H_4_-by-NH_3_ substitution, which is significantly distinguished from the structurally analogous 1 producing N_2_*via* an ANA mechanism and is also different from CSU-1 and CSU-2 bearing similar hemilabile dipyridylpyrrolide ligands, which more efficiently give the N_2_H_4_ product *via* a bimolecular coupling mechanism of the ruthenium(iii)-iminyl radical. DFT calculation indicates that N_2_H_4_ release is the rate-determining step for NH_3_-to-N_2_H_4_ conversion catalysed by CSU-3. The weak orbital interaction between the HOMO of N_2_H_4_ and the LUMO of *D* (the LUMO is the orbital that the dissociated N_2_H_4_ binds to) in the CSU-3 catalyst system may be the main reason why N_2_H_4_ is more easily released than in the 1 catalyst system.

## Data availability

All data included in this paper are available upon request by contact with the corresponding author.

## Author contributions

X.-Y. Yi designed research; G. Chen, X.-L. Ding, P. He, and T. Cheng. performed research; G. Chen., X.-L. Ding, Y. Chen, J. Lin, X. Zhang, S. Zhao and N. Qiao analyzed data; G. Chen and X.-Y. Yi wrote the paper.

## Conflicts of interest

There are no conflicts to declare.

## Supplementary Material

SC-016-D4SC02360A-s001

SC-016-D4SC02360A-s002

## References

[cit1] Tran D. T., Nguyen T. H., Jeong H., Tran P. K. L., Malhotra D., Jeong K. U., Kim N. H., Lee J. H. (2022). Nano Energy.

[cit2] Jiang L., Fu X. (2021). Engineering.

[cit3] Elishav O., Mosevitzky Lis B., Miller E. M., Arent D. J., Valera-Medina A., Grinberg Dana A., Shter G. E., Grader G. S. (2020). Chem. Rev..

[cit4] Liu H. Y., Lant H. M. C., Cody C. C., Jelusic J., Crabtree R. H., Brudvig G. W. (2023). ACS Catal..

[cit5] Dunn P. L., Cook B. J., Johnson S. I., Appel A. M., Bullock R. M. (2020). J. Am. Chem. Soc..

[cit6] Barona M., Johnson S. I., Mbea M., Bullock R. M., Raugei S. (2022). Top. Catal..

[cit7] Najafian A., R Cundari T. (2019). J. Phys. Chem. A.

[cit8] Habibzadeh F., Miller S. L., Hamann T. W., Smith III M. R. (2019). Proc. Natl. Acad. Sci. U. S. A..

[cit9] Nakajima K., Toda H., Sakata K., Nishibayashi Y. (2019). Nat. Chem..

[cit10] Dunn P. L., Johnson S. I., Kaminsky W., Bullock R. M. (2020). J. Am. Chem. Soc..

[cit11] Holub J., Vereshchuk N., Sanchez-Baygual F. J., Gil-Sepulcre M., Benet-Buchholz J., Llobet A. (2021). Inorg. Chem..

[cit12] Trenerry M. J., Wallen C. M., Brown T. R., Park S. V., Berry J. F. (2021). Nat. Chem..

[cit13] Jacob S. I., Chakraborty A., Chamas A., Bock R., Sepunaru L., Ménard G. (2023). ACS Energy Lett..

[cit14] Beiler A. M., Denisiuk A., Holub J., Sánchez-Baygua F. J., Gil-Sepulcre M., Ertem M. Z., Moonshiram D., Piccioni A., Llobet A. (2023). ACS Energy Lett..

[cit15] Feng S., Chen J., Wang R., Li H., Xie J., Guo Z., Lau T.-C., Liu Y. (2024). J. Am. Chem. Soc..

[cit16] Zott M. D., Garrido-Barros P., Peters J. C. (2019). ACS Catal..

[cit17] Li Y., Chen J.-Y., Miao Q., Yu X., Feng L., Liao R.-Z., Ye S., Tung C.-H., Wang W. A. (2022). J. Am. Chem. Soc..

[cit18] Liu L., Johnson S. I., Appel A. M., Bullock R. M. (2024). Angew. Chem., Int. Ed..

[cit19] Ahmed M. E., Raghibi Boroujeni M., Ghosh P., Greene C., Kundu S., Bertke J. A., Warren T. H. (2022). J. Am. Chem. Soc..

[cit20] Stephens D. N., Szilagyi R. K., Roehling P. N., Arulsamy N., Mock M. T. (2023). Angew. Chem., Int. Ed..

[cit21] Toda H., Kuroki K., Kanega R., Kuriyama S., Nakajima K., Himeda Y., Sakata K., Nishibayashi Y. (2021). ChemPlusChem.

[cit22] Dunn P. L., Cook B. J., Johnson S. I., Appel A. M., Bullock R. M. (2020). J. Am. Chem. Soc..

[cit23] Li J., Zhang F., Xiong H., Cai Y., Zhang B. (2024). Sci. China: Chem..

[cit24] Stephens D. N., Mock M. T. (2024). Eur. J. Inorg. Chem..

[cit25] Chen G., He P., Liu C., Mo X.-F., Wei J.-J., Chen Z.-W., Cheng T., Fu L.-Z., Yi X. Y. (2023). Nat. Catal..

