# Supplementary material for: Understanding the factors governing the ammonia oxidation reaction by a mononuclear ruthenium complex
Source: Chem Sci. 2025 Mar 19;16(17):7573–8. doi: 10.1039/d4sc02360a (PMC11955915; doi:10.1039/d4sc02360a)
Supplement: SC-016-D4SC02360A-s001 [file SC-016-D4SC02360A-s001.pdf]

Supplementary Information (SI) for Chemical Science.
This journal is © The Royal Society of Chemistry 2025

Supplementary Information for

Understanding the factors governing the ammonia oxidation reaction by mononuclear ruthenium complex

Guo Chen,[a, b] Xiao-Lv Ding,[a] Piao He,[a] Tao Cheng,[a] Yang Chen,[c] Jian Lin,[c] Xi Zhang,[a] Shan Zhao,[a] Na Qiao,[a] and Xiao-Yi Yi*[a]

[a] *College of Chemistry and Chemical Engineering, Central South University, Changsha, Hunan 410083, People's Republic of China.*

[b] *School of Chemistry and Chemical Engineering, Southwest University, Chongqing, 400715, People's Republic of China.*

[c] *CAS Key Laboratory of Science and Technology on Applied Catalysis, Dalian Institute of Chemical Physics, Chinese Academy of Sciences, Dalian, 116023, People's Republic of China.*

* Corresponding authors: Fax: 86 731 88879616; Tel: 86 731 88879616;

E-mail address: xyyi@csu.edu.cn

Table of Contents:

Supplementary Methods
Supplementary Tables and Figures
Coordinates from Geometry Optimizations
Supplementary References

Supplementary Methods

All of the chemicals were obtained from J&K Scientific in China. Ultradry solvents were treated by Solvent Drying System (J. C. Meyer, USA). The UV-visible spectra were measured by spectrophotometer Cary 8454 (Agilent Technologies, USA). Infrared spectra (KBr) were recorded on Nicolet 6700 spectrometer FT-IR spectrophotometer (Thermo Fisher Scientific, USA). Electrochemical measurements were accomplished with a CHENHUA CHI660E (China). Gas quantifications were conducted with a GC-2014C gas chromatograph (Shimadzu, Japan). XRD crystallography studies were carried out at the Bruker Smart ApexII CCD diffractometer (Mo Kα radiation) (Germany). The NMR spectra were recorded on a Bruker AVANCE (III) 400 M spectrometer (Germany). Time of Flight Mass Spectrometer (TOF-MS) was recorded by Bruker Daltonik GmbH (Germany). The experimental parameters were as follows: capillary temperature, 200 ^oC; capillary voltage, 3500 V; flow rate, 4 L/min; hexapole, 400 Vpp.

Synthesis of 1-(4-Methyl-2,5-di(pyridin-2-yl)-1H-pyrrol-3-yl)ethanone ($Hdpp_{Me, COMe}$).

The 1-(4-Methyl-2,5-di(pyridin-2-yl)-1H-pyrrol-3-yl)ethanone ($Hdpp_{Me, COMe}$) was prepared according to the literature.[1] The yield was 0.483 g (34.5%). The resulting solid recrystallized from ether to afford faint yellow flake crystals at -20 °C that were suitable for XRD analysis.

^{1}HNMR (400 MHz, $CDCl_3$): δ 10.56 (s, 1H), 8.60−8.6 (d, 1H), 7.63-7.71(m, 3H), 7.57-7.59(d, 1H), 7.16-7.19(t, 1H), 7.09-7.12(t, 1H), 2.51 (s, 3H), 2.45 (s, 3H).

^{13}C NMR (101 MHz, CDCl3): δ 199.73, 150.07, 149.73, 149.49, 149.39, 136.49, 136.46, 132.57, 129.19, 126.20, 32.02.

IR (KBr, cm^{-1}): 3431 (s), 3109 (w), 3051 (w), 2995 (w), 1658 (s), 1585 (s), 1550 (w), 1350 (m), 1292 (m), 1248 (m), 1217 (m), 1176 (m), 1153 (m), 1112 (m) , 1053 (m) ,997 (w) , 941 (m) , 791 (s) , 739 (m) , 704 (m) , 669 (m) , 613 (m).

ESI-MS (in CH_3CN): *m/z* calcd. (found) for $[Hdpp_{Me, COMe} + H]^+$: 278.1288 (278.1194).

Synthesis of $[Ru(dpp_{Me, COMe})(bipy)(Cl)]$ (**CSU-3**).

The *cis*-$[Ru(dmso)_4(Cl)_2]$[2] was prepared according to the literature. In the N_2 atmosphere, a mixture of *cis*-$[Ru(dmso)_4(Cl)_2]$ (2.18 g, 4.5 mmol), $Hdpp_{Me, COMe}$ (1.42 g, 5.1 mmol), bpy (702 mg, 4.5 mmol) and dry Et_3N (~ 5 mL) in dry toluene (100 mL) was refluxed overnight. The suspension solution was filtered. The crude solid was washed by toluene (3 × 10 mL) and H_2O (3 × 10 mL). The resulted solid was redissolved in CH_2Cl_2, and recrystallized by addition hexane to afforded darkish red solid (1.13 g). This red solid was dissolved in MeOH (10 mL) and refluxed for 5 d. The solvent of resulted solution was evaporated by vacuum. The solid was dissolved in CH_2Cl_2 and run column chromatography (CH_2Cl_2:CH_3OH=50:1). The first band was collected, and recrystallized by addition of hexane to afford purple-red solid as **CSU-3** pure enough. Yield: 589 mg (23%). The solid of **CSU-3** in CH_2Cl_2 was layered n-hexane to obtain block dark-red single crystals which were suitable for X-ray diffraction analysis.

^{1}H NMR (400 MHz, $CDCl_3$): δ 10.51 (d, 1H), 8.96 (d, 1H), 8.24 (d, 1H), 7.99 (d, 1H), 7.87

(t, 1H), 7.77 (s, 1H), 7.71 (t, 1H), 7.54 (d, 1H), 7.43 – 7.29 (m, 3H), 7.14 (s, 2H), 6.95 (dd, 1H), 6.49 (dd, 2H), 2.95 (s, 3H), 2.67 (s, 3H) ppm.
^{13}C NMR (101 MHz, $CDCl_3$): δ 192.47, 161.01, 160.63, 157.45, 153.88, 153.17, 152.36, 143.73, 135.77, 132.08, 131.16, 125.62, 124.98, 122.56, 121.58, 121.24, 119.78, 118.06, 53.46, 31.24, 13.39 ppm.
IR (KBr, cm^{-1}): 3444 (m), 1620 (w), 1591 (w), 1462 (w), 1427 (w), 1355 (m), 1259 (s), 1155 (s), 1029 (m), 983 (w), 947 (s), 756 (s), 621 (w).
ESI-MS (in CH_3CN): *m/z* calcd. (found) for [**CSU-3**]$^+$: 569.0555 (569.0282).
Elemental analysis: calcd. (found) for $C_{27}H_{22}ClN_5ORu\cdot(CH_2Cl_2)$ (%):

	C%	H%	N%
Found	51.70	3.80	10.87
Calcd.	51.42	3.70	10.71

Synthesis of [Ru(dpp$_{Me, COMe}$)(bipy)(NH_3)]OTf ([**CSU-3-NH$_3$](OTf)**.

A solution AgOTf (209 mg, 0.88 mmol) solution in CH_2Cl_2 (2 mL) was dropwise added into **CSU-3** (50 mg, 0.088 mmol) solution in CH_2Cl_2 (15 mL). The mixture solution was stirred for 2 h and the AgCl was removed off *via* filtration. The filtrate solution was bubbling NH_3 gas for 10 min, color of solution was changed from darkish purple to orange. The reaction solution was concentrated to 1 mL and recrystallized by addition of Et_2O to afforded orange solid as [**CSU-3-NH$_3$**](OTf). Yield: 16 mg (33 %).
^1H NMR (400 MHz, $CDCl_3$): δ 10.31 (s, 1H), 8.96 (d, 1H), 8.29 (d, 1H), 8.06 (d, 1H), 7.96 (dd, 2H), 7.66 (d, 1H), 7.59 – 7.49 (m, 2H), 7.44 (t, 2H), 7.31 (d, 1H), 7.22 (d, 1H), 7.00 (t, 1H), 6.63 (t, 2H), 2.85 (s, 3H), 2.69 (s, 3H), 2.47 (s, 3H) ppm.
^{13}C NMR (101 MHz, $CDCl_3$): δ 193.64, 160.63, 160.40, 159.01, 156.53, 153.94, 153.48, 153.08, 151.99, 143.09, 137.26, 136.86, 133.84, 133.13, 128.91, 127.16, 126.29, 125.20, 122.55, 122.15, 121.89, 121.15, 118.58, 31.31, 14.14 ppm.
IR (KBr, cm^{-1}): 3439 (m), 3093 (m), 3041 (m), 2962 (m), 2916 (m), 1612 (w), 1589 (s), 1496 (s), 1460 (m), 1419 (w), 1352 (m), 1261 (w), 1234 (w), 1153 (w), 1053 (w), 1018 (m), 983 (m), 943 (m), 771 (w), 748 (s), 727 (s), 700 (m), 650 (w), 540 (m).
ESI-MS (in CH_3CN): *m/z* calcd. (found) for [**CSU-3**]$^+$: 551.1135 (551.1135).
Elemental analysis: calcd. (found) for $C_{28}H_{25}N_6O_4F_3SRu$ (%):

	C%	H%	N%
Found	48.52	3.79	11.73
Calcd.	48.07	3.60	12.01

X-ray crystal structure determination.

The measurement of the crystals was performed using a Bruker Smart ApexII CCD diffractometer using graphite-mono-chromated Mo Kα radiation from an X-ray tube. The collected frames were processed with the software SAINT. The absorption correction was treated with SADABS.[3] Structures were solved using direct methods with SHELXS or SHELXT and refined against F^2 on all data by full-matrix least squares with SHELXL software packages.[4] The atomic positions of non-hydrogen atoms were refined with anisotropic parameters. The hydrogen atoms were introduced at their

geometric positions and refined as riding atoms. A summary of the crystallographic data and selected bond distances and angles for complexes are listed in Tables S1-4. CCDC 2329730 (for **CSU-3**) and 2330568 (for $Hdpp_{Me, COMe}$) contain the supplementary crystallographic data (Table S1-4) for this paper. These data can be obtained free of charge from The Cambridge Crystallographic Data Centre via www.ccdc.cam.ac.uk/data_request/cif.

Electrochemical measurements.

The typical sealed three-electrode cell was employed, including an Ag/AgCl electrode, a Pt wire, and a glassy carbon electrode (GCE), which were used as the reference electrode, the counter electrode, and the working electrode with a diameter of 3 mm, respectively. Cyclic voltammetry (CV) chronoamperometry and differential pulse voltammogram (DPV) were measured using 1 mM solutions of catalyst. The scan rate in all CV experiments was 0.1 V s^{-1}. If no otherwise specified, all potentials are converted into $E_{1/2}$ versus $Cp_2Fe^{+/0}$ in CH_3CN by adding −0.43 V to the measured potential.

Controlled potential coulometry (CPC) measurements.

The CPC experiments were carried in a sealed Schlenk electrolytic cell (internal volume of 276-283 mL) containing catalyst (0.01 mM), Bu_4NPF_6 (0.1 M) in ultradry MeCN (80 mL) in the presence of NH_3 (0.2 M or 2.0 M) under an Ar atmosphere. The carbon cloth (A = 1 cm^2), the platinum wire plate (A = 1 cm^2), and the Ag/AgCl electrode were used as the working electrode, counter electrode, and reference electrode, respectively. Applied potential of 0.2 V or 1.0 V vs. $Cp_2Fe^{+/0}$ were chosen. At given time, the gas products at the headspace of reactor were quantified by GC method, and the products in the electrolyte were quantified by N_2H_4, NO_2^-, NO_3^- and NH_3 test methods.[8-12]

Gas Chromatography (GC) methods.

Gas quantification was performed using a molecular sieve column attached to a thermal conductivity detector. Ar was used as the carrier gas. Mixture gas containing 2503.8 ppm H_2, 406.3 ppm N_2, 98.8 ppm O_2 are used as standard gases. Standard curves were generated by direct injection various amounts (100 μL, 200 μL, 300 μL, 400 μL, 500 μL, 600 μL, 700 μL, 800 μL, 900 μL, 1000 μL) of mixture gases using a gastight syringe (SGE Analytical Science). The calibration curve of H_2, N_2, O_2 and the corresponding linear equation is shown in Fig. S17.

DFT calculation.

The free energies of the reaction species were calculated using density functional theory (DFT) in the Gaussian 16 software package. The geometries of the catalyst models were fully optimized with the PBE0 functional[13] including the DFT-D3 dispersion correction with BJ-damping[14] and the def2-SV(P) basis set. All pertinent

spin states including low, intermediate, and high spin were evaluated for the complexes in their reactant, transition, and product states. The lowest free energy spin multiplicities were selected to calculate thermodynamics (ΔG) and free energy barriers ($\Delta G^{\ddagger}$) for the studied reactions. Single-point calculations for all stationary points were performed with the same functional and a larger basis set def2-TZVP basis set,[15, 16] to obtain more accurate free energies. The SMD implicit solvation model[17] was used to account for the solvation effect of MeCN (ε = 35.688). The redox potentials are calculated for all individual steps from ΔG of electron transfer reactions using the standard relationships, $E = -\Delta G/(nF)$, where −ΔG corresponds to electron transfer free energies, respectively. n is the number of electrons being transferred and F is the Faraday constant. The oxidation free energies were calculated relative to $Cp_2Fe \rightarrow [Cp_2Fe]^+ + e^-$ in the SMD-CH_3CN solvent, as shown below:

$LnRu^{n+} \rightarrow LnRu^{n+1} + e^-$ (eq. S4)

$[Cp_2Fe]^+ + e^- \rightarrow Cp_2Fe$ (eq. S5)

$L_nRu^{n+} + [Cp_2Fe]^+ \rightarrow LnRu^{n+1} + Cp_2Fe$ ΔG (eq. S6)

where L_n is ligand.

Supplementary Tables and Figures

Table S1. Crystallographic data of $Hdpp_{Me, COMe}$.

Compound	$Hdpp_{Me, COMe}$
CCDC	2330568
Empirical formula	$C_{17}H_{15}N_3O$
Formula weight	277.32
Crystal system	triclinic
Space group	*P*-1
a/Å	9.4605(5)
b/Å	9.5615(4)
c/Å	9.9418(4)
α/°	116.535(4)
β/°	108.263(5)
γ/°	100.865(4)
V/[Å^3]	704.62(6)
Z	2
ρ_{calcd} [g cm^{-3}]	1.307
μ [mm^{-1}]	0.670
F(000)	292.0
R_{int}	0.0620
^{a}GooF	1.064
$^bR_1$, $^cwR_2$ [I>2σ (*I*)]	0.0644/0.1808
R_1, wR_2 [all data]	0.0770/0.1808

^{a}GooF = $[\Sigma w(|F_o| - |F_c|)^2/(N_{obs} - N_{param})]^{½}$.

$^bR_1=\Sigma||F_o|-|F_c||/\Sigma|F_o|$. $^cwR_2$ $[(\Sigma w|F_o| -|F_c|)^2/\Sigma w^2|F_o|^2]½$.

Table S2. Solid-state structure, bond lengths (Å) and angles (°) of $Hdpp_{Me,\ COMe}$.

Solid-state structure of $Hdpp_{Me,\ COMe}$:

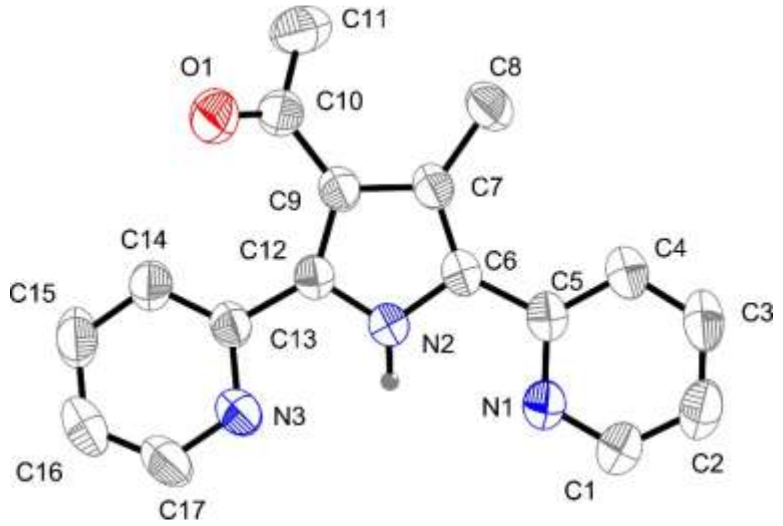

Bond lengths (Å) and angles (°) of $Hdpp_{Me,\ COMe}$:

$Hdpp_{Me,\ COMe}$			
Bond Distances(Å)			
N(1)-C(1)	1.328(2)	C(12)-C(13)	1.476(2)
N(1)-C(5)	1.344(2)	N(3)-C(13)	1.339(2)
C(5)-C(6)	1.464(2)	N(3)-C(17)	1.335(2)
N(2)-C(6)	1.369(2)	O(1)- C(10)	1.219(2)
N(2)-C(12)	1.348(2)		
Bond Angles (°)			
N(1)-C(1)-C(2)	124.3(2)	N(2)-C(12)-C(9)	106.73(14)
C(1)-N(1)-C(5)	118.12(16)	N(3)-C(13)-C(12)	124.45(16)
N(1)-C(5)-C(6)	115.07(14)	N(3)-C(13)-C(14)	121.76(16)
N(1)-C(5)-C(4)	120.66(17)	C(13)-N(3)-C(17)	118.09(17)
N(2)-C(6)-C(7)	107.24(14)	N(3)-C(17)-C(16)	123.3(2)
N(2)-C(6)-C(5)	117.59(15)	O(1)-C(10)-C(9)	121.11(17)
C(6)-N(2)-C(12)	111.85(14)	O(1)-C(10)-C(11)	119.18(18)
N(2)-C(12)-C(13)	117.46(14)		

Table S3. Crystallographic data of **CSU-3**.

Compound	**CSU-3**
CCDC	2329730
Empirical formula	$C_{27}H_{22}ClN_5ORu$
Formula weight	569.01
Crystal system	orthorhombic
Space group	$P2_12_12_1$
a/Å	9.4383(4)
b/Å	15.9847(7)
c/Å	18.7749(8)
α/°	90
β/°	90
γ/°	90
V/[Å^3]	2832.5(2)
Z	4
ρ_{calcd} [g cm^{-3}]	1.334
μ [mm^{-1}]	0.674
F(000)	1147.87
R_{int}	0.0536
[a]GooF	0.922
[b]R_1, [c]wR_2 [I>2σ (I)]	0.0541/0.1343
R_1, wR_2 [all data]	0.0886/0.1502

[a]GooF = $[\Sigma w(|F_o| - |F_c|)^2/(N_{obs} - N_{param})]^{½}$.

[b]$R_1=\Sigma||F_o|-|F_c||/\Sigma|F_o|$. [c]$wR_2$ $[(\Sigma w|F_o| -|F_c|)^2/\Sigma w^2|F_o|^2]^{½}$.

Table S4. Solid-state structure, bond lengths (Å) and angles (°) of **CSU-3**.

Solid-state structure of **CSU-3**:

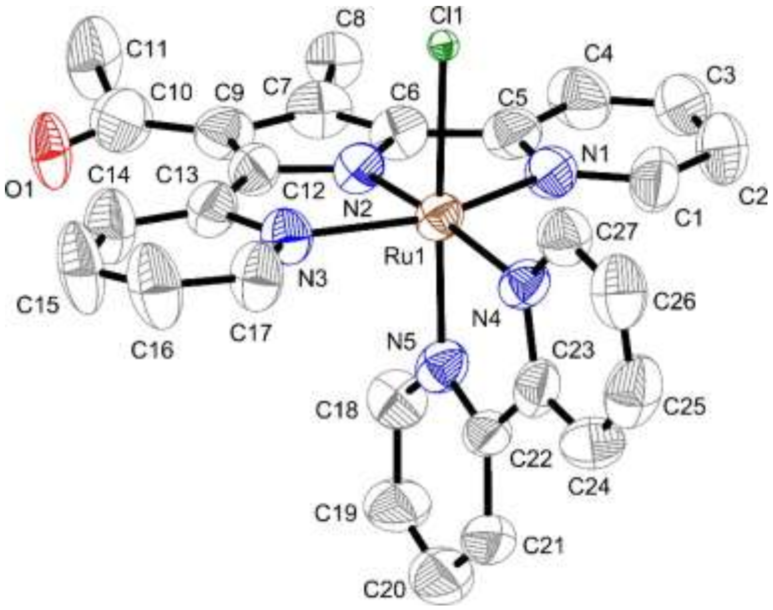

Bond lengths (Å) and angles (°) of **CSU-3**:

**CSU-3**			
Bond Distances(Å)			
Ru(1)-N(1)	2.107(5)	Ru(1)-N(5)	2.065(4)
Ru(1)-N(2)	1.907(4)	Ru(1)-Cl(1)	2.4297(14)
Ru(1)-N(3)	2.101(4)	O(1)-C(10)	1.184(11)
Ru(1)-N(4)	2.033(5)		
Bond Angles (°)			
N(1)-Ru(1)-N(2)	76.68(19)	N(4)-Ru(1)-N(5)	78.44(17)
N(1)-Ru(1)-N(3)	153.09(18)	Cl(1)-Ru(1)-N(1)	90.14(14)
N(1)-Ru(1)-N(4)	93.07(19)	Cl(1)-Ru(1)-N(2)	91.03(14)
N(1)-Ru(1)-N(5)	103.81(19)	Cl(1)-Ru(1)-N(3)	90.39(16)
N(2)-Ru(1)-N(3)	76.41(18)	Cl(1)-Ru(1)-N(4)	173.05(13)
N(2)-Ru(1)-N(4)	95.70(18)	Cl(1)-Ru(1)-N(5)	94.82(12)
N(2)-Ru(1)-N(5)	174.13(17)	O(1)-C(10)-C(9)	120.1(7)
N(3)-Ru(1)-N(4)	89.5(2)	O(1)-C(10)-C(11)	116.0(9)
N(3)-Ru(1)-N(5)	102.95(18)		

Table S5 | The control CPC experiment [a]

Entry	Cat.	$c(NH_3)$ (mol)	E_{app}	Time (h)	n_{H2} (μmol)	n_{N2H4} (μmol)	n_{N2} (μmol)
1	-	0.2	0.2	1	trace	trace	trace
2	-	2.0	0.2	1	trace	trace	trace
3	-	0.2	1.0	1	18.3	14.7	0.7
4	-	2.0	1.0	1	35.9	29.3	2.2

[a] Conditions: a Ag/AgCl electrode in saturated KCl solution as reference electrode, a Pt wire as counter electrode, a carbon cloth (1 cm^2) as working electrode.

Table S6 NPA of ruthenium (II) intermediates (D) from **CSU-3** and **1**.

	Ru^{II} intermediates (D)	
	**CSU-3**	**1**
Charge of Ru	0.61714	0.31047

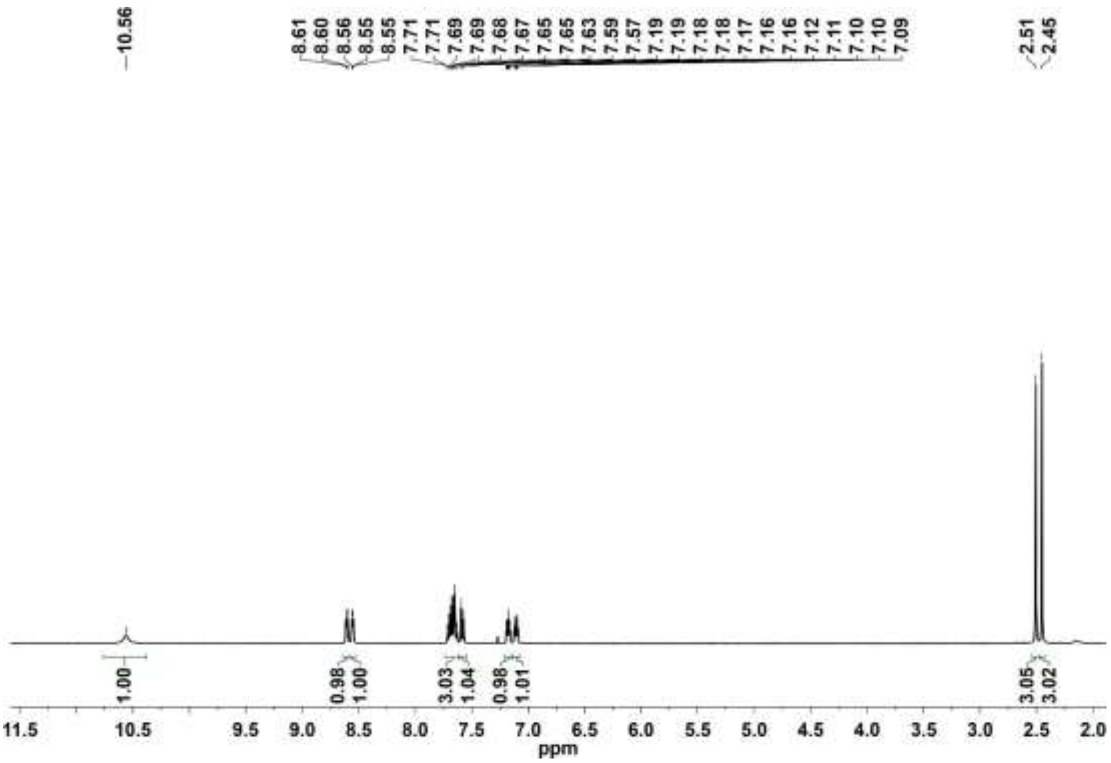

Fig. S1 ^{1}H NMR ($CDCl_3$) spectrum of $Hdpp_{Me, COMe}$.

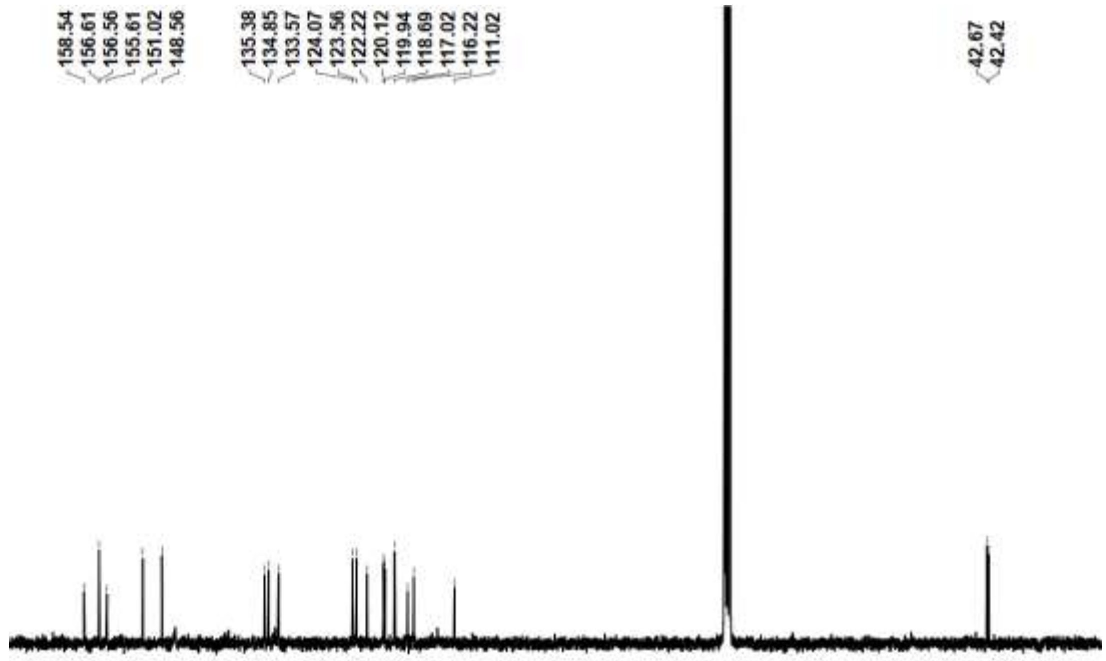

Fig. S2 ^{13}C NMR ($CDCl_3$) spectrum of $Hdpp_{Me, COMe}$.

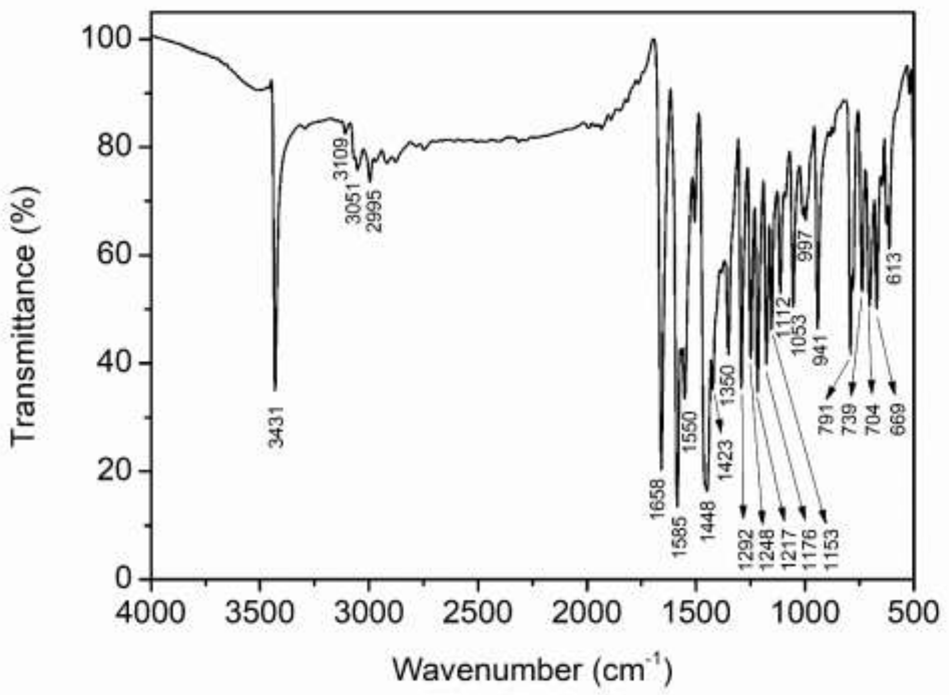

Fig. S3 IR spectrum of $Hdpp_{Me, COMe}$.

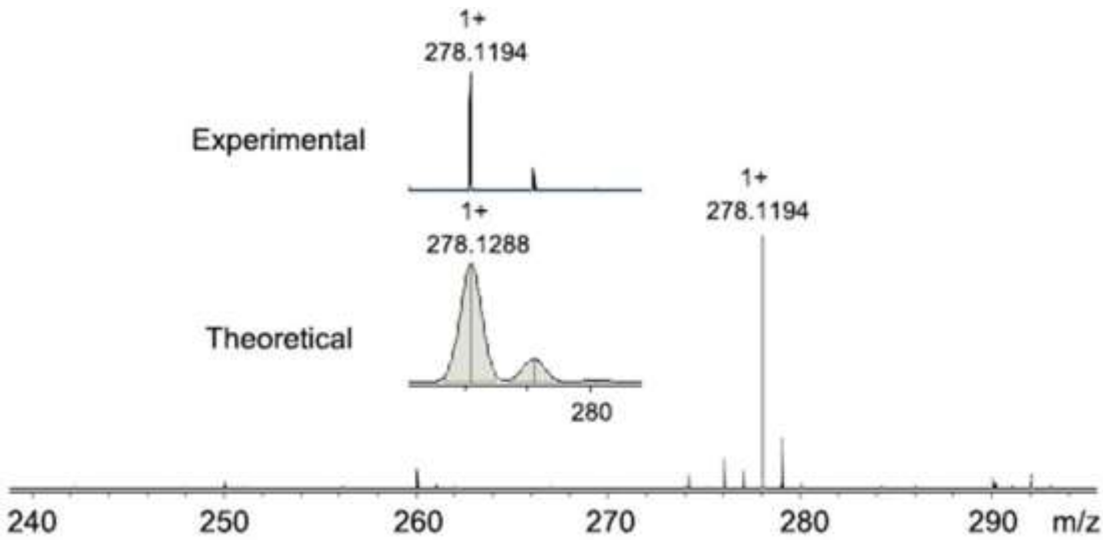

Fig. S4 ESI-MS spectrum of $Hdpp_{Me, COMe}$.

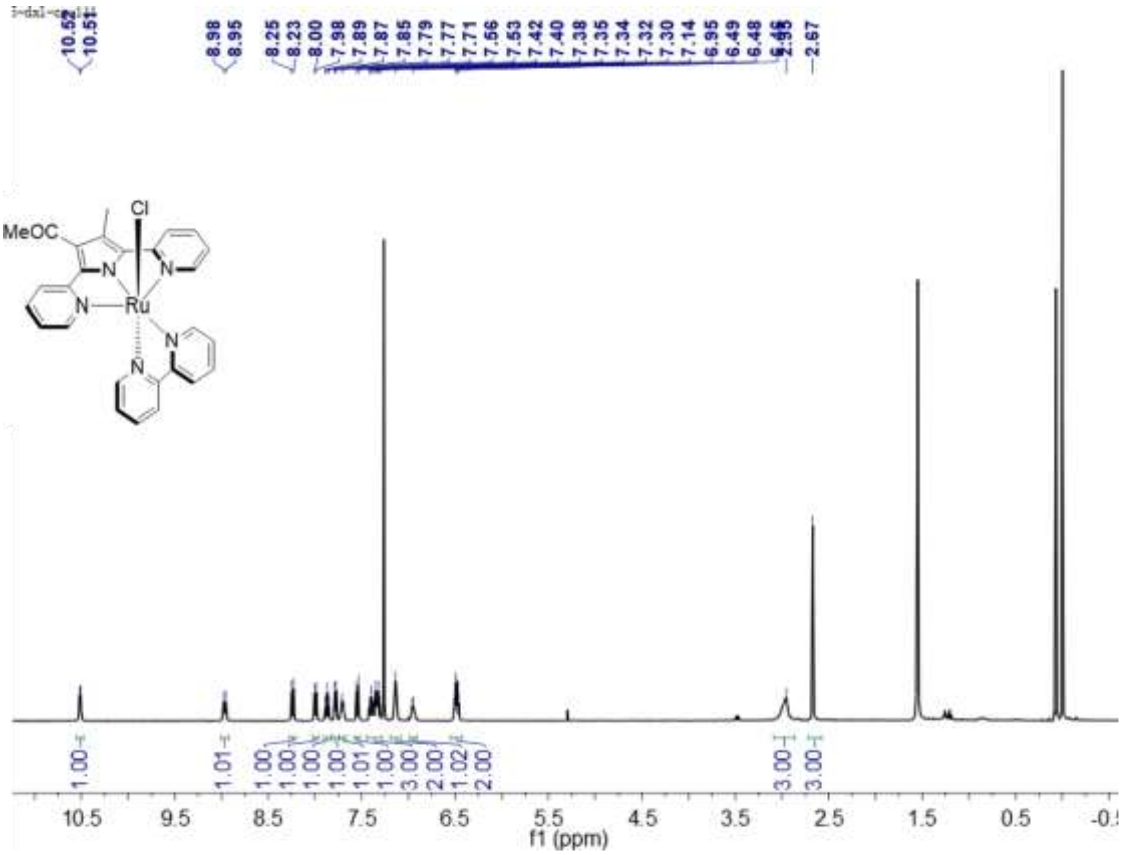

Fig. S5 ^{1}H NMR ($CDCl_3$) spectrum of **CSU-3**.

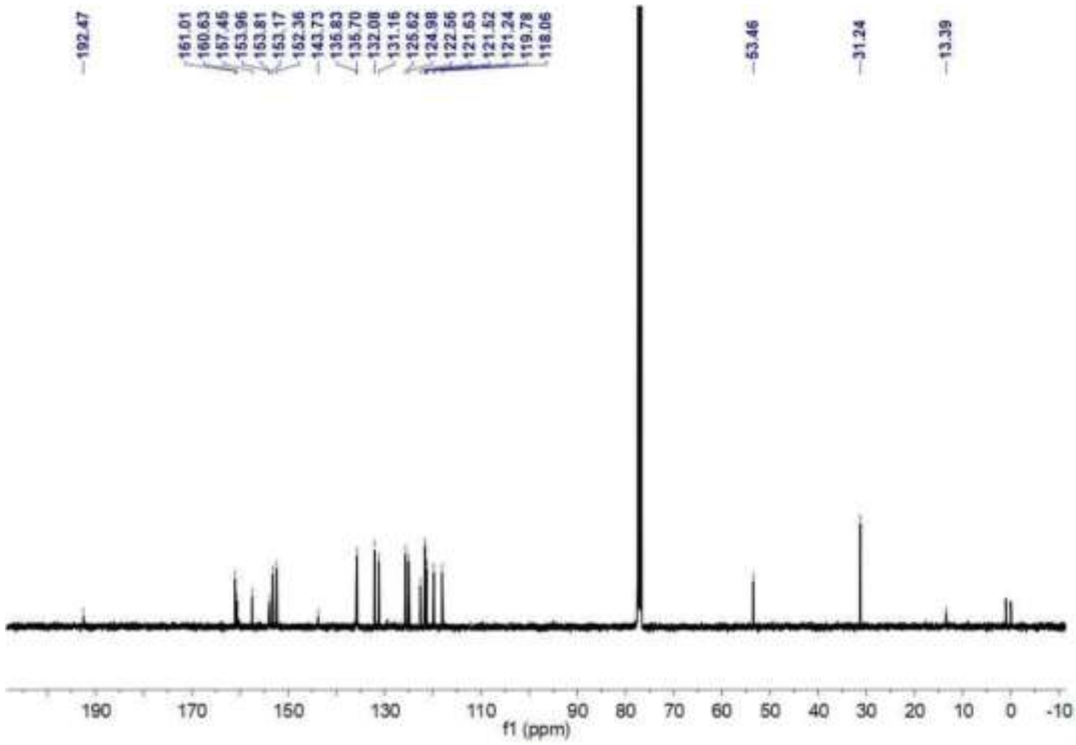

Fig. S6 ^{13}C NMR ($CDCl_3$) spectrum of **CSU-3**.

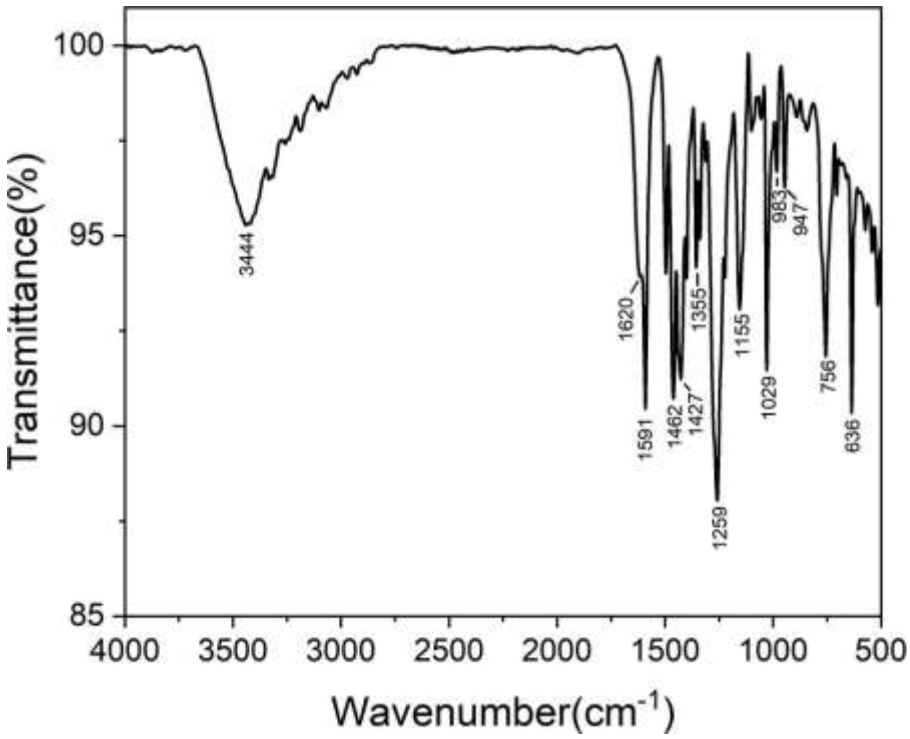

Fig. S7 IR spectrum and elemental analysis of **CSU-3**.

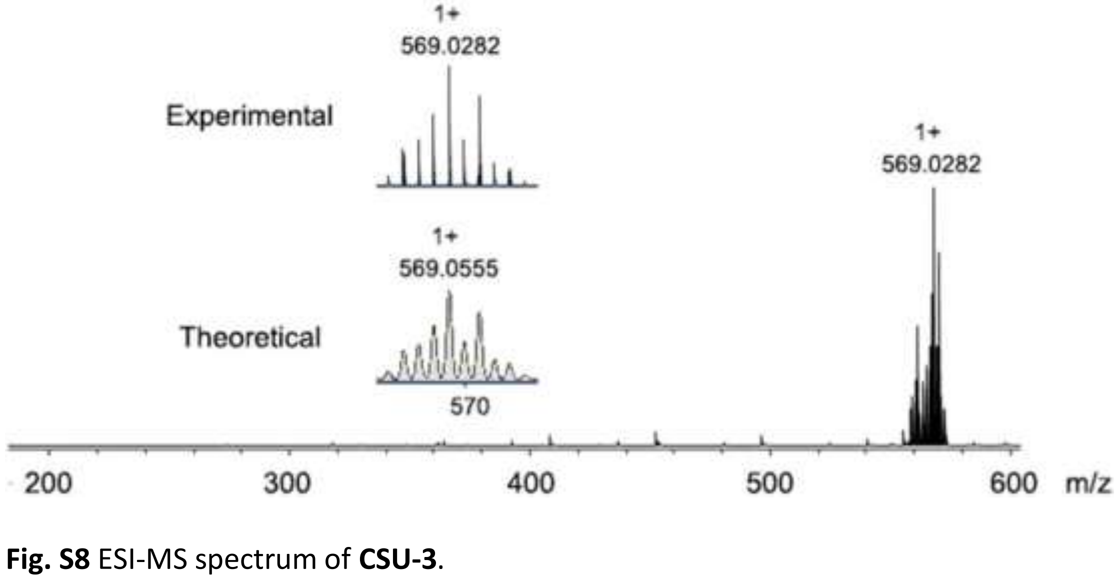

Fig. S8 ESI-MS spectrum of **CSU-3**.

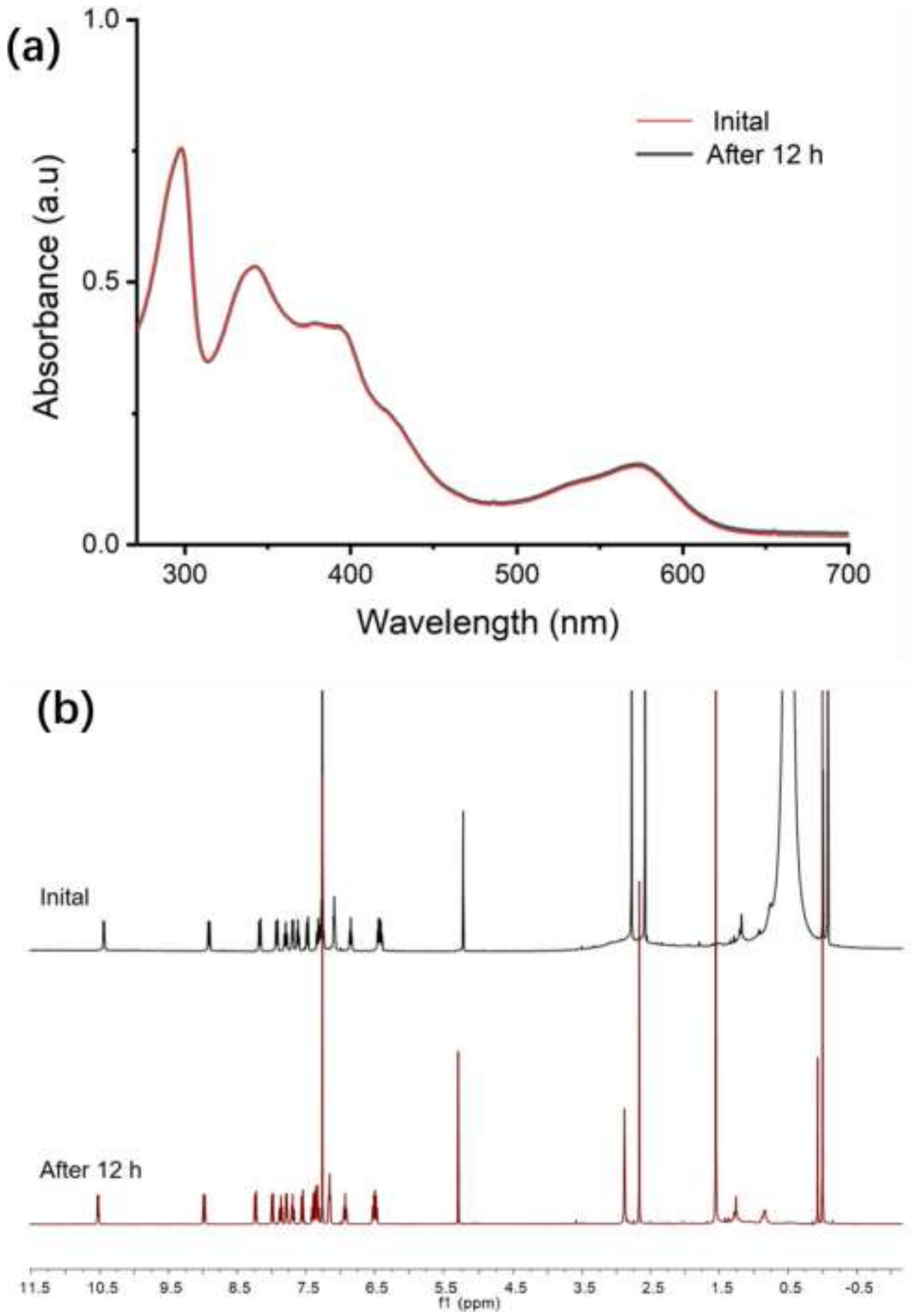

Fig. S9 (a) UV-vis spectra changes of **CSU-3**. red line: **CSU-3** solution in $CHCl_3$ before bubbling NH_3 (initial); black line: **CSU-3** solution in $CHCl_3$ stood undisturbedly for 12 h after 1 h NH_3 bubbling; (b) ^{1}H NMR spectra changes of **CSU-3**. black line: **CSU-3** solution in $CHCl_3$ before bubbling NH_3 (initial), red line: **CSU-3** solution in $CHCl_3$ stood undisturbedly for 12 h after 1 h NH_3 bubbling.

The negligible change indicates **CSU-3** can't react with NH_3.

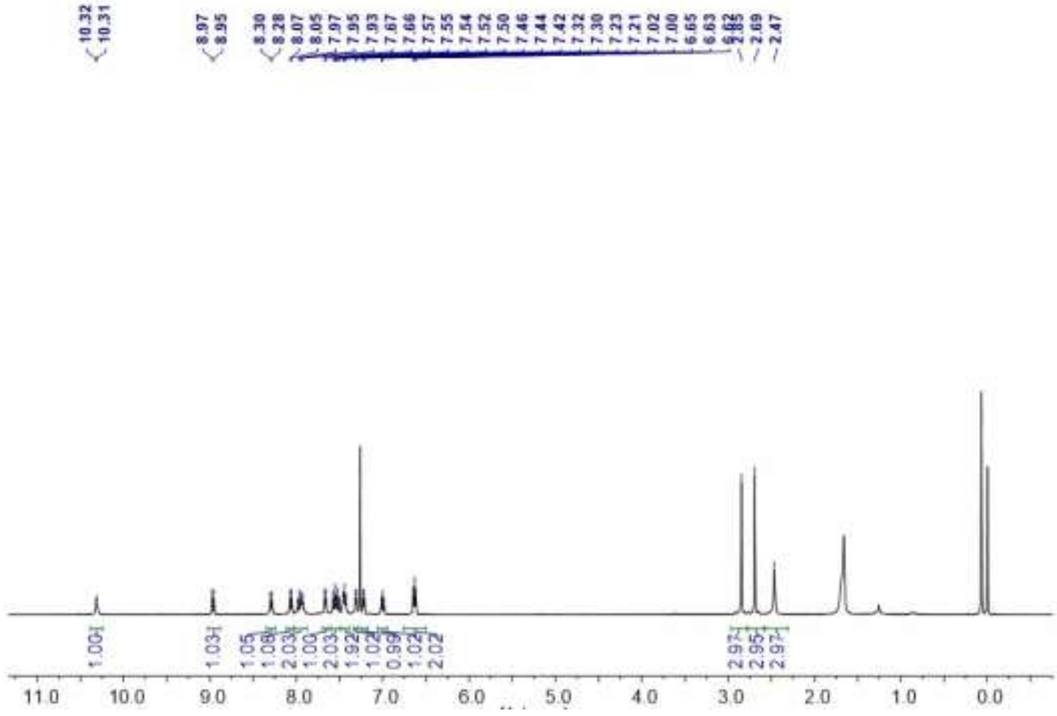

Fig. S10 ^{1}H NMR ($CDCl_3$) spectrum of **[CSU-3-NH$_3$]**OTf.

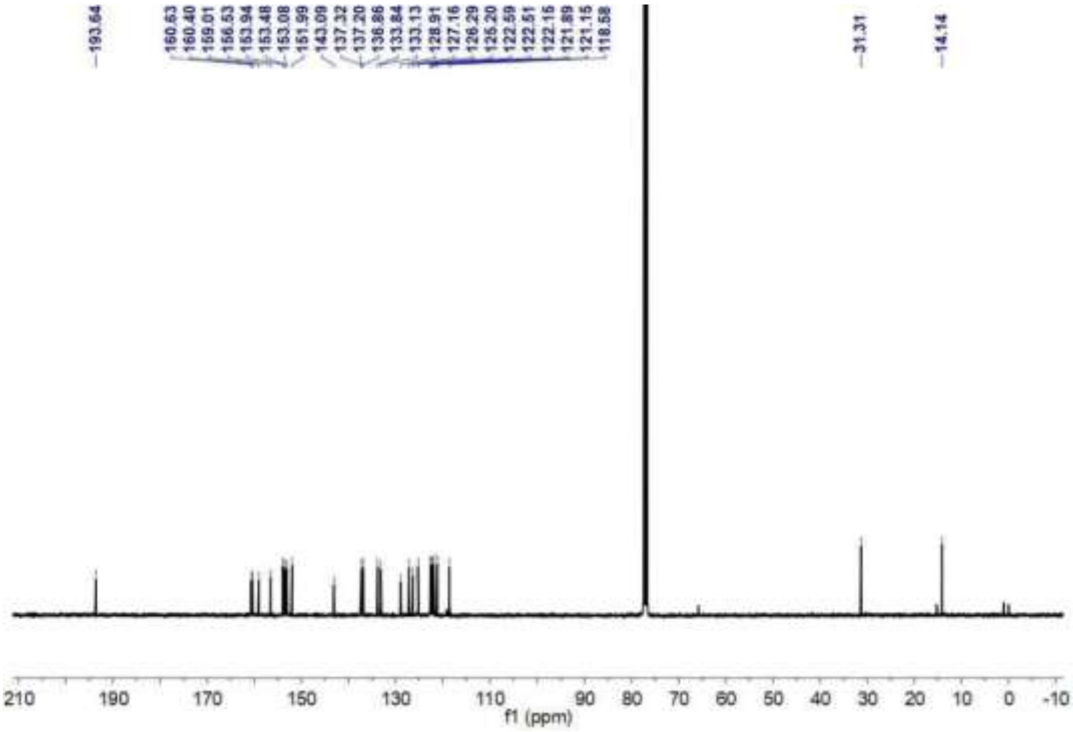

Fig. S11 ^{13}C NMR ($CDCl_3$) spectrum of **[CSU-3-NH$_3$]**OTf.

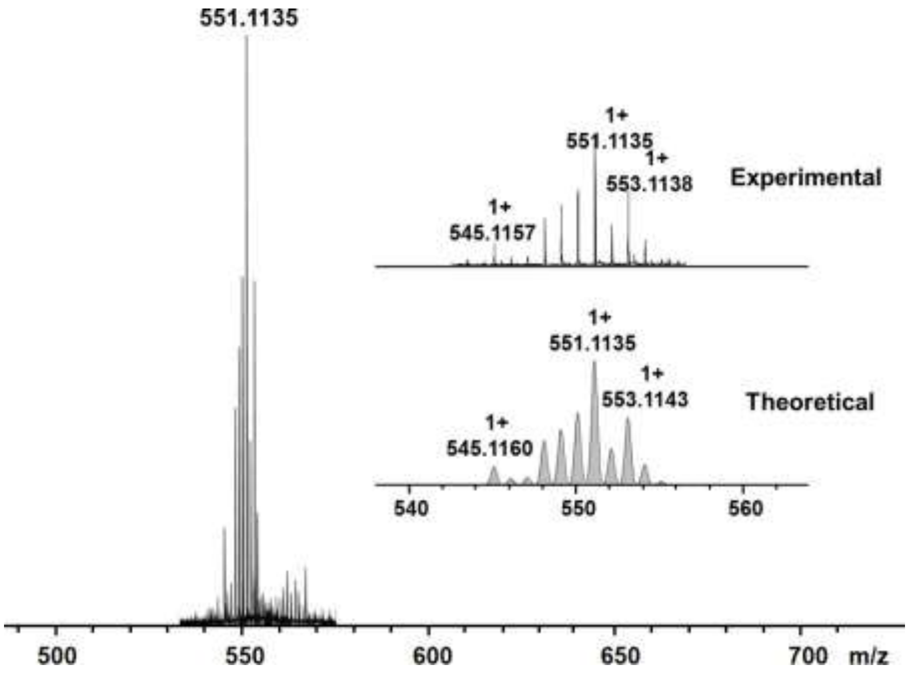

Fig. S12 ESI-MS spectrum of **[CSU-3-NH$_3$]**OTf.

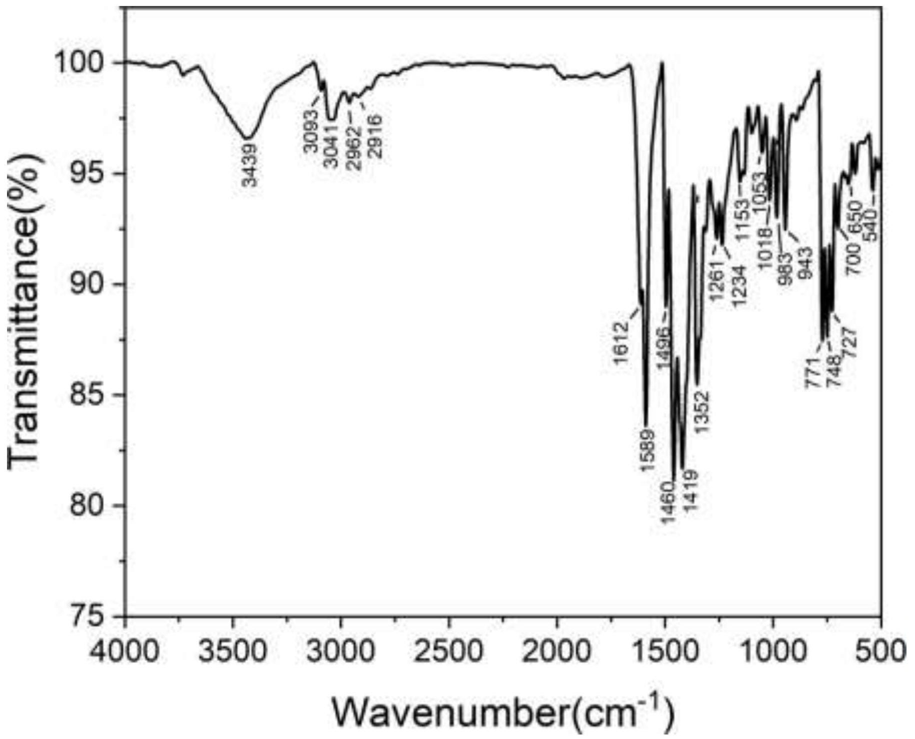

Fig. S13 IR spectrum of **[CSU-3-NH$_3$]**OTf.

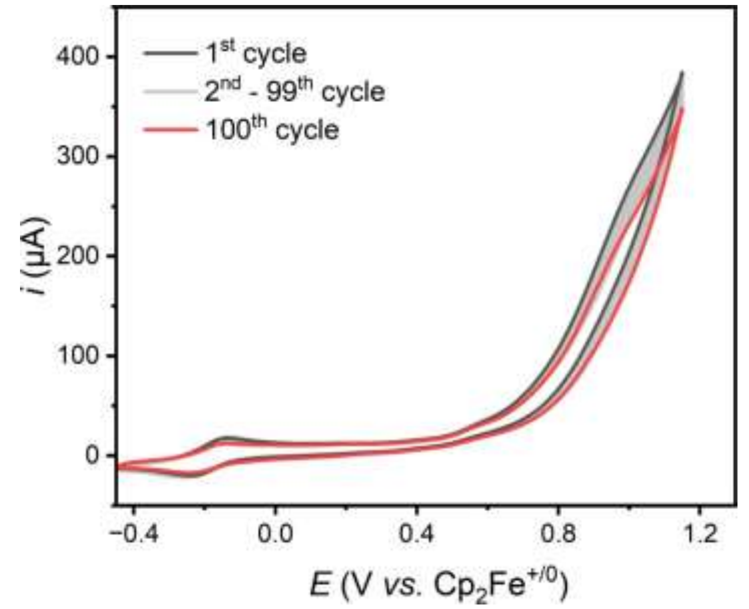

Fig. S14 Cycling stability of 1 mM **CSU-3** solution in MeCN in presence of 0.10 M NH_3.

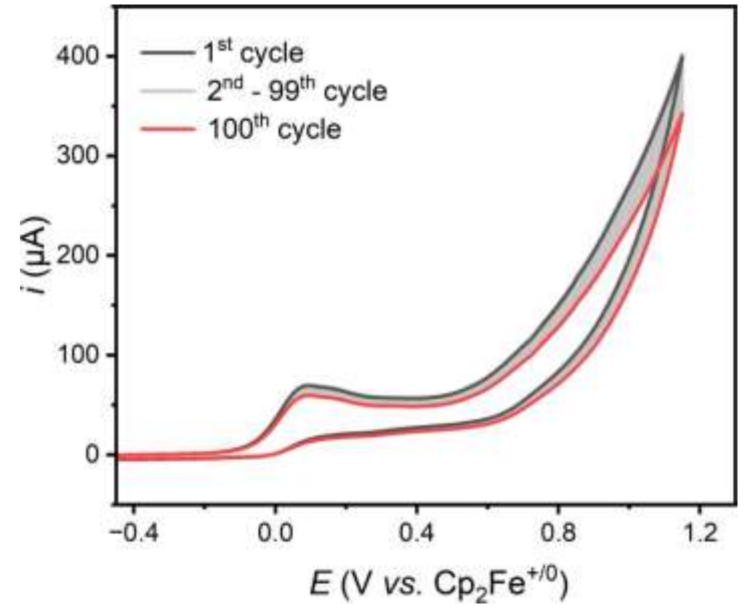

Fig. S15 Cycling stability of 1 mM [**CSU-3**]OTf solution in MeCN in presence of 0.10 M NH_3.

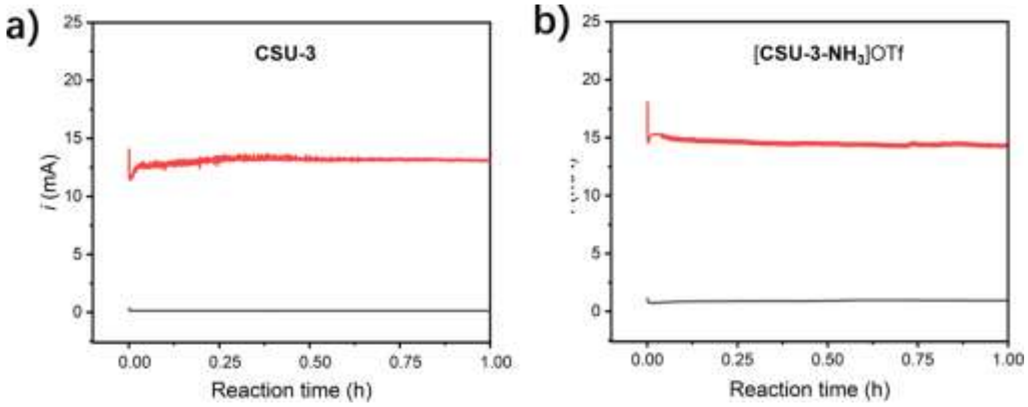

Fig. S16 the i/t plot of control CPC experiment of 2.0 M NH_3 in CH_3CN under conditions a) 0.01 M **CSU-3** using the fresh carbon cloth working electrode (red line, corresponding to Table 1 entry 4 in main text) and without **CSU-3** catalyst using the rinsed carbon cloth working electrode after CPC experiments with 0.01 M **CSU-3** (black line), b) 0.01 M [**CSU-3-NH$_3$**]OTf using the fresh carbon cloth working electrode (red line, corresponding to Table 1 entry 8 in main text) and without [**CSU-3-NH$_3$**]OTf catalyst using the rinsed carbon cloth working electrode after CPC experiments with 0.01 M [**CSU-3-NH$_3$**]OTf (black line).

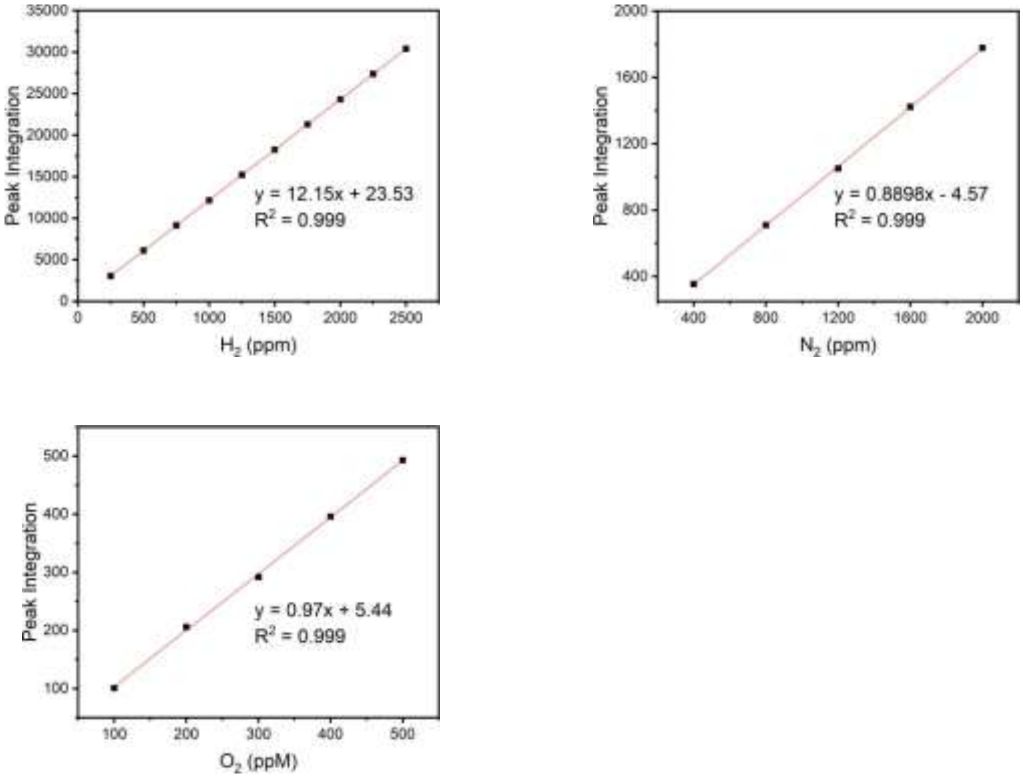

Fig. S17 The calibration curves of H_2, N_2, O_2.

Notably, the oxygen signal (aerobic contamination) in the cell of CPC experiments is inevitably observed. The aerobic contamination was estimated by assuming air as 4/1 mixture of N_2/O_2. Thus, the generated N_2 in the CPC experiments was quantified after correction of aerobic contamination estimated from the O_2 signal.

N_2H_4, NO_2^-, NO_3^- and NH_3 Test.

The testing methods for N_2H_4, NO_2^- $NO^-{}_3$and NH_3 in electrolytes refer to previously report.[8-12]

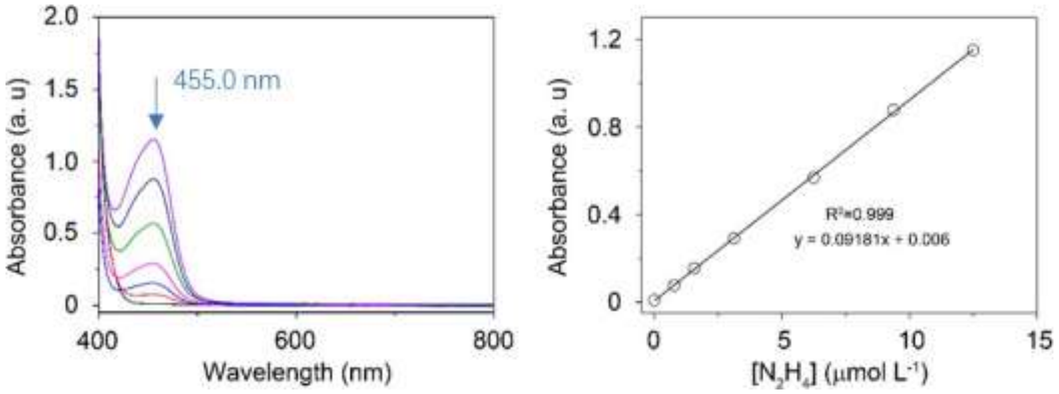

Fig. S18 Calibration curve used for calculation of N_2H_4 concentrations.

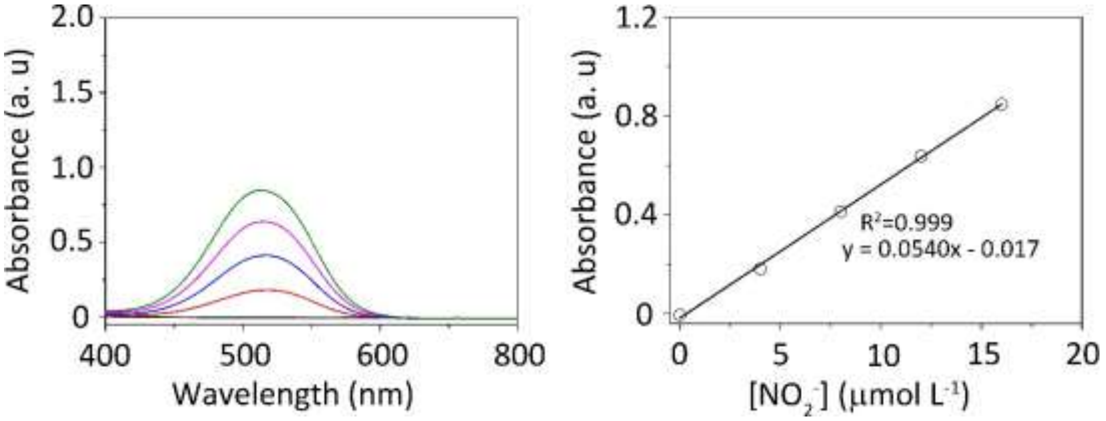

Fig. S19 Calibration curve used for calculation of NO_2^- concentrations.

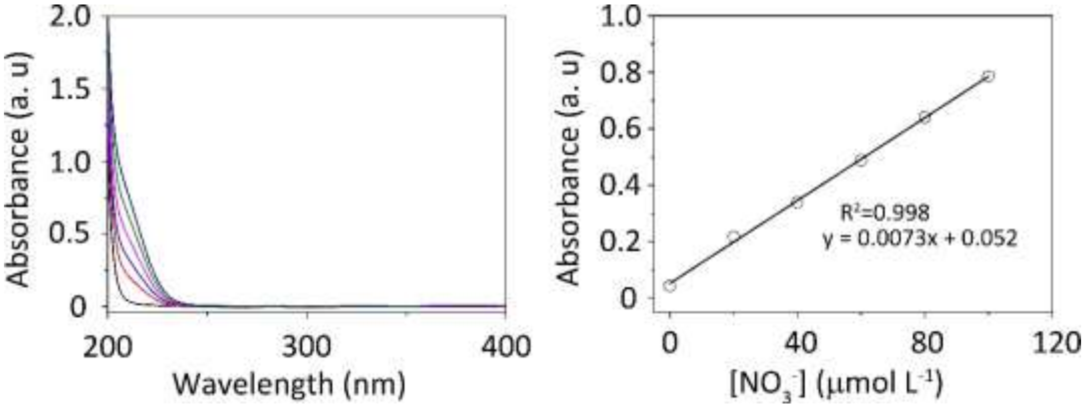

Fig. S20 Calibration curve used for calculation of NO_3^- concentrations.

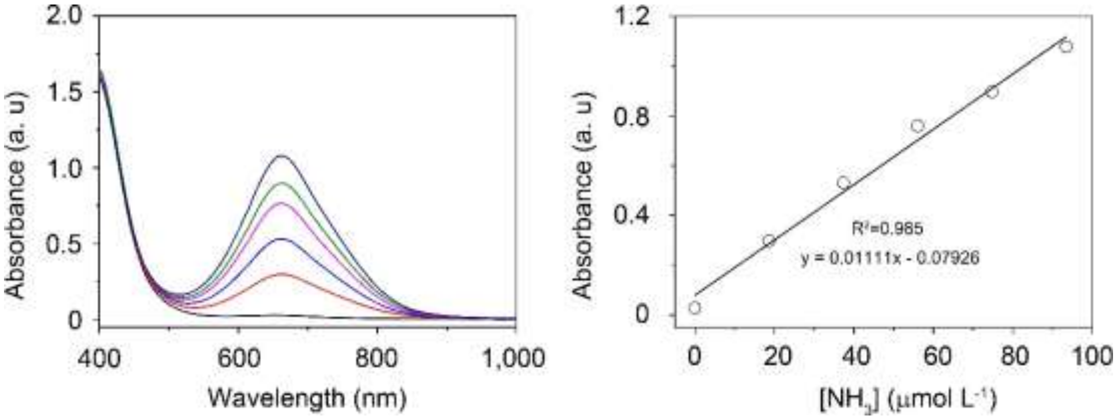

Fig. S21 Calibration curve used for calculation of NH_3 concentrations.

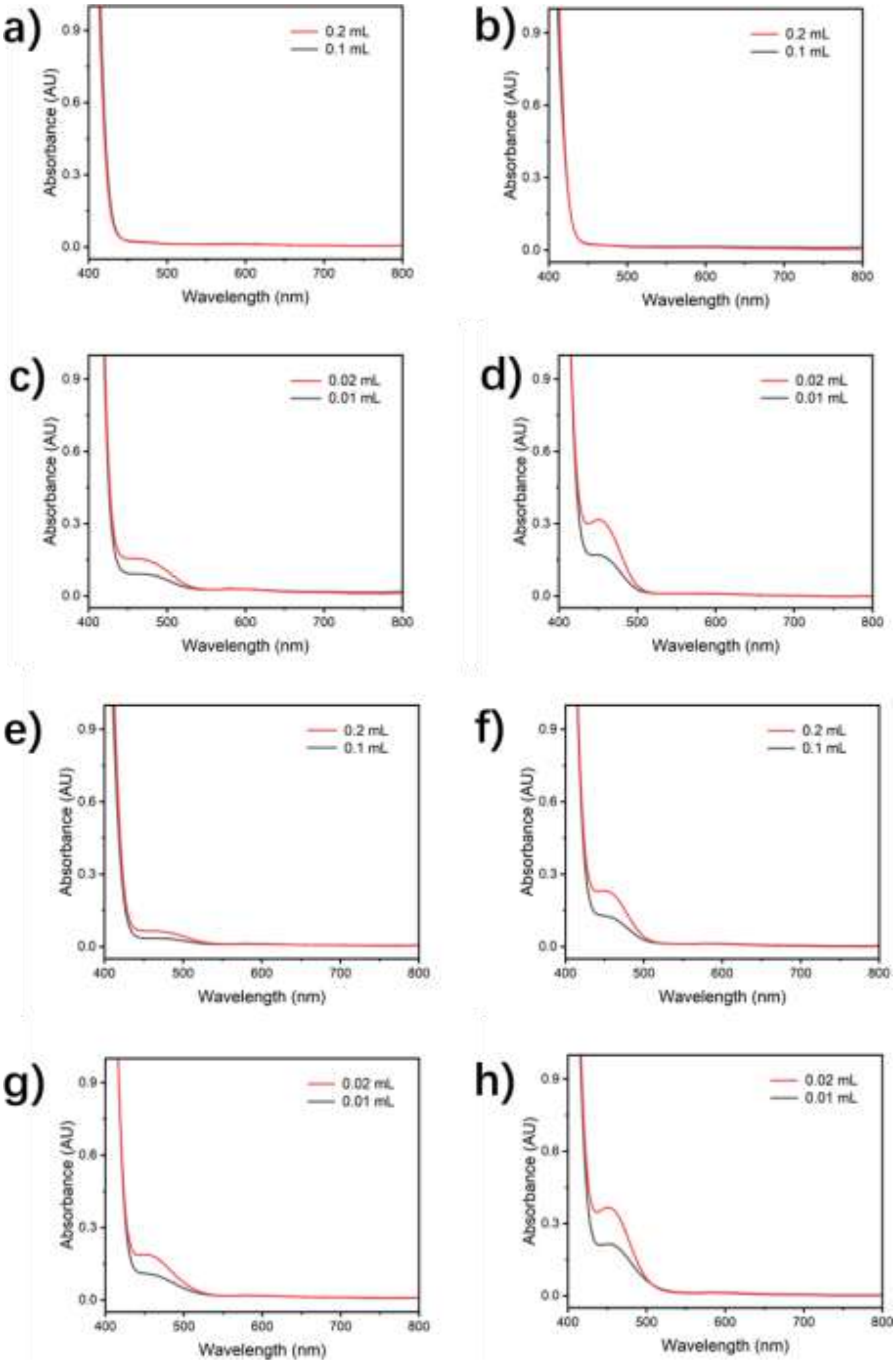

Fig. S22 Determination of N_2H_4 in the electrolyte of CPC experiment under conditions of a) **CSU-3** + 0.2 M NH_3 at 0.2 V; b) **CSU-3** + 2 M NH_3 at 0.2 V; c) **CSU-3** + 0.2 M NH_3 at 1 V; d) **CSU-3** + 2 M NH_3 at 1 V; e) [**CSU-3-NH$_3$**]OTf + 0.2 M NH_3 at 0.2 V; f) [**CSU-3-NH$_3$**]OTf + 2 M NH_3 at 0.2 V; g) [**CSU-3-NH$_3$**]OTf + 0.2 M NH_3 at 1 V; g) [**CSU-3-NH$_3$**]OTf + 2 M NH_3 at 1 V.

Procedure of quantification of N_2H_4 in CPC experiments: A given volume (0.10 mL, 0.20 mL or 0.010 mL, 0.020 mL) of electrolyte taken by high-accuracy syringe was mixed color reagent (2 mL) and then diluted to 10 mL. The resulting solution was shaken and allowed to stand for 20 min at room temperature, and then was analyzed with UV-vis spectroscopy using λ = 455.0 nm. Each sample is measured twice as shown in the figure above.

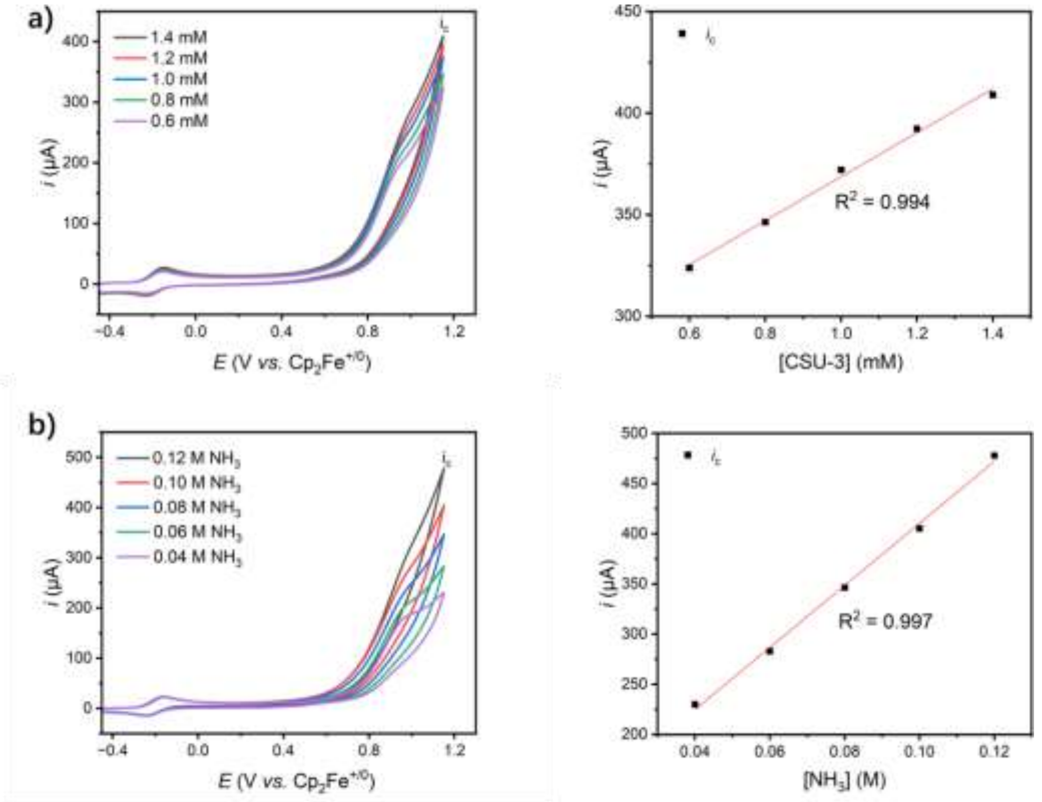

Fig. S23 a) the CVs of complex **CSU-3** in concentration range from 0.6 mM to 1.4 mM in the presence of 0.1 M NH_3. b) the CVs of 0.1 mM **CSU-3** in the presence of NH3 with concentration range from 0.04 M to 0.12 M, scan rate at 0.1 V s^{-1}.

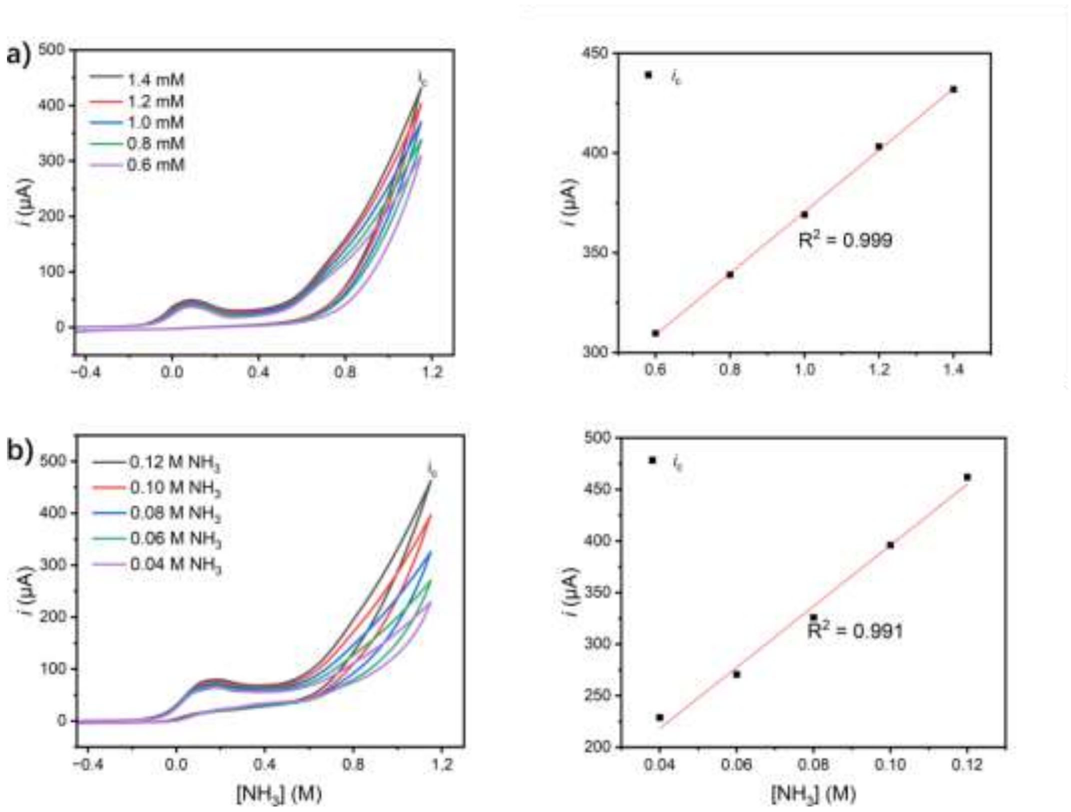

Fig. S24 a) the CVs of complex [**CSU-3-NH$_3$**]OTf in concentration range from 0.6 mM to 1.4 mM in the presence of 0.1 M NH_3. b) the CVs of 0.1 mM [**CSU-3-NH$_3$**]OTf in the presence of NH3 with concentration range from 0.04 M to 0.12 M, scan rate at 0.1 V s^{-1}.

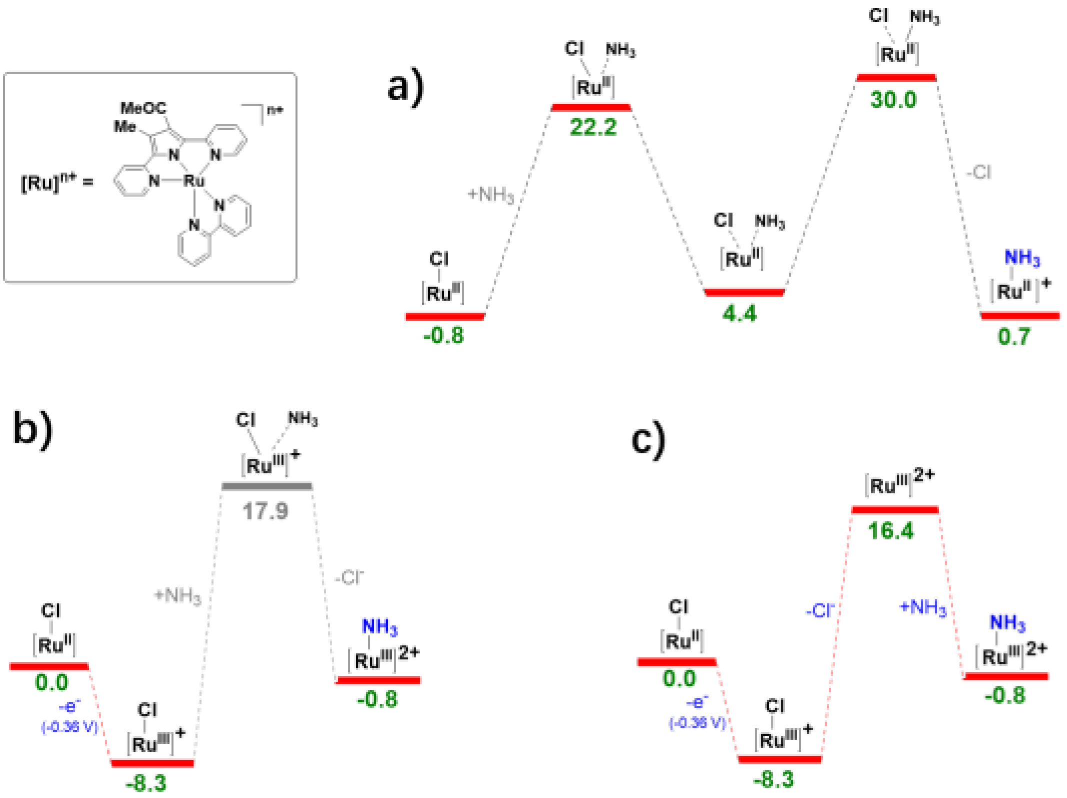

Fig. S25 a) direct Cl-by-NH_3 substitution of **CSU-3** to generate $[Ru^{II}\text{-}NH_3]^+$ *via* I_a pathway. b) direct Cl-by-NH_3 substitution of $[Ru^{III}\text{-}Cl]^+$ to generate $[Ru^{II}\text{-}NH_3]^+$ *via* I_a pathway; c) direct Cl-by-NH_3 substitution of $[Ru^{III}\text{-}Cl]^+$ to generate $[Ru^{II}\text{-}NH_3]^+$ *via* D pathway. The free energy changes (ΔG) are presented in individual reaction steps in kcal mol^{-1}, with the calculated potentials in parentheses versus $Cp_2Fe^{+/0}$ in CH_3CN.

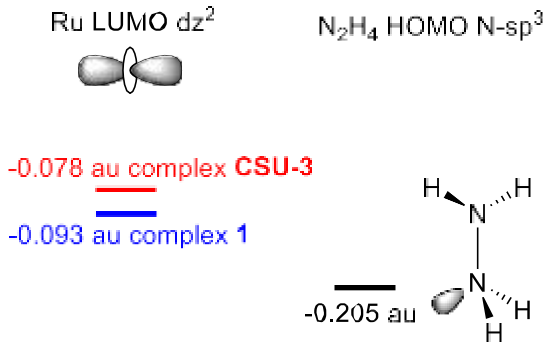

Fig. S26 Interacting orbitals of dative ligand N_2H_4 and ruthenium (II) intermediates (D) from **CSU-3** and **1**.

Coordinates from Geometry Optimizations

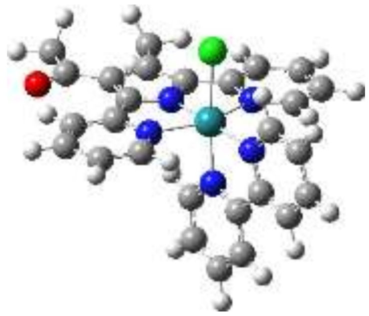

[Ru^{II}-Cl]
E = -1945.26185747
Charge = 0 Multiplicity = 1

Ru	-0.578937000	0.002653000	-0.487968000
N	-1.111015000	-0.123721000	1.467470000
N	-2.637544000	-0.288219000	-0.636417000
N	-0.426675000	2.095503000	-0.369519000
N	0.187821000	-1.949046000	-0.451979000
N	1.307630000	0.270223000	-0.198418000
C	1.862893000	1.497818000	-0.082597000
C	-3.338309000	-0.356903000	-1.778439000
H	-2.742234000	-0.255135000	-2.690671000
C	-0.246794000	-0.033575000	2.491596000
H	0.800454000	0.121854000	2.222473000
C	-2.893437000	-0.429006000	3.044002000
H	-3.955310000	-0.589261000	3.237181000
C	-2.430264000	-0.319097000	1.730201000
C	0.854709000	2.554201000	-0.162996000
C	-4.676740000	-0.587993000	0.595829000
H	-5.188394000	-0.675168000	1.555843000
C	3.255871000	1.328022000	0.086582000
C	-0.645792000	-0.132022000	3.816039000
H	0.100409000	-0.050917000	4.610151000
C	-5.398384000	-0.657936000	-0.589459000
C	-3.291781000	-0.404087000	0.546233000
C	-1.269458000	4.337975000	-0.335991000
H	-2.133407000	5.002506000	-0.410090000
C	3.492708000	-0.090834000	0.067971000
C	-1.996625000	-0.334764000	4.100475000
H	-2.347701000	-0.419156000	5.132349000
C	1.551223000	-2.020476000	-0.258083000
C	-1.438186000	2.962949000	-0.450486000
H	-2.427906000	2.526894000	-0.615244000
C	-0.522821000	-3.072254000	-0.574175000
H	-1.598362000	-2.941360000	-0.725780000
C	2.201568000	-0.713798000	-0.124660000
O	4.826918000	-2.026300000	0.181049000
H	4.743086000	2.420303000	1.228954000
H	3.778807000	3.415337000	0.124602000
C	1.423052000	-4.433713000	-0.326989000
H	1.913876000	-5.410457000	-0.278114000
C	0.019227000	4.825207000	-0.125976000
H	0.202429000	5.899201000	-0.027922000
C	0.045684000	-4.339963000	-0.517502000
H	-0.585844000	-5.225340000	-0.623044000

C	2.176151000	-3.271491000	-0.198050000
H	3.255957000	-3.287913000	-0.047648000
C	1.080141000	3.930870000	-0.038545000
H	2.091101000	4.301536000	0.131312000
C	4.761106000	-0.799866000	0.217545000
C	-4.713846000	-0.540988000	-1.800109000
C	4.254515000	2.431550000	0.238557000
H	-6.481961000	-0.802249000	-0.567777000
C	6.043078000	-0.028631000	0.425852000
H	-5.237737000	-0.590128000	-2.758033000
H	5.055534000	2.373791000	-0.516993000
H	6.271407000	0.611969000	-0.442964000
H	5.990452000	0.623860000	1.312971000
H	6.859306000	-0.755498000	0.558038000
Cl	-0.217913000	0.115215000	-2.885076000

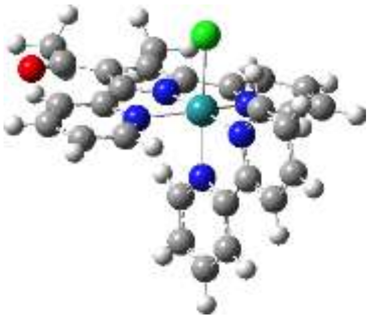

$[Ru^{III}-Cl]^+$
E = -1945.10500747
Charge = 1 Multiplicity = 2

Ru	-0.565301000	0.001394000	-0.538545000
N	-1.161726000	-0.162619000	1.423870000
N	-2.640152000	-0.266096000	-0.740858000
N	-0.413993000	2.071450000	-0.223603000
N	0.215901000	-1.922240000	-0.336717000
N	1.338166000	0.284731000	-0.234850000
C	1.885487000	1.511341000	-0.109741000
C	-3.302599000	-0.296487000	-1.902802000
H	-2.688872000	-0.181972000	-2.800556000
C	-0.314557000	-0.099944000	2.457289000
H	0.739953000	0.050551000	2.214287000
C	-2.983474000	-0.480245000	2.931845000
H	-4.050206000	-0.632917000	3.099165000
C	-2.485638000	-0.349162000	1.635080000
C	0.867694000	2.551706000	-0.104993000
C	-4.701066000	-0.569274000	0.436038000
H	-5.240418000	-0.675037000	1.378296000
C	3.280804000	1.345355000	0.051010000
C	-0.748746000	-0.220763000	3.769900000
H	-0.024320000	-0.163392000	4.585216000

C	-5.389715000	-0.601795000	-0.771837000
C	-3.316214000	-0.400375000	0.424809000
C	-1.317260000	4.270918000	-0.041858000
H	-2.200080000	4.913192000	-0.020622000
C	3.520120000	-0.068541000	0.022267000
C	-2.107207000	-0.415174000	4.009027000
H	-2.485857000	-0.516446000	5.029538000
C	1.584500000	-2.000714000	-0.222897000
C	-1.463286000	2.899464000	-0.181679000
H	-2.449535000	2.436764000	-0.272601000
C	-0.534429000	-3.028123000	-0.346952000
H	-1.614448000	-2.879141000	-0.428992000
C	2.236973000	-0.695920000	-0.159523000
O	4.845553000	-2.007266000	0.094828000
H	4.769538000	2.416710000	1.201352000
H	3.788878000	3.434002000	0.128473000
C	1.407148000	-4.405510000	-0.172180000
H	1.882423000	-5.388804000	-0.110706000
C	-0.025568000	4.787644000	0.067386000
H	0.133950000	5.863716000	0.180385000
C	0.020225000	-4.297491000	-0.270561000
H	-0.628906000	-5.175485000	-0.290490000
C	2.191013000	-3.258153000	-0.146077000
H	3.276896000	-3.292120000	-0.060185000
C	1.065774000	3.929664000	0.039235000
H	2.077230000	4.324344000	0.133431000
C	4.794676000	-0.784872000	0.156610000
C	-4.679942000	-0.463568000	-1.962467000
C	4.271923000	2.451435000	0.216763000
H	-6.474811000	-0.734781000	-0.779752000
C	6.073324000	-0.018220000	0.373606000
H	-5.180770000	-0.483325000	-2.932997000
H	5.063112000	2.410376000	-0.549789000
H	6.291247000	0.642536000	-0.482647000
H	6.019765000	0.614350000	1.275097000
H	6.893230000	-0.744008000	0.485312000
Cl	-0.125666000	0.115406000	-2.849560000

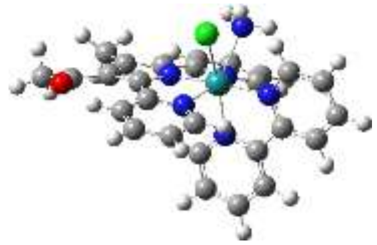

TS1-I$_a$
E = -2001.6038003
Charge = 1   Multiplicity = 2

Ru	-0.582690000	-0.051304000	-0.560236000
N	-1.072566000	0.224765000	1.384831000
N	-2.649713000	-0.390049000	-0.589045000
N	-0.277653000	2.146843000	-0.363694000
N	0.142610000	-1.871570000	0.146057000
N	1.357523000	0.228764000	-0.426733000
C	1.970925000	1.435081000	-0.452561000
C	-3.355155000	-0.766952000	-1.660953000
H	-2.774084000	-0.974014000	-2.563049000
C	-0.162402000	0.498741000	2.329153000
H	0.879701000	0.537950000	2.006808000
C	-2.811725000	0.382987000	3.012199000
H	-3.872257000	0.330667000	3.262105000
C	-2.388296000	0.155847000	1.701799000
C	1.025956000	2.541175000	-0.419765000
C	-4.663361000	-0.326283000	0.705235000
H	-5.161346000	-0.156715000	1.660672000
C	3.354702000	1.230946000	-0.260866000
C	-0.523497000	0.726387000	3.648251000
H	0.251131000	0.945546000	4.386016000
C	-5.400234000	-0.693430000	-0.415256000
C	-3.280109000	-0.185373000	0.589839000
C	-1.017732000	4.403266000	-0.165797000
H	-1.849197000	5.103355000	-0.059346000
C	3.524092000	-0.176843000	-0.057723000
C	-1.872269000	0.670826000	3.994469000
H	-2.192663000	0.848623000	5.024345000
C	1.507924000	-1.998429000	0.162144000
C	-1.259853000	3.038111000	-0.236480000
H	-2.276200000	2.632842000	-0.192967000
C	-0.643496000	-2.890236000	0.498186000
H	-1.720136000	-2.708333000	0.454971000
C	2.213381000	-0.761768000	-0.155202000
O	4.791300000	-2.139433000	0.212109000
H	4.698072000	2.623664000	0.720933000

H	4.026158000	3.194055000	-0.824448000
C	1.252625000	-4.287551000	0.881953000
H	1.696041000	-5.247067000	1.163890000
C	0.307415000	4.837253000	-0.235833000
H	0.544699000	5.903940000	-0.187337000
C	-0.132141000	-4.120620000	0.883727000
H	-0.812498000	-4.926683000	1.166707000
C	2.073847000	-3.227495000	0.519097000
H	3.160375000	-3.310513000	0.500045000
C	1.332438000	3.908831000	-0.360009000
H	2.370092000	4.239462000	-0.399024000
C	4.761560000	-0.914703000	0.223719000
C	-4.734677000	-0.925739000	-1.616916000
C	4.395666000	2.304417000	-0.293702000
H	-6.485264000	-0.807212000	-0.344827000
C	6.029695000	-0.166124000	0.548992000
H	-5.271271000	-1.233044000	-2.517486000
H	5.305627000	1.978049000	-0.818577000
H	6.462170000	0.272327000	-0.367982000
H	5.865241000	0.653313000	1.266747000
H	6.754238000	-0.884986000	0.961929000
Cl	-0.284044000	-1.800879000	-2.675546000
N	-0.888322000	1.005015000	-2.615875000
H	-0.107831000	1.620904000	-2.858217000
H	-1.751944000	1.553463000	-2.663356000
H	-0.916969000	0.237907000	-3.295743000

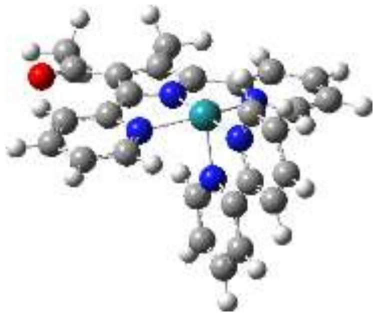

TS1-D
E = -1484.825492
Charge = 2 Multiplicity = 2

Ru	-0.582224000	0.008170000	-0.687962000
N	-1.220253000	-0.080918000	1.198527000
N	-2.638413000	-0.283171000	-0.950538000
N	-0.374289000	2.088013000	-0.415051000
N	0.189213000	-1.917642000	-0.388754000
N	1.340482000	0.275266000	-0.419862000

C	1.912814000	1.496289000	-0.319784000
C	-3.235728000	-0.353395000	-2.143253000
H	-2.583750000	-0.268101000	-3.017365000
C	-0.379593000	0.029781000	2.237110000
H	0.674131000	0.187873000	2.001595000
C	-3.049509000	-0.383520000	2.701312000
H	-4.115258000	-0.552954000	2.860372000
C	-2.547931000	-0.281493000	1.405707000
C	0.913570000	2.553024000	-0.314153000
C	-4.740065000	-0.544993000	0.147994000
H	-5.323012000	-0.614562000	1.067446000
C	3.302243000	1.310577000	-0.164291000
C	-0.827501000	-0.059768000	3.546743000
H	-0.108603000	0.033779000	4.363287000
C	-5.368666000	-0.618347000	-1.092002000
C	-3.357233000	-0.377993000	0.189248000
C	-1.248717000	4.296398000	-0.214080000
H	-2.123022000	4.949313000	-0.175528000
C	3.517823000	-0.109376000	-0.168084000
C	-2.182989000	-0.272854000	3.782470000
H	-2.566717000	-0.354103000	4.802653000
C	1.559054000	-2.016897000	-0.323705000
C	-1.415240000	2.927703000	-0.354884000
H	-2.408948000	2.477403000	-0.428518000
C	-0.582232000	-3.007971000	-0.332546000
H	-1.662298000	-2.844031000	-0.376284000
C	2.229462000	-0.718779000	-0.326054000
O	4.819020000	-2.064968000	-0.137830000
H	4.804816000	2.384763000	0.960432000
H	3.848792000	3.390978000	-0.145315000
C	1.340234000	-4.413752000	-0.186741000
H	1.800638000	-5.402969000	-0.112089000
C	0.050572000	4.797076000	-0.123322000
H	0.224308000	5.870440000	-0.006515000
C	-0.046334000	-4.283922000	-0.234407000
H	-0.711512000	-5.149299000	-0.200121000
C	2.145848000	-3.280566000	-0.229208000
H	3.233219000	-3.334430000	-0.188833000
C	1.131680000	3.926673000	-0.171238000
H	2.149579000	4.306937000	-0.089173000
C	4.786151000	-0.845975000	-0.039118000
C	-4.609516000	-0.523882000	-2.257394000
C	4.312759000	2.402328000	-0.027369000
H	-6.452842000	-0.748281000	-1.145742000

C	6.069023000	-0.102074000	0.216825000
H	-5.070457000	-0.576668000	-3.245974000
H	5.106033000	2.322825000	-0.788430000
H	6.320202000	0.555173000	-0.633070000
H	5.999402000	0.530622000	1.116998000
H	6.873953000	-0.841025000	0.348217000

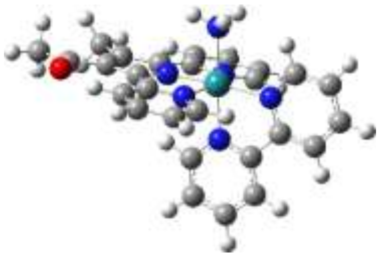

$[Ru^{III}\text{-}NH_3]^{2+}$
E = -1541.39569686
Charge = 2   Multiplicity = 2

Ru	-0.572624000	0.009215000	-0.614107000
N	-1.206122000	-0.097235000	1.347328000
N	-2.646480000	-0.270491000	-0.839654000
N	-0.369271000	2.083160000	-0.337227000
N	0.195631000	-1.915996000	-0.363257000
N	1.342986000	0.272189000	-0.290309000
C	1.913566000	1.492604000	-0.188935000
C	-3.290218000	-0.338791000	-2.011296000
H	-2.681874000	-0.251319000	-2.914125000
C	-0.377325000	-0.003822000	2.392343000
H	0.680470000	0.147358000	2.164779000
C	-3.050834000	-0.392390000	2.832629000
H	-4.119051000	-0.549279000	2.985996000
C	-2.530863000	-0.287468000	1.542672000
C	0.914869000	2.549575000	-0.208765000
C	-4.723640000	-0.549313000	0.311468000
H	-5.278264000	-0.630549000	1.247111000
C	3.304692000	1.307572000	-0.027195000
C	-0.832487000	-0.097487000	3.700849000
H	-0.121654000	-0.016727000	4.526023000
C	-5.391981000	-0.620387000	-0.906310000
C	-3.340268000	-0.374320000	0.319509000
C	-1.250831000	4.291548000	-0.175046000
H	-2.126757000	4.943309000	-0.162754000
C	3.521014000	-0.110067000	-0.032442000
C	-2.192649000	-0.296549000	3.922488000
H	-2.587149000	-0.378094000	4.938717000

C	1.561930000	-2.017355000	-0.247749000
C	-1.411960000	2.921052000	-0.306832000
H	-2.402447000	2.467598000	-0.399003000
C	-0.575435000	-3.008580000	-0.359477000
H	-1.653151000	-2.843696000	-0.440528000
C	2.230630000	-0.720310000	-0.200875000
O	4.815084000	-2.068662000	0.051617000
H	4.807495000	2.377374000	1.103962000
H	3.845528000	3.389380000	0.009215000
C	1.342481000	-4.416975000	-0.171026000
H	1.800557000	-5.407566000	-0.098706000
C	0.045492000	4.794957000	-0.059466000
H	0.215428000	5.869761000	0.049677000
C	-0.041871000	-4.286076000	-0.269547000
H	-0.706587000	-5.152405000	-0.278580000
C	2.146714000	-3.283008000	-0.157164000
H	3.231697000	-3.335541000	-0.071386000
C	1.128064000	3.925293000	-0.072775000
H	2.143154000	4.307906000	0.030890000
C	4.786353000	-0.846103000	0.106411000
C	-4.665009000	-0.514448000	-2.089678000
C	4.312693000	2.401101000	0.117720000
H	-6.476198000	-0.758486000	-0.927675000
C	6.076078000	-0.098305000	0.316855000
H	-5.149446000	-0.565075000	-3.067094000
H	5.104283000	2.331571000	-0.646195000
H	6.306884000	0.545731000	-0.548812000
H	6.029856000	0.548519000	1.208475000
H	6.883646000	-0.835678000	0.441168000
N	-0.024058000	0.086245000	-2.652543000
H	-0.303850000	-0.762432000	-3.157982000
H	-0.462028000	0.879602000	-3.135153000
H	0.990188000	0.185765000	-2.770794000

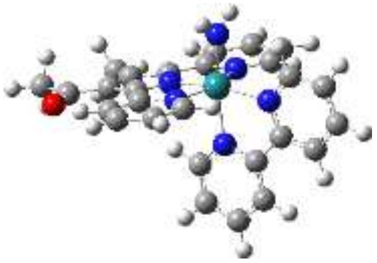

$[Ru^{III}-NH_2]^+$
E = -1540.91763016
Charge = 1 Multiplicity = 2

Ru	-0.600009000	0.008622000	-0.667390000
N	-0.986336000	-0.122477000	1.423767000
N	-2.692808000	-0.303059000	-0.570722000
N	-0.469871000	2.114916000	-0.564103000
N	0.172111000	-1.947424000	-0.686840000
N	1.280232000	0.287047000	-0.394539000
C	1.813973000	1.516171000	-0.211737000
C	-3.488972000	-0.374655000	-1.645397000
H	-2.984981000	-0.268318000	-2.609330000
C	-0.049356000	-0.016465000	2.372374000
H	0.974736000	0.151848000	2.030690000
C	-2.640851000	-0.439285000	3.121731000
H	-3.682119000	-0.609625000	3.397913000
C	-2.274046000	-0.329676000	1.778632000
C	0.808222000	2.573698000	-0.346146000
C	-4.601697000	-0.624780000	0.837195000
H	-5.024229000	-0.719254000	1.838377000
C	3.183774000	1.343057000	0.067240000
C	-0.344706000	-0.112933000	3.726118000
H	0.457122000	-0.019136000	4.462049000
C	-5.426994000	-0.698844000	-0.278660000
C	-3.229984000	-0.425528000	0.664078000
C	-1.298808000	4.356994000	-0.650774000
H	-2.155646000	5.022944000	-0.774411000
C	3.428237000	-0.079070000	0.023079000
C	-1.666508000	-0.330026000	4.107075000
H	-1.940090000	-0.413884000	5.162257000
C	1.528313000	-2.013795000	-0.454700000
C	-1.475733000	2.979096000	-0.709233000
H	-2.463376000	2.540765000	-0.879476000
C	-0.525665000	-3.065697000	-0.886322000
H	-1.597004000	-2.936654000	-1.064852000
C	2.167000000	-0.702145000	-0.266614000
O	4.752324000	-2.013373000	0.215205000
H	4.717850000	2.234604000	1.302690000
H	3.638661000	3.396033000	0.519922000
C	1.423084000	-4.419684000	-0.625944000
H	1.921048000	-5.393354000	-0.598730000
C	-0.011763000	4.844719000	-0.439120000
H	0.177153000	5.921093000	-0.394100000
C	0.055774000	-4.329775000	-0.868126000
H	-0.561349000	-5.214805000	-1.038925000
C	2.161740000	-3.258059000	-0.416591000
H	3.233291000	-3.276315000	-0.218331000

C	1.043704000	3.950830000	-0.289941000
H	2.057069000	4.322800000	-0.139964000
C	4.699215000	-0.789072000	0.212973000
C	-4.859778000	-0.571468000	-1.544952000
C	4.155145000	2.436492000	0.377359000
H	-6.502416000	-0.854402000	-0.157898000
C	5.979841000	-0.015192000	0.397223000
H	-5.466622000	-0.622274000	-2.452031000
H	4.894861000	2.578625000	-0.430435000
H	6.130642000	0.728771000	-0.401854000
H	5.979117000	0.525713000	1.359118000
H	6.815081000	-0.732171000	0.395258000
N	-0.540403000	0.073120000	-2.588537000
H	-0.257776000	-0.714786000	-3.174275000
H	-0.638669000	0.935093000	-3.128229000

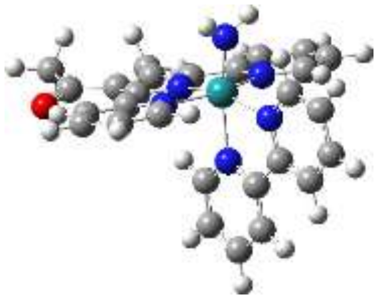

$[Ru^{IV}\text{-}NH_2]^{2+}$
E = -1540.7411905
Charge = 2 Multiplicity = 1

Ru	-0.570145000	0.029774000	-0.734826000
N	-0.921641000	-0.093487000	1.411377000
N	-2.672422000	-0.305119000	-0.514995000
N	-0.478486000	2.133463000	-0.605777000
N	0.152987000	-1.942952000	-0.732154000
N	1.234656000	0.294329000	-0.359680000
C	1.780929000	1.532250000	-0.177427000
C	-3.501026000	-0.385237000	-1.568456000
H	-3.054933000	-0.265250000	-2.556061000
C	0.041229000	0.042133000	2.331060000
H	1.052743000	0.239182000	1.974442000
C	-2.511991000	-0.443878000	3.163601000
H	-3.540097000	-0.637349000	3.470531000
C	-2.187425000	-0.329269000	1.812130000
C	0.784033000	2.594744000	-0.329607000
C	-4.530443000	-0.673914000	0.944330000
H	-4.917381000	-0.782997000	1.957809000

C	3.135500000	1.341592000	0.085079000
C	-0.210282000	-0.057375000	3.693317000
H	0.613976000	0.061363000	4.399850000
C	-5.387981000	-0.758812000	-0.145216000
C	-3.171128000	-0.444191000	0.730690000
C	-1.321068000	4.362659000	-0.682125000
H	-2.176646000	5.025623000	-0.826690000
C	3.368538000	-0.101165000	0.040197000
C	-1.510741000	-0.307708000	4.117816000
H	-1.748633000	-0.395185000	5.181154000
C	1.493009000	-2.025778000	-0.437780000
C	-1.489739000	2.985734000	-0.774822000
H	-2.465546000	2.542544000	-0.992211000
C	-0.558075000	-3.043453000	-0.964446000
H	-1.617714000	-2.897712000	-1.192474000
C	2.129554000	-0.717476000	-0.225624000
O	4.663388000	-2.027183000	0.389498000
H	4.751163000	2.139790000	1.258313000
H	3.642819000	3.361873000	0.609589000
C	1.358712000	-4.424427000	-0.611489000
H	1.839578000	-5.405151000	-0.558633000
C	-0.048222000	4.854568000	-0.408587000
H	0.128392000	5.931040000	-0.332771000
C	0.008088000	-4.315134000	-0.918976000
H	-0.612287000	-5.191637000	-1.117822000
C	2.107113000	-3.274097000	-0.363975000
H	3.164623000	-3.316721000	-0.106457000
C	1.010617000	3.967598000	-0.233537000
H	2.013644000	4.342295000	-0.030570000
C	4.653346000	-0.820999000	0.207860000
C	-4.862484000	-0.610485000	-1.425080000
C	4.135141000	2.405503000	0.385236000
H	-6.455443000	-0.938158000	0.007230000
C	5.951893000	-0.069583000	0.129872000
H	-5.491598000	-0.666336000	-2.316173000
H	4.821230000	2.572298000	-0.463939000
H	5.973371000	0.633380000	-0.718325000
H	6.113785000	0.513486000	1.053403000
H	6.767771000	-0.801920000	0.032691000
N	-0.673205000	0.059716000	-2.559105000
H	-0.607437000	-0.784065000	-3.142022000
H	-0.911890000	0.902571000	-3.096393000

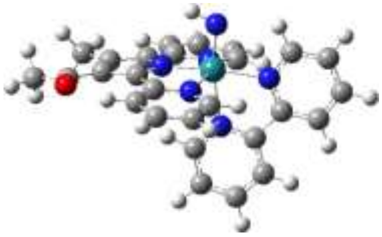

$[Ru^{IV}\text{-}NH]^{+}$
E = -1540.2621121
Charge = 1   Multiplicity = 1

Ru	-0.546887000	0.016556000	-0.758613000
N	-1.142460000	-0.137528000	1.356467000
N	-2.663869000	-0.265341000	-0.820819000
N	-0.400008000	2.080197000	-0.368706000
N	0.201516000	-1.909803000	-0.456489000
N	1.355666000	0.293049000	-0.534016000
C	1.903337000	1.514136000	-0.340771000
C	-3.356996000	-0.306077000	-1.961552000
H	-2.757432000	-0.203708000	-2.871244000
C	-0.295088000	-0.065651000	2.383802000
H	0.759563000	0.087490000	2.135070000
C	-2.957441000	-0.454648000	2.868843000
H	-4.022145000	-0.611889000	3.044781000
C	-2.458415000	-0.326708000	1.569798000
C	0.886773000	2.553172000	-0.274357000
C	-4.692599000	-0.550912000	0.408860000
H	-5.207938000	-0.645183000	1.365609000
C	3.283974000	1.335029000	-0.090713000
C	-0.719414000	-0.179646000	3.702099000
H	0.004371000	-0.114574000	4.517640000
C	-5.415407000	-0.593140000	-0.778232000
C	-3.306054000	-0.385779000	0.361726000
C	-1.291117000	4.272869000	-0.074861000
H	-2.170713000	4.915847000	-0.002544000
C	3.514513000	-0.079156000	-0.118220000
C	-2.077158000	-0.379517000	3.943074000
H	-2.453469000	-0.477668000	4.965066000
C	1.572277000	-1.993313000	-0.369116000
C	-1.444475000	2.909247000	-0.270305000
H	-2.433393000	2.451765000	-0.357329000
C	-0.554955000	-3.009589000	-0.387747000
H	-1.634432000	-2.857814000	-0.468266000
C	2.240870000	-0.695734000	-0.390540000
O	4.842870000	-2.016908000	-0.073745000
H	4.672438000	2.419304000	1.170535000
H	3.816827000	3.418236000	-0.021839000

C	1.378815000	-4.388707000	-0.150531000
H	1.848142000	-5.369840000	-0.032912000
C	0.005262000	4.782086000	0.018634000
H	0.171264000	5.852501000	0.170150000
C	-0.009347000	-4.274946000	-0.230554000
H	-0.665396000	-5.146683000	-0.181619000
C	2.170604000	-3.249530000	-0.221282000
H	3.258256000	-3.288147000	-0.161940000
C	1.092544000	3.924609000	-0.079561000
H	2.107489000	4.314293000	-0.004216000
C	4.773567000	-0.804113000	0.087541000
C	-4.736317000	-0.469184000	-1.987970000
C	4.272438000	2.431984000	0.141557000
H	-6.500866000	-0.722197000	-0.754828000
C	6.012996000	-0.058942000	0.511533000
H	-5.262358000	-0.497194000	-2.945119000
H	5.133219000	2.358311000	-0.543036000
H	6.353233000	0.625967000	-0.283801000
H	5.837865000	0.545122000	1.416935000
H	6.806155000	-0.796338000	0.708472000
N	-0.441183000	-0.002933000	-2.548340000
H	0.532622000	-0.001328000	-2.920248000

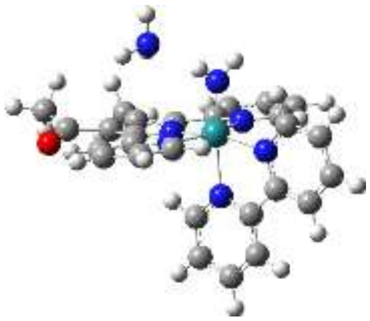

TS2
E = -1597.26605053
Charge = 2 Multiplicity = 1

Ru	-0.595959000	0.016624000	-0.599143000
N	-1.195791000	-0.161197000	1.484405000
N	-2.699209000	-0.321696000	-0.652069000
N	-0.552890000	2.125164000	-0.458065000
N	0.177785000	-1.935492000	-0.541622000
N	1.167902000	0.319246000	-0.034156000
C	1.685937000	1.568167000	0.124945000
C	-3.386606000	-0.386853000	-1.802729000
H	-2.810937000	-0.278390000	-2.723001000
C	-0.347398000	-0.079049000	2.514666000

H	0.703007000	0.098253000	2.277934000
C	-2.994087000	-0.521516000	3.018624000
H	-4.055347000	-0.700658000	3.193238000
C	-2.506112000	-0.377931000	1.719896000
C	0.680604000	2.609867000	-0.100134000
C	-4.732589000	-0.650315000	0.560339000
H	-5.248882000	-0.751477000	1.515600000
C	3.047161000	1.412976000	0.398451000
C	-0.763538000	-0.209783000	3.833598000
H	-0.029539000	-0.134253000	4.638875000
C	-5.446160000	-0.715257000	-0.630105000
C	-3.351623000	-0.454568000	0.521941000
C	-1.424485000	4.340177000	-0.614148000
H	-2.278655000	4.987150000	-0.824649000
C	3.311460000	-0.019388000	0.377061000
C	-2.112033000	-0.436302000	4.089693000
H	-2.478551000	-0.546850000	5.113635000
C	1.495308000	-1.986338000	-0.154339000
C	-1.563043000	2.959831000	-0.704725000
H	-2.513861000	2.498879000	-0.986478000
C	-0.484177000	-3.053608000	-0.830778000
H	-1.528601000	-2.933860000	-1.131155000
C	2.082946000	-0.665349000	0.108607000
O	4.649982000	-1.912056000	0.752479000
H	4.642497000	2.272019000	1.561020000
H	3.508206000	3.455104000	0.888677000
C	1.441022000	-4.386296000	-0.359910000
H	1.944229000	-5.354267000	-0.283491000
C	-0.181784000	4.855624000	-0.256861000
H	-0.028554000	5.935458000	-0.177435000
C	0.111382000	-4.310092000	-0.757553000
H	-0.469706000	-5.200662000	-1.006518000
C	2.137537000	-3.218997000	-0.050797000
H	3.176387000	-3.233608000	0.276860000
C	0.876047000	3.987980000	-0.000294000
H	1.854909000	4.381578000	0.272861000
C	4.608393000	-0.708548000	0.551253000
C	-4.759932000	-0.583224000	-1.833916000
C	4.021973000	2.506092000	0.681949000
H	-6.528228000	-0.869246000	-0.614521000
C	5.890710000	0.069973000	0.452999000
H	-5.273177000	-0.629595000	-2.796877000
H	4.706526000	2.675305000	-0.167987000
H	5.892949000	0.756758000	-0.408671000

H	6.044212000	0.674060000	1.364229000
H	6.722152000	-0.646003000	0.365577000
N	-0.363674000	0.077273000	-2.405068000
N	2.267972000	-0.222176000	-3.088051000
H	2.629981000	0.560566000	-2.537847000
H	2.755112000	-1.060995000	-2.762711000
H	2.545287000	-0.065579000	-4.060094000
H	-0.515613000	0.922629000	-2.970827000
H	0.020396000	-0.698798000	-2.957791000

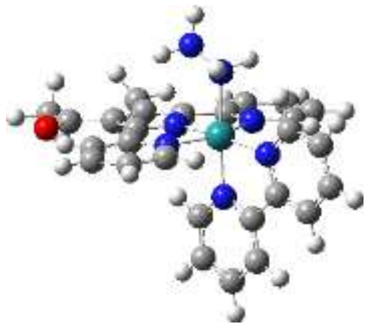

$[Ru^{II}\text{-}NH_2\text{-}NH_3]^{2+}$
E = -1597.2891391
Charge = 2   Multiplicity = 1

Ru	-0.631966000	0.009316000	-0.530422000
N	-1.090677000	-0.129284000	1.447751000
N	-2.705787000	-0.280906000	-0.611344000
N	-0.467471000	2.110456000	-0.392490000
N	0.135315000	-1.954688000	-0.512072000
N	1.265807000	0.268715000	-0.245345000
C	1.816189000	1.491928000	-0.063009000
C	-3.470174000	-0.336363000	-1.712061000
H	-2.962879000	-0.221429000	-2.672724000
C	-0.191850000	-0.036697000	2.438838000
H	0.843741000	0.136484000	2.140203000
C	-2.811595000	-0.472575000	3.078142000
H	-3.863147000	-0.648504000	3.308194000
C	-2.395499000	-0.341510000	1.751543000
C	0.811285000	2.555897000	-0.139710000
C	-4.681270000	-0.609444000	0.716442000
H	-5.142012000	-0.712415000	1.700026000
C	3.194716000	1.308114000	0.188441000
C	-0.542937000	-0.154267000	3.776292000
H	0.229752000	-0.071025000	4.544075000
C	-5.464524000	-0.666727000	-0.429446000
C	-3.302496000	-0.417223000	0.599834000
C	-1.297079000	4.356232000	-0.320602000

H	-2.154511000	5.028451000	-0.398038000
C	3.423140000	-0.111572000	0.158236000
C	-1.878520000	-0.379082000	4.103378000
H	-2.192846000	-0.480431000	5.145477000
C	1.491671000	-2.031656000	-0.271541000
C	-1.472548000	2.986400000	-0.476994000
H	-2.460891000	2.561764000	-0.675394000
C	-0.574871000	-3.074447000	-0.667776000
H	-1.644335000	-2.940414000	-0.854421000
C	2.147201000	-0.724743000	-0.119841000
O	4.744564000	-2.054138000	0.266850000
H	4.599275000	2.371588000	1.450236000
H	3.730747000	3.389750000	0.287875000
C	1.356312000	-4.442322000	-0.354225000
H	1.841952000	-5.420515000	-0.289996000
C	-0.012467000	4.827910000	-0.060879000
H	0.173745000	5.897198000	0.074293000
C	-0.011098000	-4.343865000	-0.596919000
H	-0.640510000	-5.226781000	-0.729775000
C	2.109335000	-3.283022000	-0.193552000
H	3.183021000	-3.305967000	-0.006962000
C	1.041504000	3.925254000	0.030006000
H	2.048885000	4.284055000	0.240262000
C	4.682522000	-0.833145000	0.363942000
C	-4.844390000	-0.527373000	-1.669684000
C	4.187976000	2.400478000	0.425976000
H	-6.544561000	-0.817444000	-0.353408000
C	5.942598000	-0.078505000	0.706514000
H	-5.412500000	-0.563261000	-2.602170000
H	5.042488000	2.341572000	-0.267767000
H	6.242989000	0.590531000	-0.117810000
H	5.815264000	0.542809000	1.608158000
H	6.743663000	-0.813266000	0.879788000
N	-0.349231000	0.133623000	-2.662120000
N	0.989281000	0.279527000	-3.146239000
H	1.428351000	1.110348000	-2.713554000
H	1.553171000	-0.544739000	-2.878752000
H	1.030323000	0.384113000	-4.175723000
H	-0.872331000	0.937587000	-3.031785000
H	-0.726289000	-0.705009000	-3.121589000

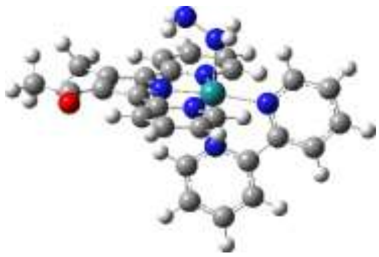

[Ru^{II}-NH_2-NH_2]$^+$
E = -1596.8386926
Charge = 1 Multiplicity = 1

Ru	-0.610407000	0.004213000	-0.495994000
N	-1.171062000	-0.117025000	1.472722000
N	-2.674296000	-0.281614000	-0.668073000
N	-0.437163000	2.102734000	-0.397590000
N	0.151197000	-1.953952000	-0.476712000
N	1.273350000	0.258785000	-0.149899000
C	1.833810000	1.481882000	-0.013414000
C	-3.379345000	-0.354950000	-1.807699000
H	-2.817764000	-0.260336000	-2.739753000
C	-0.324262000	-0.024578000	2.508893000
H	0.727445000	0.130569000	2.258140000
C	-2.979558000	-0.418691000	3.015793000
H	-4.043986000	-0.578034000	3.193542000
C	-2.492261000	-0.310619000	1.710571000
C	0.839672000	2.548605000	-0.134631000
C	-4.721212000	-0.581401000	0.555881000
H	-5.235177000	-0.667709000	1.514392000
C	3.220393000	1.300651000	0.192388000
C	-0.745355000	-0.120474000	3.827602000
H	-0.013549000	-0.037917000	4.634703000
C	-5.442013000	-0.655237000	-0.629157000
C	-3.336607000	-0.396300000	0.512384000
C	-1.250271000	4.356932000	-0.425297000
H	-2.100396000	5.031875000	-0.547523000
C	3.446296000	-0.119531000	0.175647000
C	-2.100252000	-0.322808000	4.087093000
H	-2.469889000	-0.405650000	5.112611000
C	1.506361000	-2.035239000	-0.228771000
C	-1.432503000	2.983463000	-0.535587000
H	-2.419659000	2.559466000	-0.741707000
C	-0.555046000	-3.072316000	-0.662867000
H	-1.623383000	-2.935880000	-0.854762000
C	2.157975000	-0.732522000	-0.052307000
O	4.759689000	-2.065749000	0.326287000
H	4.681344000	2.372774000	1.383136000

H	3.756889000	3.383595000	0.259223000
C	1.376137000	-4.444691000	-0.367599000
H	1.862785000	-5.423558000	-0.322661000
C	0.032030000	4.828952000	-0.153170000
H	0.223746000	5.901071000	-0.051951000
C	0.009812000	-4.342163000	-0.618343000
H	-0.617246000	-5.222525000	-0.777476000
C	2.124915000	-3.288364000	-0.173167000
H	3.196844000	-3.312674000	0.023973000
C	1.075906000	3.922222000	-0.006482000
H	2.081100000	4.281152000	0.214089000
C	4.707035000	-0.839665000	0.357671000
C	-4.754237000	-0.540452000	-1.836509000
C	4.221996000	2.395572000	0.379232000
H	-6.525232000	-0.800975000	-0.609340000
C	5.989569000	-0.079250000	0.592360000
H	-5.271025000	-0.592039000	-2.797775000
H	5.043446000	2.336231000	-0.353649000
H	6.238809000	0.562420000	-0.269824000
H	5.923464000	0.570729000	1.480405000
H	6.797808000	-0.812184000	0.738589000
N	-0.258181000	0.097329000	-2.591625000
N	1.058469000	0.278328000	-3.141863000
H	1.432813000	1.122307000	-2.700002000
H	1.627759000	-0.483311000	-2.763295000
H	-0.826492000	0.850903000	-2.991659000
H	-0.620198000	-0.755918000	-3.029767000

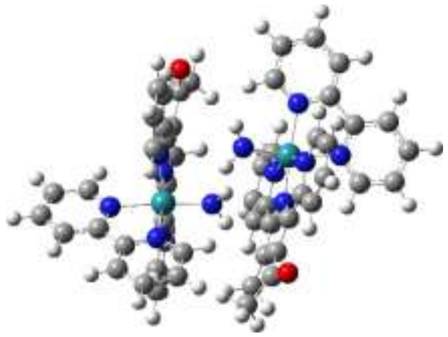

Reactant complex
E = -3081.49966316
Charge = 4 Multiplicity = 1

Ru	2.860193000	0.163962000	-0.299829000
N	4.741498000	1.068745000	-0.000493000
N	4.200470000	-1.334973000	-0.944640000
N	2.821232000	-0.249228000	1.748180000

N	2.573425000	1.475025000	-1.905669000
N	1.821629000	1.632672000	0.447906000
C	1.399511000	1.638392000	1.733524000
C	3.823555000	-2.538508000	-1.396910000
H	2.749359000	-2.733740000	-1.431401000
C	4.901538000	2.302462000	0.487455000
H	3.992139000	2.854201000	0.736896000
C	7.107440000	0.819523000	-0.170213000
H	7.974632000	0.213026000	-0.434336000
C	5.816005000	0.319511000	-0.334317000
C	2.004143000	0.561299000	2.499469000
C	6.489936000	-1.937573000	-1.251305000
H	7.547701000	-1.680693000	-1.187613000
C	0.486687000	2.703788000	1.875500000
C	6.160328000	2.859729000	0.671126000
H	6.248771000	3.871718000	1.071769000
C	6.100850000	-3.187268000	-1.722462000
C	5.515750000	-1.020138000	-0.861312000
C	3.417522000	-1.525556000	3.667780000
H	3.989610000	-2.353510000	4.090695000
C	0.362563000	3.301493000	0.573072000
C	7.279842000	2.103025000	0.337695000
H	8.286984000	2.506711000	0.470684000
C	1.708756000	2.519766000	-1.678039000
C	3.516630000	-1.243154000	2.314867000
H	4.156418000	-1.831576000	1.653263000
C	3.153927000	1.307278000	-3.097106000
H	3.840043000	0.461682000	-3.191829000
C	1.251143000	2.591799000	-0.293788000
O	-0.581412000	4.734262000	-1.027694000
H	-0.041307000	4.200870000	3.328396000
H	0.244179000	2.590461000	4.008758000
C	1.987864000	3.199549000	-3.971672000
H	1.749937000	3.882615000	-4.791829000
C	2.567640000	-0.740946000	4.448409000
H	2.457665000	-0.939051000	5.518017000
C	2.882363000	2.149406000	-4.166756000
H	3.365474000	1.972001000	-5.129796000
C	1.401612000	3.390403000	-2.724255000
H	0.706764000	4.204942000	-2.524112000
C	1.859813000	0.302299000	3.865568000
H	1.204473000	0.926812000	4.471324000
C	-0.577932000	4.347182000	0.132765000
C	4.744993000	-3.496100000	-1.795009000

C	-0.174826000	3.122753000	3.144780000
H	6.856728000	-3.914716000	-2.029458000
C	-1.585223000	4.906509000	1.097354000
H	4.393387000	-4.465449000	-2.154686000
H	-1.259273000	2.925122000	3.128772000
H	-2.191623000	4.105660000	1.552580000
H	-1.087283000	5.451691000	1.916991000
H	-2.241527000	5.599545000	0.549779000
N	1.286555000	-0.886879000	-0.663709000
H	0.966396000	-1.077612000	-1.619346000
H	0.934515000	-1.579722000	0.004589000
Ru	-2.841967000	-0.186726000	-0.201911000
N	-4.767859000	-1.041485000	-0.076943000
N	-4.112090000	1.387710000	-0.877898000
N	-2.845871000	0.238399000	1.864248000
N	-2.522706000	-1.388672000	-1.901576000
N	-1.871898000	-1.642247000	0.511601000
C	-1.470937000	-1.694680000	1.806242000
C	-3.696330000	2.617372000	-1.200520000
H	-2.624563000	2.815760000	-1.145386000
C	-4.989766000	-2.295941000	0.331738000
H	-4.119999000	-2.865695000	0.666872000
C	-7.092313000	-0.773097000	-0.533743000
H	-7.916193000	-0.153118000	-0.888827000
C	-5.792525000	-0.269490000	-0.503014000
C	-2.074749000	-0.626531000	2.600631000
C	-6.369704000	2.061294000	-1.285705000
H	-7.433736000	1.822056000	-1.298420000
C	-0.589396000	-2.777451000	1.924062000
C	-6.261128000	-2.853015000	0.331782000
H	-6.399900000	-3.880405000	0.674842000
C	-5.935785000	3.336713000	-1.630712000
C	-5.433155000	1.097740000	-0.910650000
C	-3.436946000	1.452471000	3.827551000
H	-3.991822000	2.279875000	4.274156000
C	-0.475799000	-3.361725000	0.592779000
C	-7.328208000	-2.077774000	-0.113314000
H	-8.343037000	-2.483507000	-0.134592000
C	-1.750805000	-2.498269000	-1.653093000
C	-3.507606000	1.234230000	2.458250000
H	-4.105777000	1.876554000	1.807071000
C	-2.999515000	-1.153880000	-3.121969000
H	-3.607325000	-0.253037000	-3.241105000
C	-1.333256000	-2.625753000	-0.254363000

O	0.288294000	-4.985259000	-0.923980000
H	0.012750000	-4.351953000	3.252265000
H	-0.398385000	-2.824370000	4.062896000
C	-1.949887000	-3.127682000	-3.968589000
H	-1.722214000	-3.818146000	-4.785363000
C	-2.638096000	0.605131000	4.592527000
H	-2.548076000	0.751343000	5.672321000
C	-2.733237000	-2.000927000	-4.193936000
H	-3.140221000	-1.768400000	-5.180265000
C	-1.459626000	-3.383788000	-2.689756000
H	-0.857554000	-4.263398000	-2.463257000
C	-1.951850000	-0.437187000	3.977734000
H	-1.330402000	-1.110805000	4.567418000
C	0.459819000	-4.416171000	0.141238000
C	-4.574326000	3.621515000	-1.586172000
C	0.072124000	-3.255127000	3.168406000
H	-6.659481000	4.101229000	-1.924763000
C	1.669141000	-4.732409000	0.976248000
H	-4.180918000	4.608487000	-1.839391000
H	1.141296000	-2.982506000	3.190611000
H	2.160792000	-3.820193000	1.353961000
H	1.382435000	-5.344150000	1.849519000
H	2.372971000	-5.313670000	0.361143000
N	-1.226257000	0.790132000	-0.550823000
H	-1.112936000	1.422249000	-1.347914000
H	-0.317184000	0.495099000	-0.172450000

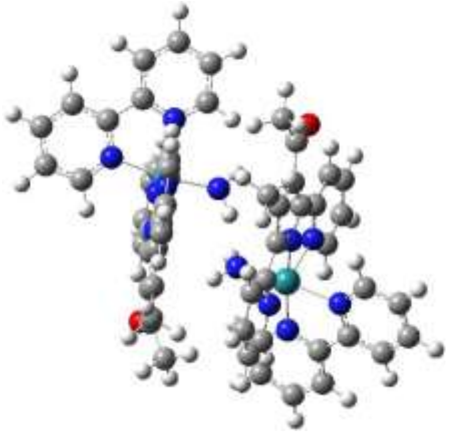

TS-coupling
E = -3081.475876
Charge = 4 Multiplicity = 1

Ru	2.860193000	0.163962000	-0.299829000
N	4.741498000	1.068745000	-0.000493000
N	4.200470000	-1.334973000	-0.944640000

N	2.821232000	-0.249228000	1.748180000
N	2.573425000	1.475025000	-1.905669000
N	1.821629000	1.632672000	0.447906000
C	1.399511000	1.638392000	1.733524000
C	3.823555000	-2.538508000	-1.396910000
H	2.749359000	-2.733740000	-1.431401000
C	4.901538000	2.302462000	0.487455000
H	3.992139000	2.854201000	0.736896000
C	7.107440000	0.819523000	-0.170213000
H	7.974632000	0.213026000	-0.434336000
C	5.816005000	0.319511000	-0.334317000
C	2.004143000	0.561299000	2.499469000
C	6.489936000	-1.937573000	-1.251305000
H	7.547701000	-1.680693000	-1.187613000
C	0.486687000	2.703788000	1.875500000
C	6.160328000	2.859729000	0.671126000
H	6.248771000	3.871718000	1.071769000
C	6.100850000	-3.187268000	-1.722462000
C	5.515750000	-1.020138000	-0.861312000
C	3.417522000	-1.525556000	3.667780000
H	3.989610000	-2.353510000	4.090695000
C	0.362563000	3.301493000	0.573072000
C	7.279842000	2.103025000	0.337695000
H	8.286984000	2.506711000	0.470684000
C	1.708756000	2.519766000	-1.678039000
C	3.516630000	-1.243154000	2.314867000
H	4.156418000	-1.831576000	1.653263000
C	3.153927000	1.307278000	-3.097106000
H	3.840043000	0.461682000	-3.191829000
C	1.251143000	2.591799000	-0.293788000
O	-0.581412000	4.734262000	-1.027694000
H	-0.041307000	4.200870000	3.328396000
H	0.244179000	2.590461000	4.008758000
C	1.987864000	3.199549000	-3.971672000
H	1.749937000	3.882615000	-4.791829000
C	2.567640000	-0.740946000	4.448409000
H	2.457665000	-0.939051000	5.518017000
C	2.882363000	2.149406000	-4.166756000
H	3.365474000	1.972001000	-5.129796000
C	1.401612000	3.390403000	-2.724255000
H	0.706764000	4.204942000	-2.524112000
C	1.859813000	0.302299000	3.865568000
H	1.204473000	0.926812000	4.471324000
C	-0.577932000	4.347182000	0.132765000

C	4.744993000	-3.496100000	-1.795009000
C	-0.174826000	3.122753000	3.144780000
H	6.856728000	-3.914716000	-2.029458000
C	-1.585223000	4.906509000	1.097354000
H	4.393387000	-4.465449000	-2.154686000
H	-1.259273000	2.925122000	3.128772000
H	-2.191623000	4.105660000	1.552580000
H	-1.087283000	5.451691000	1.916991000
H	-2.241527000	5.599545000	0.549779000
N	1.286555000	-0.886879000	-0.663709000
H	0.966396000	-1.077612000	-1.619346000
H	0.934515000	-1.579722000	0.004589000
Ru	-2.841967000	-0.186726000	-0.201911000
N	-4.767859000	-1.041485000	-0.076943000
N	-4.112090000	1.387710000	-0.877898000
N	-2.845871000	0.238399000	1.864248000
N	-2.522706000	-1.388672000	-1.901576000
N	-1.871898000	-1.642247000	0.511601000
C	-1.470937000	-1.694680000	1.806242000
C	-3.696330000	2.617372000	-1.200520000
H	-2.624563000	2.815760000	-1.145386000
C	-4.989766000	-2.295941000	0.331738000
H	-4.119999000	-2.865695000	0.666872000
C	-7.092313000	-0.773097000	-0.533743000
H	-7.916193000	-0.153118000	-0.888827000
C	-5.792525000	-0.269490000	-0.503014000
C	-2.074749000	-0.626531000	2.600631000
C	-6.369704000	2.061294000	-1.285705000
H	-7.433736000	1.822056000	-1.298420000
C	-0.589396000	-2.777451000	1.924062000
C	-6.261128000	-2.853015000	0.331782000
H	-6.399900000	-3.880405000	0.674842000
C	-5.935785000	3.336713000	-1.630712000
C	-5.433155000	1.097740000	-0.910650000
C	-3.436946000	1.452471000	3.827551000
H	-3.991822000	2.279875000	4.274156000
C	-0.475799000	-3.361725000	0.592779000
C	-7.328208000	-2.077774000	-0.113314000
H	-8.343037000	-2.483507000	-0.134592000
C	-1.750805000	-2.498269000	-1.653093000
C	-3.507606000	1.234230000	2.458250000
H	-4.105777000	1.876554000	1.807071000
C	-2.999515000	-1.153880000	-3.121969000
H	-3.607325000	-0.253037000	-3.241105000

C	-1.333256000	-2.625753000	-0.254363000
O	0.288294000	-4.985259000	-0.923980000
H	0.012750000	-4.351953000	3.252265000
H	-0.398385000	-2.824370000	4.062896000
C	-1.949887000	-3.127682000	-3.968589000
H	-1.722214000	-3.818146000	-4.785363000
C	-2.638096000	0.605131000	4.592527000
H	-2.548076000	0.751343000	5.672321000
C	-2.733237000	-2.000927000	-4.193936000
H	-3.140221000	-1.768400000	-5.180265000
C	-1.459626000	-3.383788000	-2.689756000
H	-0.857554000	-4.263398000	-2.463257000
C	-1.951850000	-0.437187000	3.977734000
H	-1.330402000	-1.110805000	4.567418000
C	0.459819000	-4.416171000	0.141238000
C	-4.574326000	3.621515000	-1.586172000
C	0.072124000	-3.255127000	3.168406000
H	-6.659481000	4.101229000	-1.924763000
C	1.669141000	-4.732409000	0.976248000
H	-4.180918000	4.608487000	-1.839391000
H	1.141296000	-2.982506000	3.190611000
H	2.160792000	-3.820193000	1.353961000
H	1.382435000	-5.344150000	1.849519000
H	2.372971000	-5.313670000	0.361143000
N	-1.226257000	0.790132000	-0.550823000
H	-1.112936000	1.422249000	-1.347914000
H	-0.317184000	0.495099000	-0.172450000

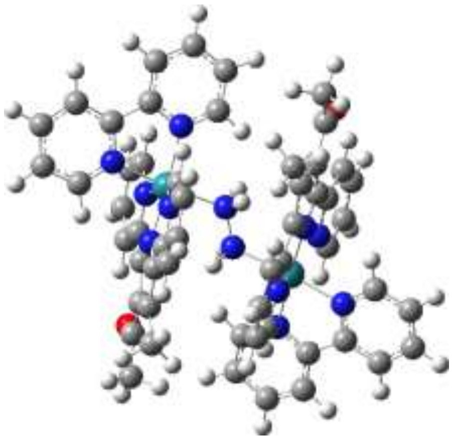

$[Ru^{II}\text{-}NH_2\text{-}NH_2\text{-}Ru^{II}]^{4+}$
E = -3081.54649445
Charge = 4 Multiplicity = 1

Ru	2.558072000	0.219895000	-0.192989000
N	4.530199000	0.702479000	0.112058000

N	3.523096000	-1.601009000	-0.622413000
N	2.309985000	0.090345000	1.893289000
N	2.640626000	1.388646000	-1.926001000
N	1.754896000	1.941367000	0.311873000
C	1.241028000	2.168403000	1.544020000
C	2.905043000	-2.733333000	-0.980803000
H	1.815868000	-2.704473000	-1.051084000
C	4.933605000	1.921772000	0.485262000
H	4.152551000	2.670574000	0.632793000
C	6.797972000	-0.041327000	0.075369000
H	7.524931000	-0.835849000	-0.096727000
C	5.435393000	-0.282378000	-0.095105000
C	1.622782000	1.122096000	2.482003000
C	5.630132000	-2.719848000	-0.748272000
H	6.715411000	-2.697658000	-0.643777000
C	0.492119000	3.363968000	1.485331000
C	6.275775000	2.223675000	0.673540000
H	6.562410000	3.232359000	0.978375000
C	4.987162000	-3.894306000	-1.126746000
C	4.871861000	-1.576052000	-0.501234000
C	2.537227000	-1.025696000	3.987164000
H	2.909974000	-1.888115000	4.543200000
C	0.601010000	3.836386000	0.133409000
C	7.222262000	1.224793000	0.463996000
H	8.287895000	1.426857000	0.600366000
C	1.971248000	2.588480000	-1.887558000
C	2.761216000	-0.937623000	2.621460000
H	3.312042000	-1.712308000	2.082084000
C	3.268995000	0.987515000	-3.035218000
H	3.791623000	0.028704000	-2.978556000
C	1.418084000	2.889572000	-0.568401000
O	0.090691000	5.242671000	-1.675339000
H	0.158953000	4.941361000	2.925179000
H	-0.246951000	3.311959000	3.502824000
C	2.555479000	2.948560000	-4.196568000
H	2.517215000	3.567787000	-5.097215000
C	1.841220000	0.009455000	4.609525000
H	1.652169000	-0.017477000	5.686134000
C	3.249861000	1.740582000	-4.201274000
H	3.770137000	1.375882000	-5.089343000
C	1.914280000	3.376277000	-3.038145000
H	1.362045000	4.314059000	-2.988205000
C	1.389760000	1.088177000	3.858732000
H	0.861708000	1.909177000	4.341643000

C	-0.025356000	5.020907000	-0.477673000
C	3.599157000	-3.904867000	-1.246098000
C	-0.264953000	3.968350000	2.622346000
H	5.570850000	-4.797103000	-1.324991000
C	-0.834798000	5.966611000	0.366108000
H	3.049836000	-4.802065000	-1.539701000
H	-1.322656000	4.138286000	2.365869000
H	-1.746678000	5.472406000	0.742461000
H	-0.265855000	6.324130000	1.239926000
H	-1.126413000	6.823161000	-0.260267000
N	0.551895000	-0.454925000	-0.524513000
H	0.513184000	-0.957273000	-1.420095000
H	0.369044000	-1.154620000	0.206492000
Ru	-2.558108000	-0.219956000	-0.193099000
N	-4.530241000	-0.702519000	0.111890000
N	-3.523083000	1.600972000	-0.622521000
N	-2.310191000	-0.090305000	1.893236000
N	-2.640518000	-1.388755000	-1.926038000
N	-1.754903000	-1.941361000	0.311933000
C	-1.241154000	-2.168364000	1.544136000
C	-2.905004000	2.733282000	-0.980909000
H	-1.815833000	2.704384000	-1.051249000
C	-4.933684000	-1.921817000	0.485031000
H	-4.152648000	-2.670640000	0.632563000
C	-6.797998000	0.041339000	0.075160000
H	-7.524931000	0.835889000	-0.096924000
C	-5.435410000	0.282368000	-0.095258000
C	-1.623068000	-1.122044000	2.482060000
C	-5.630090000	2.719866000	-0.748334000
H	-6.715371000	2.697698000	-0.643853000
C	-0.492204000	-3.363910000	1.485536000
C	-6.275868000	-2.223701000	0.673248000
H	-6.562534000	-3.232392000	0.978028000
C	-4.987093000	3.894317000	-1.126785000
C	-4.871848000	1.576043000	-0.501336000
C	-2.537748000	1.025751000	3.987075000
H	-2.910570000	1.888184000	4.543039000
C	-0.600919000	-3.836336000	0.133596000
C	-7.222328000	-1.224791000	0.463714000
H	-8.287970000	-1.426842000	0.600035000
C	-1.971139000	-2.588588000	-1.887488000
C	-2.761511000	0.937673000	2.621332000
H	-3.312226000	1.712384000	2.081882000
C	-3.268858000	-0.987695000	-3.035297000

H	-3.791464000	-0.028867000	-2.978717000
C	-1.418021000	-2.889597000	-0.568287000
O	-0.090899000	-5.242905000	-1.675017000
H	-0.158262000	-4.942086000	2.924355000
H	0.245370000	-3.312708000	3.503637000
C	-2.555347000	-2.948837000	-4.196482000
H	-2.517085000	-3.568142000	-5.097075000
C	-1.841853000	-0.009401000	4.609550000
H	-1.652979000	0.017519000	5.686191000
C	-3.249708000	-1.740849000	-4.201297000
H	-3.769965000	-1.376210000	-5.089402000
C	-1.914180000	-3.376477000	-3.038013000
H	-1.361991000	-4.314281000	-2.987973000
C	-1.390258000	-1.088112000	3.858820000
H	-0.862252000	-1.909092000	4.341812000
C	0.025802000	-5.020657000	-0.477503000
C	-3.599091000	3.904841000	-1.246169000
C	0.264721000	-3.968345000	2.622631000
H	-5.570763000	4.797130000	-1.325009000
C	0.836469000	-5.965522000	0.366053000
H	-3.049738000	4.802015000	-1.539791000
H	1.322810000	-4.136849000	2.366744000
H	1.747510000	-5.470310000	0.743059000
H	0.267636000	-6.324281000	1.239453000
H	1.129411000	-6.821414000	-0.260598000
N	-0.551897000	0.454845000	-0.524603000
H	-0.513160000	0.957092000	-1.420241000
H	-0.369105000	1.154618000	0.206342000

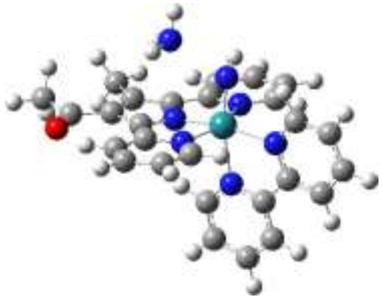

TS-ANA
E = -1596.77870544
Charge = 1 Multiplicity = 1

Ru	-0.586427000	-0.014703000	-0.550079000
N	-1.322996000	0.038596000	1.497681000
N	-2.680777000	-0.367101000	-0.713123000
N	-0.428795000	2.072638000	-0.554690000

N	0.152064000	-2.003189000	-0.351231000
N	1.250112000	0.248190000	-0.056616000
C	1.813278000	1.477772000	-0.006056000
C	-3.293773000	-0.602867000	-1.881678000
H	-2.645974000	-0.592336000	-2.762901000
C	-0.546241000	0.268706000	2.560777000
H	0.521861000	0.395037000	2.363842000
C	-3.233281000	-0.059288000	2.932323000
H	-4.308914000	-0.192052000	3.057510000
C	-2.650281000	-0.125666000	1.663924000
C	0.830069000	2.535536000	-0.248517000
C	-4.783663000	-0.617520000	0.404827000
H	-5.356841000	-0.621601000	1.332618000
C	3.190114000	1.303557000	0.224502000
C	-1.054659000	0.345653000	3.851239000
H	-0.380703000	0.534727000	4.689991000
C	-5.417562000	-0.854929000	-0.808539000
C	-3.407801000	-0.375910000	0.427216000
C	-1.237584000	4.314112000	-0.770215000
H	-2.077151000	4.978032000	-0.987737000
C	3.405452000	-0.123186000	0.313687000
C	-2.425678000	0.179421000	4.038340000
H	-2.865482000	0.235561000	5.037765000
C	1.487932000	-2.062641000	-0.034339000
C	-1.414821000	2.936053000	-0.809541000
H	-2.385882000	2.495175000	-1.051763000
C	-0.542902000	-3.121875000	-0.539763000
H	-1.599602000	-2.994964000	-0.792853000
C	2.127327000	-0.743384000	0.116879000
O	4.697988000	-2.054091000	0.684984000
H	4.655231000	2.433688000	1.350937000
H	3.738061000	3.381895000	0.166606000
C	1.373820000	-4.472825000	-0.098808000
H	1.861837000	-5.446420000	0.005383000
C	0.024751000	4.805232000	-0.445954000
H	0.209175000	5.882081000	-0.395075000
C	0.024550000	-4.388532000	-0.424293000
H	-0.589036000	-5.277736000	-0.586028000
C	2.109835000	-3.305651000	0.096874000
H	3.168948000	-3.321906000	0.352341000
C	1.059161000	3.913283000	-0.182991000
H	2.048353000	4.287256000	0.080795000
C	4.665937000	-0.835326000	0.566517000
C	-4.656697000	-0.848335000	-1.975690000

C	4.198490000	2.400696000	0.346294000
H	-6.493759000	-1.044997000	-0.839863000
C	5.958690000	-0.069494000	0.683691000
H	-5.106635000	-1.029759000	-2.954656000
H	5.019198000	2.291344000	-0.381259000
H	6.176443000	0.493535000	-0.239497000
H	5.926184000	0.654759000	1.514364000
H	6.768327000	-0.792499000	0.865917000
N	-0.363316000	-0.075710000	-2.364438000
N	1.925489000	-0.097342000	-3.025574000
H	2.571692000	0.514187000	-2.521001000
H	2.184012000	-1.063850000	-2.823577000
H	2.037391000	0.062808000	-4.028520000
H	-0.207059000	0.869549000	-2.758039000

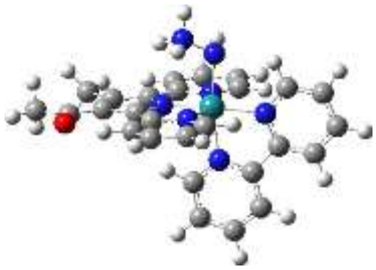

$[Ru^{II}\text{-}NH\text{-}NH_3]^+$
E = -1596.81174482
Charge = 1 Multiplicity = 1

Ru	-0.607693000	0.005316000	-0.502531000
N	-1.162251000	-0.145745000	1.488072000
N	-2.661043000	-0.302603000	-0.645407000
N	-0.483653000	2.093868000	-0.403208000
N	0.204114000	-1.942658000	-0.473406000
N	1.276573000	0.297663000	-0.204832000
C	1.810917000	1.535247000	-0.073895000
C	-3.344432000	-0.381041000	-1.797029000
H	-2.729755000	-0.297034000	-2.699229000
C	-0.312624000	-0.074897000	2.522993000
H	0.741186000	0.068498000	2.270380000
C	-2.972252000	-0.432795000	3.034332000
H	-4.038813000	-0.578009000	3.212871000
C	-2.485343000	-0.325862000	1.728487000
C	0.789396000	2.575614000	-0.184014000
C	-4.716701000	-0.584241000	0.559538000
H	-5.242913000	-0.662665000	1.512357000
C	3.198245000	1.386002000	0.155052000
C	-0.730918000	-0.174329000	3.842487000

H	0.003455000	-0.108646000	4.648956000
C	-5.422010000	-0.660007000	-0.635314000
C	-3.330166000	-0.407169000	0.529703000
C	-1.353877000	4.328030000	-0.428366000
H	-2.224274000	4.979860000	-0.532928000
C	3.455285000	-0.028343000	0.164157000
C	-2.089083000	-0.355635000	4.104130000
H	-2.457429000	-0.437439000	5.130277000
C	1.559463000	-1.992108000	-0.211744000
C	-1.503834000	2.949717000	-0.522654000
H	-2.484891000	2.499578000	-0.700380000
C	-0.479799000	-3.080152000	-0.630761000
H	-1.548085000	-2.967997000	-0.838051000
C	2.183059000	-0.674012000	-0.070598000
O	4.811082000	-1.942282000	0.348352000
H	4.614202000	2.508646000	1.354069000
H	3.690226000	3.480611000	0.195635000
C	1.473319000	-4.407151000	-0.275058000
H	1.976443000	-5.375404000	-0.194447000
C	-0.076684000	4.835613000	-0.197441000
H	0.090647000	5.913134000	-0.111585000
C	0.106571000	-4.337135000	-0.539698000
H	-0.503258000	-5.233554000	-0.674734000
C	2.200222000	-3.232706000	-0.113464000
H	3.271231000	-3.231330000	0.090336000
C	0.993502000	3.956552000	-0.075340000
H	1.995527000	4.343674000	0.109590000
C	4.728743000	-0.717053000	0.368902000
C	-4.720273000	-0.557844000	-1.837581000
C	4.173460000	2.504854000	0.341475000
H	-6.506525000	-0.798451000	-0.627466000
C	5.990638000	0.074733000	0.614176000
H	-5.231344000	-0.612970000	-2.802068000
H	5.010156000	2.451094000	-0.374409000
H	6.240441000	0.709682000	-0.252923000
H	5.896368000	0.735578000	1.491513000
H	6.812019000	-0.638371000	0.783573000
N	-0.424728000	0.118625000	-2.578024000
N	0.833737000	-0.328965000	-3.090612000
H	1.664677000	0.137762000	-2.684771000
H	0.936734000	-1.333859000	-2.892371000
H	0.895887000	-0.226769000	-4.115758000
H	-0.450263000	1.104107000	-2.856827000

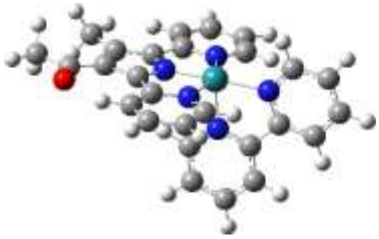

TS3-D
E = -1484.99699
Charge = 1 Multiplicity = 1

Ru	-0.595204000	0.010531000	-0.634024000
N	-1.164619000	-0.105175000	1.254521000
N	-2.635678000	-0.275761000	-0.867127000
N	-0.426405000	2.110889000	-0.539026000
N	0.180482000	-1.957504000	-0.579383000
N	1.303943000	0.275023000	-0.345065000
C	1.863483000	1.502472000	-0.246999000
C	-3.288986000	-0.343174000	-2.034296000
H	-2.675245000	-0.240774000	-2.934295000
C	-0.316991000	-0.004258000	2.293253000
H	0.733109000	0.156701000	2.043922000
C	-2.974537000	-0.414288000	2.797026000
H	-4.039112000	-0.580385000	2.969128000
C	-2.491631000	-0.305864000	1.492323000
C	0.857647000	2.563230000	-0.328571000
C	-4.710700000	-0.582030000	0.294198000
H	-5.256467000	-0.672399000	1.234895000
C	3.255975000	1.330101000	-0.092497000
C	-0.743862000	-0.100569000	3.608963000
H	-0.012043000	-0.011509000	4.415210000
C	-5.386909000	-0.651627000	-0.918387000
C	-3.326584000	-0.394805000	0.291654000
C	-1.259332000	4.356547000	-0.498190000
H	-2.119956000	5.025457000	-0.569172000
C	3.487660000	-0.091049000	-0.101338000
C	-2.097116000	-0.311464000	3.869092000
H	-2.466078000	-0.395171000	4.894562000
C	1.543449000	-2.023337000	-0.380366000
C	-1.435583000	2.983247000	-0.617210000
H	-2.427918000	2.553917000	-0.782808000
C	-0.531652000	-3.082418000	-0.676338000
H	-1.606995000	-2.957192000	-0.832938000
C	2.197080000	-0.711577000	-0.271850000
O	4.819165000	-2.028440000	-0.001774000
H	4.768673000	2.419548000	1.014517000

H	3.787921000	3.415357000	-0.074557000
C	1.413356000	-4.435976000	-0.393070000
H	1.904666000	-5.410707000	-0.318843000
C	0.031364000	4.835985000	-0.286746000
H	0.219533000	5.908628000	-0.184540000
C	0.037691000	-4.348377000	-0.587647000
H	-0.593421000	-5.236134000	-0.671589000
C	2.167706000	-3.270523000	-0.291261000
H	3.247409000	-3.285524000	-0.141631000
C	1.089033000	3.937099000	-0.201249000
H	2.101223000	4.302767000	-0.029163000
C	4.760747000	-0.803282000	0.031634000
C	-4.662863000	-0.531096000	-2.105341000
C	4.261366000	2.430108000	0.034005000
H	-6.469984000	-0.799097000	-0.936312000
C	6.045764000	-0.034366000	0.216431000
H	-5.153467000	-0.580281000	-3.080303000
H	5.046262000	2.364074000	-0.737446000
H	6.260595000	0.602271000	-0.658664000
H	6.006578000	0.621772000	1.101478000
H	6.862860000	-0.761738000	0.338994000

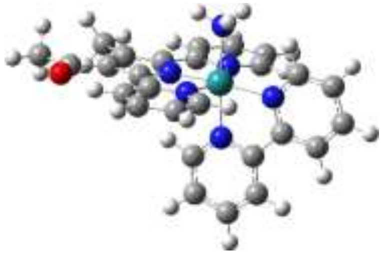

$[Ru^{II}\text{-}NH_3]^+$
E = -1541.562709
Charge = 1 Multiplicity = 1

Ru	-0.586754000	0.001111000	-0.564636000
N	-1.127703000	-0.105441000	1.404426000
N	-2.645590000	-0.291450000	-0.716258000
N	-0.426522000	2.100229000	-0.485629000
N	0.185936000	-1.952728000	-0.532377000
N	1.303735000	0.271302000	-0.276225000
C	1.859084000	1.499401000	-0.161646000
C	-3.353351000	-0.371832000	-1.853423000
H	-2.789317000	-0.282925000	-2.784590000
C	-0.270043000	-0.003828000	2.430723000
H	0.778703000	0.151648000	2.166799000
C	-2.919415000	-0.400818000	2.968830000

H	-3.981628000	-0.561993000	3.158359000
C	-2.446382000	-0.300752000	1.657894000
C	0.854006000	2.557590000	-0.264868000
C	-4.685641000	-0.580889000	0.519930000
H	-5.195574000	-0.659114000	1.481398000
C	3.249721000	1.328653000	0.024471000
C	-0.677029000	-0.091429000	3.754433000
H	0.062982000	-0.001623000	4.553238000
C	-5.410844000	-0.662771000	-0.662137000
C	-3.301234000	-0.396835000	0.468118000
C	-1.258966000	4.347499000	-0.500139000
H	-2.118260000	5.014704000	-0.598657000
C	3.485023000	-0.090207000	0.013962000
C	-2.028743000	-0.295122000	4.029874000
H	-2.386965000	-0.371691000	5.059920000
C	1.548395000	-2.022137000	-0.326444000
C	-1.432933000	2.971973000	-0.597160000
H	-2.422447000	2.539001000	-0.770609000
C	-0.520131000	-3.077975000	-0.670804000
H	-1.594641000	-2.950483000	-0.831776000
C	2.197347000	-0.713395000	-0.190030000
O	4.817501000	-2.025803000	0.134337000
H	4.719846000	2.423256000	1.183601000
H	3.775771000	3.415299000	0.059464000
C	1.426791000	-4.434727000	-0.408996000
H	1.919864000	-5.410140000	-0.358743000
C	0.027635000	4.831590000	-0.272870000
H	0.213836000	5.905868000	-0.185002000
C	0.052242000	-4.344066000	-0.616107000
H	-0.575267000	-5.230554000	-0.734310000
C	2.175446000	-3.271015000	-0.265303000
H	3.253524000	-3.285989000	-0.103672000
C	1.083017000	3.934225000	-0.154295000
H	2.092006000	4.302838000	0.030303000
C	4.753276000	-0.800458000	0.178447000
C	-4.728585000	-0.556815000	-1.873924000
C	4.247673000	2.431242000	0.185387000
H	-6.494105000	-0.807565000	-0.636898000
C	6.030524000	-0.030557000	0.412268000
H	-5.251018000	-0.614857000	-2.831820000
H	5.060390000	2.367561000	-0.556875000
H	6.274901000	0.611169000	-0.451335000
H	5.960460000	0.620713000	1.298998000
H	6.844095000	-0.757560000	0.558642000

N	-0.188979000	0.080155000	-2.651206000
H	-0.486136000	-0.766738000	-3.145897000
H	-0.646920000	0.871279000	-3.114726000
H	0.815623000	0.181713000	-2.832779000

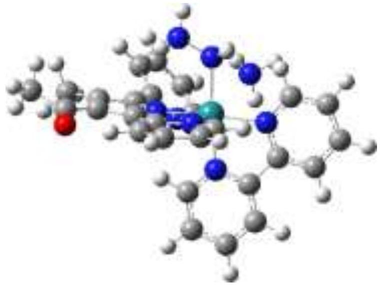

TS3-I$_a$-1
E = -1653.340644
Charge = 1 Multiplicity = 1

Ru	-0.704750000	0.117395000	-0.436854000
N	-1.397714000	-0.139867000	1.482012000
N	-2.769416000	0.114247000	-0.741544000
N	0.852191000	2.717895000	0.973528000
N	-0.387796000	-1.842553000	-0.551899000
N	1.278420000	0.000811000	0.034224000
C	2.259348000	0.926415000	0.204995000
C	-3.397720000	0.287524000	-1.912179000
H	-2.767747000	0.405681000	-2.796405000
C	-0.612008000	-0.298002000	2.556143000
H	0.462939000	-0.235322000	2.374605000
C	-3.324016000	-0.434517000	2.875617000
H	-4.408693000	-0.490835000	2.979026000
C	-2.743480000	-0.208098000	1.625895000
C	1.941079000	2.357900000	0.274048000
C	-4.907945000	-0.004107000	0.340924000
H	-5.489329000	-0.117791000	1.256844000
C	3.508060000	0.287662000	0.292982000
C	-1.125151000	-0.526233000	3.826068000
H	-0.441921000	-0.648060000	4.669871000
C	-5.552558000	0.171811000	-0.877722000
C	-3.512512000	-0.029215000	0.384888000
C	1.275826000	5.023408000	0.451628000
H	0.973115000	6.068689000	0.556806000
C	3.252279000	-1.113760000	0.173119000
C	-2.507420000	-0.595017000	3.989551000
H	-2.948458000	-0.774654000	4.973695000
C	0.879839000	-2.293296000	-0.279848000

C	0.535767000	4.007103000	1.054849000
H	-0.363055000	4.251147000	1.635429000
C	-1.357790000	-2.718625000	-0.858789000
H	-2.340670000	-2.291998000	-1.066503000
C	1.831319000	-1.233606000	0.013861000
O	3.953479000	-3.367241000	0.372810000
H	4.600053000	2.015037000	0.949839000
H	5.452053000	1.076125000	-0.293989000
C	0.134202000	-4.568742000	-0.632064000
H	0.345031000	-5.641911000	-0.655702000
C	2.398812000	4.658598000	-0.289812000
H	3.002978000	5.415613000	-0.799457000
C	-1.143452000	-4.086583000	-0.916614000
H	-1.971278000	-4.751083000	-1.174527000
C	1.141484000	-3.669929000	-0.309960000
H	2.152489000	-3.994241000	-0.062932000
C	2.734793000	3.312053000	-0.385787000
H	3.589194000	2.993343000	-0.987310000
C	4.247929000	-2.189648000	0.196417000
C	-4.780954000	0.321496000	-2.028020000
C	4.795397000	0.991769000	0.590824000
H	-6.644510000	0.193809000	-0.925939000
C	5.704828000	-1.851566000	-0.022273000
H	-5.236949000	0.464782000	-3.010420000
H	5.373135000	0.477304000	1.375651000
H	5.855210000	-1.095102000	-0.808284000
H	6.153740000	-1.463044000	0.908803000
H	6.231484000	-2.780601000	-0.291357000
N	-1.342463000	2.941804000	-1.412584000
H	-1.741671000	3.038673000	-0.475497000
H	-2.097627000	3.151718000	-2.070847000
H	-0.644609000	3.684601000	-1.503955000
N	-0.188671000	0.409022000	-2.471257000
H	-0.757429000	-0.204695000	-3.059287000
H	-0.521328000	1.378429000	-2.590903000
N	1.162291000	0.236968000	-2.881451000
H	1.310681000	0.758681000	-3.749642000
H	1.750675000	0.671185000	-2.168179000

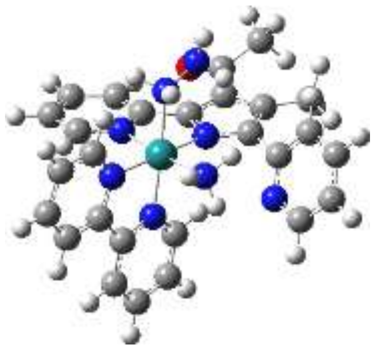

TS3-I$_a$-m
E = -1653.37224
Charge = 1 Multiplicity = 1

Ru	-0.748542000	0.073382000	-0.521961000
N	-1.481634000	0.341878000	1.358520000
N	-2.781846000	0.029072000	-0.887789000
N	1.302834000	2.854522000	0.939285000
N	-0.498842000	-1.935807000	-0.188807000
N	1.247442000	-0.041879000	0.036565000
C	2.283552000	0.839894000	0.049989000
C	-3.372508000	-0.135221000	-2.080650000
H	-2.713588000	-0.275181000	-2.940588000
C	-0.728903000	0.461220000	2.462550000
H	0.350070000	0.418744000	2.306505000
C	-3.450150000	0.568622000	2.710706000
H	-4.537973000	0.609781000	2.784469000
C	-2.833412000	0.383357000	1.471103000
C	2.083997000	2.299189000	-0.000465000
C	-4.957170000	0.217715000	0.111728000
H	-5.569200000	0.358517000	1.003828000
C	3.503555000	0.163650000	0.173876000
C	-1.280281000	0.638992000	3.724014000
H	-0.621582000	0.731210000	4.590872000
C	-5.561264000	0.039963000	-1.127180000
C	-3.563912000	0.212185000	0.208215000
C	1.695065000	5.020244000	-0.024128000
H	1.508537000	6.097046000	0.009954000
C	3.180156000	-1.225687000	0.279851000
C	-2.667295000	0.698879000	3.851746000
H	-3.136543000	0.843030000	4.828630000
C	0.759843000	-2.360364000	0.131056000
C	1.117456000	4.172033000	0.921023000
H	0.469466000	4.582740000	1.705901000
C	-1.500334000	-2.822892000	-0.268995000
H	-2.481425000	-2.416090000	-0.525028000
C	1.750047000	-1.293541000	0.193811000

O	3.835959000	-3.438854000	0.838382000
H	4.688246000	1.935527000	0.415899000
H	5.461791000	0.708245000	-0.606636000
C	-0.051636000	-4.634552000	0.285153000
H	0.132053000	-5.695816000	0.478183000
C	2.506477000	4.451502000	-1.003518000
H	2.975979000	5.072946000	-1.772380000
C	-1.328276000	-4.181070000	-0.046706000
H	-2.182154000	-4.857836000	-0.127869000
C	0.990125000	-3.721848000	0.379209000
H	2.000513000	-4.023377000	0.655220000
C	2.704157000	3.073021000	-0.993595000
H	3.321066000	2.587827000	-1.754600000
C	4.141717000	-2.321800000	0.433607000
C	-4.750995000	-0.137524000	-2.246777000
C	4.829470000	0.849918000	0.287883000
H	-6.650858000	0.041241000	-1.215473000
C	5.589321000	-2.081425000	0.067575000
H	-5.173361000	-0.278767000	-3.244452000
H	5.410237000	0.491953000	1.154439000
H	5.704105000	-1.493993000	-0.856848000
H	6.107352000	-1.538583000	0.877422000
H	6.075596000	-3.062495000	-0.050324000
N	-0.899523000	2.159958000	-0.949977000
H	-1.002596000	2.717083000	-0.097489000
H	-1.711547000	2.370682000	-1.539693000
H	-0.078883000	2.535095000	-1.435493000
N	-0.171435000	-0.187191000	-2.570643000
H	-0.582743000	-1.071052000	-2.882930000
H	-0.630852000	0.549054000	-3.120312000
N	1.217901000	-0.244956000	-2.869852000
H	1.359136000	-0.001879000	-3.853594000
H	1.680254000	0.470440000	-2.305970000

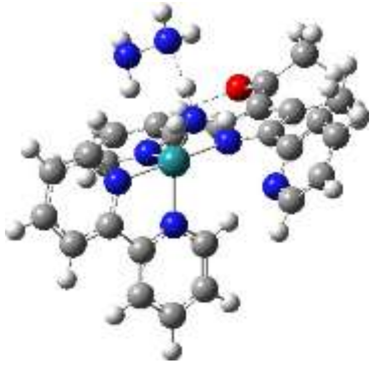

TS3-I$_a$-2
E = -1653.321584
Charge = 1 Multiplicity = 1

Ru	0.718810000	0.151563000	0.375116000
N	1.514835000	0.303922000	-1.425694000
N	2.727076000	0.119085000	0.845939000
N	-0.888257000	2.874899000	-0.669647000
N	0.487885000	-1.789376000	-0.068463000
N	-1.269136000	0.073455000	-0.119726000
C	-2.276220000	0.987508000	-0.144288000
C	3.239883000	-0.047157000	2.072441000
H	2.519525000	-0.204779000	2.880353000
C	0.787306000	0.347884000	-2.556893000
H	-0.291964000	0.248116000	-2.432992000
C	3.512139000	0.577601000	-2.725946000
H	4.599151000	0.666742000	-2.763484000
C	2.872479000	0.396215000	-1.498808000
C	-1.991649000	2.423962000	-0.047889000
C	4.945907000	0.305915000	-0.042171000
H	5.604768000	0.436948000	-0.902299000
C	-3.508940000	0.334692000	-0.304232000
C	1.372244000	0.517841000	-3.801567000
H	0.736477000	0.549395000	-4.689520000
C	5.475736000	0.147438000	1.233141000
C	3.559995000	0.284542000	-0.210995000
C	-1.346331000	5.096184000	0.119467000
H	-1.048196000	6.147445000	0.155017000
C	-3.218958000	-1.063653000	-0.385314000
C	2.759270000	0.643286000	-3.891461000
H	3.249082000	0.785016000	-4.858267000
C	-0.794275000	-2.225656000	-0.294780000
C	-0.579365000	4.165037000	-0.580083000
H	0.333399000	4.482443000	-1.100280000
C	1.509290000	-2.663011000	-0.098329000
H	2.503648000	-2.247901000	0.076157000

C	-1.791037000	-1.167006000	-0.283815000
O	-3.893162000	-3.296906000	-0.813472000
H	-4.655007000	2.093492000	-0.751729000
H	-5.442023000	1.024904000	0.427969000
C	0.034278000	-4.487585000	-0.537659000
H	-0.151895000	-5.549931000	-0.720314000
C	-2.490640000	4.638526000	0.771022000
H	-3.117939000	5.324569000	1.348587000
C	1.330843000	-4.017444000	-0.323072000
H	2.198559000	-4.681022000	-0.331535000
C	-1.022768000	-3.589007000	-0.529939000
H	-2.051077000	-3.899210000	-0.715951000
C	-2.815388000	3.287828000	0.695298000
H	-3.681932000	2.895809000	1.232109000
C	-4.197644000	-2.150405000	-0.502083000
C	4.607889000	-0.038091000	2.309559000
C	-4.818974000	1.037058000	-0.484896000
H	6.558419000	0.161178000	1.384245000
C	-5.652096000	-1.869326000	-0.200384000
H	4.981023000	-0.177707000	3.326787000
H	-5.417545000	0.588033000	-1.293018000
H	-5.791496000	-1.199871000	0.662544000
H	-6.141538000	-1.401340000	-1.072670000
H	-6.149035000	-2.833445000	-0.009692000
N	0.298437000	1.495904000	2.059939000
H	-0.371873000	2.244058000	1.866249000
H	1.112874000	1.914681000	2.517694000
H	-0.159337000	0.829105000	2.744874000
N	-0.831933000	-0.629178000	3.607561000
H	-1.762154000	-0.847873000	3.235309000
H	-0.923696000	-0.526647000	4.618842000
N	0.117301000	-1.635820000	3.329481000
H	-0.310755000	-2.566160000	3.331834000
H	0.476900000	-1.478872000	2.382204000

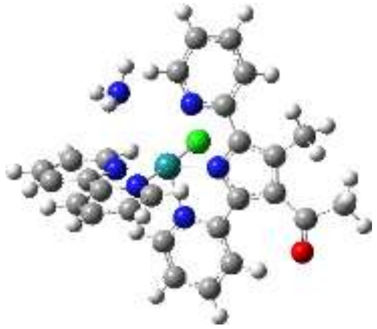

TS1 Ru-Cl→Ru-NH_3
E = -2001.760993
Charge = 0   Multiplicity = 1

Ru	-0.663343000	0.010377000	-0.491497000
N	-1.359167000	-0.001515000	1.418370000
N	-2.709844000	-0.114340000	-0.810691000
N	0.923914000	2.823015000	0.827613000
N	-0.272742000	-1.929026000	-0.347604000
N	1.336117000	0.028775000	-0.037607000
C	2.281958000	1.000830000	0.032224000
C	-3.306359000	-0.161201000	-2.008297000
H	-2.624837000	-0.185916000	-2.864309000
C	-0.578489000	-0.008270000	2.509973000
H	0.497191000	0.020048000	2.324952000
C	-3.292632000	-0.078288000	2.835289000
H	-4.378223000	-0.115258000	2.938388000
C	-2.708876000	-0.045855000	1.567367000
C	1.925126000	2.427443000	0.025755000
C	-4.864329000	-0.068558000	0.238836000
H	-5.464209000	-0.025484000	1.149432000
C	3.558466000	0.425513000	0.136908000
C	-1.097809000	-0.042973000	3.796240000
H	-0.417603000	-0.044883000	4.651363000
C	-5.482226000	-0.115091000	-1.006008000
C	-3.469451000	-0.073697000	0.310790000
C	1.226628000	5.088557000	0.089816000
H	0.913580000	6.134256000	0.154135000
C	3.362260000	-0.991697000	0.140733000
C	-2.481880000	-0.074446000	3.964449000
H	-2.926261000	-0.101195000	4.962936000
C	1.024781000	-2.298748000	-0.097728000
C	0.589018000	4.109805000	0.850984000
H	-0.237437000	4.384657000	1.518957000
C	-1.222681000	-2.869773000	-0.472875000
H	-2.232639000	-2.507047000	-0.670272000
C	1.941806000	-1.179444000	0.031104000

O	4.171964000	-3.195251000	0.474566000
H	4.583912000	2.259330000	0.574372000
H	5.458368000	1.223383000	-0.571365000
C	0.354514000	-4.625529000	-0.111125000
H	0.607530000	-5.685400000	-0.013078000
C	2.262937000	4.686531000	-0.751894000
H	2.785572000	5.412495000	-1.382654000
C	-0.957793000	-4.225568000	-0.366582000
H	-1.772870000	-4.944204000	-0.479793000
C	1.340953000	-3.659785000	0.026222000
H	2.378756000	-3.913683000	0.243504000
C	2.615096000	3.341341000	-0.789383000
H	3.402590000	2.989144000	-1.460051000
C	4.404629000	-2.015924000	0.223871000
C	-4.688130000	-0.164985000	-2.151181000
C	4.818881000	1.210828000	0.329808000
H	-6.573166000	-0.111611000	-1.079192000
C	5.844850000	-1.625676000	-0.023769000
H	-5.128940000	-0.202728000	-3.150387000
H	5.431603000	0.814256000	1.155896000
H	5.960607000	-0.939132000	-0.877025000
H	6.270583000	-1.129728000	0.866116000
H	6.417642000	-2.548193000	-0.207262000
Cl	-0.060134000	0.060513000	-2.839765000
N	-2.124587000	2.905286000	-0.540769000
H	-2.244831000	2.853582000	0.473172000
H	-3.054694000	2.817013000	-0.956555000
H	-1.781873000	3.845555000	-0.750301000

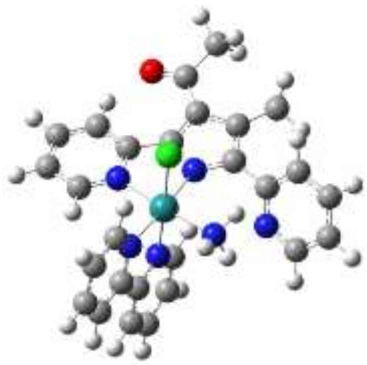

M1 Ru-Cl→Ru-NH_3
E = -2001.794901
Charge = 0 Multiplicity = 1

Ru	-0.722142000	0.038754000	-0.501568000
N	-1.454481000	0.370586000	1.349972000
N	-2.743790000	-0.034568000	-0.867205000

N	1.291992000	2.857172000	0.889011000
N	-0.443677000	-1.945584000	-0.081424000
N	1.287576000	-0.033840000	0.005092000
C	2.306468000	0.865987000	-0.015790000
C	-3.309939000	-0.262754000	-2.061342000
H	-2.603448000	-0.443718000	-2.877719000
C	-0.703525000	0.511383000	2.455618000
H	0.375136000	0.451611000	2.302808000
C	-3.424579000	0.661218000	2.694516000
H	-4.512711000	0.713882000	2.761468000
C	-2.809138000	0.430274000	1.461691000
C	2.078789000	2.321188000	-0.057673000
C	-4.928401000	0.229122000	0.089597000
H	-5.551599000	0.428066000	0.963152000
C	3.542476000	0.213869000	0.079340000
C	-1.255761000	0.733239000	3.708370000
H	-0.597256000	0.841797000	4.573695000
C	-5.512441000	-0.012989000	-1.148338000
C	-3.536245000	0.213589000	0.206677000
C	1.642696000	5.037595000	-0.057426000
H	1.437328000	6.110688000	-0.014310000
C	3.249433000	-1.182343000	0.198771000
C	-2.643496000	0.816926000	3.832951000
H	-3.111745000	0.996544000	4.804422000
C	0.835582000	-2.352592000	0.170743000
C	1.083052000	4.171164000	0.881754000
H	0.430154000	4.563590000	1.672176000
C	-1.443053000	-2.837217000	-0.058660000
H	-2.440583000	-2.442880000	-0.266884000
C	1.815990000	-1.275019000	0.153750000
O	3.957852000	-3.395728000	0.694763000
H	4.698207000	2.008431000	0.295938000
H	5.465960000	0.804377000	-0.757003000
C	0.050218000	-4.623797000	0.458563000
H	0.252867000	-5.677378000	0.674055000
C	2.461631000	4.490289000	-1.043014000
H	2.917854000	5.125561000	-1.808664000
C	-1.249600000	-4.185575000	0.202842000
H	-2.103684000	-4.867156000	0.206184000
C	1.090830000	-3.705096000	0.446628000
H	2.120865000	-3.992420000	0.658328000
C	2.682306000	3.115638000	-1.045449000
H	3.302893000	2.648278000	-1.814291000
C	4.233208000	-2.259715000	0.317987000

C	-4.686468000	-0.263148000	-2.244518000
C	4.857470000	0.926612000	0.157037000
H	-6.600562000	-0.005326000	-1.255202000
C	5.674033000	-1.982174000	-0.053102000
H	-5.099707000	-0.456051000	-3.237731000
H	5.472301000	0.577192000	1.003302000
H	5.770968000	-1.384358000	-0.972870000
H	6.185661000	-1.435529000	0.758189000
H	6.180617000	-2.951261000	-0.183896000
Cl	-0.060885000	-0.323472000	-2.818510000
N	-0.892976000	2.097627000	-1.020260000
H	-0.962451000	2.717680000	-0.209195000
H	-1.719246000	2.266029000	-1.602339000
H	-0.083090000	2.408242000	-1.563493000

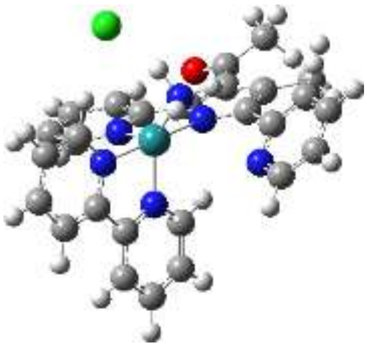

TS2 Ru-Cl→Ru-NH_3
E = -2001.752822
Charge = 0 Multiplicity = 1

Ru	-0.688616000	0.140866000	-0.321320000
N	-1.325289000	0.201462000	1.543946000
N	-2.729958000	0.154201000	-0.620635000
N	0.944482000	2.818585000	0.680612000
N	-0.435779000	-1.814832000	-0.016683000
N	1.323609000	0.040100000	0.031829000
C	2.328359000	0.956339000	0.061060000
C	-3.340398000	0.072688000	-1.812379000
H	-2.679013000	-0.072738000	-2.680610000
C	-0.507379000	0.161998000	2.613317000
H	0.557360000	0.069124000	2.394815000
C	-3.204940000	0.386082000	3.025538000
H	-4.284216000	0.471078000	3.161426000
C	-2.672353000	0.295937000	1.738428000
C	2.036195000	2.393823000	0.020959000
C	-4.860751000	0.363443000	0.456118000
H	-5.445578000	0.473637000	1.370982000

C	3.566404000	0.301657000	0.167106000
C	-0.984925000	0.240931000	3.911236000
H	-0.276829000	0.205320000	4.742635000
C	-5.494837000	0.297119000	-0.779310000
C	-3.467275000	0.282914000	0.509544000
C	1.382141000	5.069391000	-0.032511000
H	1.081989000	6.120647000	-0.020950000
C	3.281330000	-1.099578000	0.207306000
C	-2.358812000	0.361457000	4.126560000
H	-2.765716000	0.429022000	5.138744000
C	0.854248000	-2.262569000	0.131394000
C	0.630791000	4.109931000	0.645237000
H	-0.272432000	4.404726000	1.194830000
C	-1.455753000	-2.689876000	-0.008900000
H	-2.454464000	-2.264222000	-0.122493000
C	1.851126000	-1.203297000	0.136140000
O	3.962729000	-3.344973000	0.547376000
H	4.719634000	2.048204000	0.644044000
H	5.479120000	1.021750000	-0.590064000
C	0.032626000	-4.536557000	0.260770000
H	0.224630000	-5.608222000	0.367531000
C	2.513933000	4.639437000	-0.723765000
H	3.128673000	5.348573000	-1.286814000
C	-1.270250000	-4.055854000	0.120793000
H	-2.137356000	-4.720351000	0.114021000
C	1.089517000	-3.637711000	0.272169000
H	2.123235000	-3.958154000	0.401689000
C	2.842041000	3.287594000	-0.706625000
H	3.697880000	2.918598000	-1.275859000
C	4.262193000	-2.187661000	0.271937000
C	-4.722914000	0.141107000	-1.931193000
C	4.879069000	1.001707000	0.338021000
H	-6.584752000	0.360096000	-0.840501000
C	5.713272000	-1.895227000	-0.036542000
H	-5.183428000	0.071677000	-2.919761000
H	5.499128000	0.528334000	1.115494000
H	5.842439000	-1.199116000	-0.879766000
H	6.212316000	-1.454398000	0.844465000
H	6.208618000	-2.852320000	-0.263400000
Cl	-0.707443000	-0.738058000	-4.072525000
N	-0.413547000	1.566414000	-1.989100000
H	0.474733000	2.073832000	-2.010914000
H	-1.168616000	2.249228000	-2.098231000
H	-0.454110000	0.911500000	-2.813532000

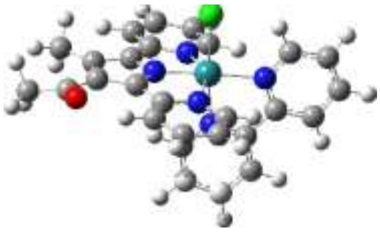

[Ru^{IV}-Cl]$^{2+}$
E = -1944.512804
Charge = 2   Multiplicity = 3

Ru	-0.594191000	-0.002910000	-0.541756000
N	-1.141361000	-0.089120000	1.440903000
N	-2.651497000	-0.307779000	-0.691149000
N	-0.416954000	2.064074000	-0.291551000
N	0.216630000	-1.927039000	-0.302854000
N	1.320766000	0.284817000	-0.305940000
C	1.858440000	1.483563000	-0.172266000
C	-3.331309000	-0.401894000	-1.841212000
H	-2.740227000	-0.314098000	-2.755027000
C	-0.279863000	0.033123000	2.457901000
H	0.768968000	0.191489000	2.202761000
C	-2.933190000	-0.383409000	2.990276000
H	-3.993105000	-0.552736000	3.177508000
C	-2.457828000	-0.292408000	1.682794000
C	0.869404000	2.538580000	-0.175647000
C	-4.685859000	-0.596977000	0.532851000
H	-5.205582000	-0.669731000	1.487768000
C	3.301285000	1.313102000	0.011034000
C	-0.693002000	-0.044168000	3.779921000
H	0.042258000	0.059712000	4.579028000
C	-5.392300000	-0.693697000	-0.660740000
C	-3.306188000	-0.405252000	0.491190000
C	-1.254853000	4.290285000	-0.187240000
H	-2.124948000	4.948287000	-0.194724000
C	3.528747000	-0.072210000	0.009578000
C	-2.042130000	-0.257560000	4.048898000
H	-2.401250000	-0.326827000	5.077650000
C	1.591155000	-2.004545000	-0.218259000
C	-1.441742000	2.912078000	-0.294677000
H	-2.441110000	2.480441000	-0.383557000
C	-0.519073000	-3.031811000	-0.307440000
H	-1.602034000	-2.901808000	-0.362087000
C	2.237161000	-0.703761000	-0.195898000
O	4.860129000	-1.992650000	-0.107775000
H	4.049336000	2.989487000	1.080513000
H	4.016694000	3.184124000	-0.676705000

C	1.437660000	-4.408156000	-0.160342000
H	1.919600000	-5.386263000	-0.104855000
C	0.036147000	4.789362000	-0.062069000
H	0.211135000	5.862939000	0.031616000
C	0.056678000	-4.303565000	-0.240365000
H	-0.589216000	-5.182716000	-0.246494000
C	2.214763000	-3.250786000	-0.150404000
H	3.302163000	-3.279326000	-0.101697000
C	1.112266000	3.905742000	-0.052877000
H	2.134449000	4.268294000	0.047602000
C	4.806165000	-0.808168000	0.173265000
C	-4.705133000	-0.595919000	-1.867613000
C	4.225475000	2.460977000	0.127452000
H	-6.473552000	-0.845142000	-0.645199000
C	6.021835000	-0.105543000	0.697027000
H	-5.220008000	-0.666859000	-2.826743000
H	5.278925000	2.180262000	0.074811000
H	6.475588000	0.500092000	-0.104095000
H	5.790538000	0.561344000	1.538955000
H	6.750516000	-0.866206000	1.004716000
Cl	-0.206301000	0.085760000	-2.861118000

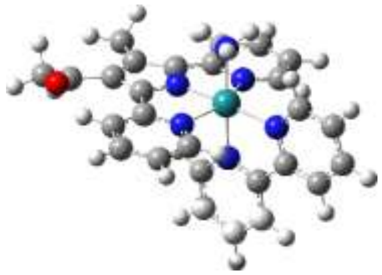

$[Ru^{IV}\text{-}NH_3]^{3+}$
E = -1540.756629
Charge = 3 Multiplicity = 3

Ru	-0.628848000	0.030740000	-0.648406000
N	-1.110325000	-0.019669000	1.364442000
N	-2.690989000	-0.290967000	-0.708932000
N	-0.351941000	2.108494000	-0.433737000
N	0.144937000	-1.902537000	-0.423940000
N	1.315009000	0.271888000	-0.455083000
C	1.894452000	1.455070000	-0.298971000
C	-3.409531000	-0.409137000	-1.834166000
H	-2.867733000	-0.337146000	-2.777854000
C	-0.217732000	0.119470000	2.351065000
H	0.822033000	0.279348000	2.063607000
C	-2.846754000	-0.306331000	2.979703000

H	-3.898228000	-0.482860000	3.203732000
C	-2.414532000	-0.229863000	1.657043000
C	0.946969000	2.543075000	-0.317297000
C	-4.677820000	-0.574004000	0.588317000
H	-5.164898000	-0.632912000	1.561221000
C	3.320625000	1.236309000	-0.057622000
C	-0.585126000	0.058536000	3.688168000
H	0.176917000	0.177481000	4.459514000
C	-5.423376000	-0.697072000	-0.579073000
C	-3.302813000	-0.370986000	0.497716000
C	-1.116575000	4.361544000	-0.368092000
H	-1.964946000	5.047114000	-0.389730000
C	3.499013000	-0.151378000	-0.055227000
C	-1.921938000	-0.159073000	4.006639000
H	-2.245673000	-0.216710000	5.047726000
C	1.515395000	-2.021924000	-0.329044000
C	-1.349388000	2.987705000	-0.454900000
H	-2.362017000	2.587752000	-0.540106000
C	-0.623714000	-2.986483000	-0.423918000
H	-1.702075000	-2.827349000	-0.484504000
C	2.194502000	-0.741002000	-0.309742000
O	4.791051000	-2.090340000	-0.240101000
H	4.047695000	2.907434000	1.033366000
H	4.178884000	3.057929000	-0.724340000
C	1.292558000	-4.418307000	-0.254125000
H	1.744214000	-5.410081000	-0.188616000
C	0.189001000	4.820791000	-0.243762000
H	0.397754000	5.889520000	-0.165880000
C	-0.084536000	-4.272544000	-0.343869000
H	-0.756453000	-5.131996000	-0.347514000
C	2.102606000	-3.284707000	-0.247395000
H	3.188329000	-3.346750000	-0.193822000
C	1.236206000	3.903035000	-0.214245000
H	2.268867000	4.234397000	-0.113481000
C	4.745592000	-0.935093000	0.142336000
C	-4.781050000	-0.613922000	-1.811617000
C	4.280731000	2.347437000	0.111628000
H	-6.502034000	-0.857382000	-0.523330000
C	5.925655000	-0.313522000	0.823775000
H	-5.328110000	-0.704929000	-2.750804000
H	5.320100000	2.016481000	0.158451000
H	6.492519000	0.294414000	0.099517000
H	5.633580000	0.334771000	1.661172000
H	6.582460000	-1.119612000	1.175136000

N	-0.239959000	0.034907000	-2.728876000
H	-0.569029000	-0.821367000	-3.184104000
H	-0.706019000	0.815168000	-3.201293000
H	0.757152000	0.116899000	-2.944449000

Supplementary References

[1] A. McSkimming, V. Diachenko. R. London, K. Olrich, C. J. Onie, M. M. Bhadhade, M. P. Bucknall, R. W. Read, S. B. Colbran, *Chem. Eur. J.* **2014**, *20*, 11445.

[2] I. P. Evans, A. Spencer, G. Wilkinson, *J. Chem. Soc., Dalton Trans.* **1973**, *2*, 204.

[3] G. M. Sheldrick, SADABS, University of Göttingen, Germany **1997**.

[4] G. M. Sheldrick, SHELXTL Version 5.1. Software.

[5] G. Chen, Z. W. Chen, Y. M. Wang, P. He, C. Liu, H. X. Tong, X. Y. Yi, *Inorg. Chem.* **2021**, *60*, 15627.

[6] G. Chen, T. Fan, B. Liu, M. Xue, J. J. Wei, S. R. Kang, H. X. Tong, X. Y. Yi, *Dalton Trans.* **2021**, *50*, 2018.

[7] J. Yang, L. Wang, S. Zhan, H. Zou, H. Chen, M. S. G. Ahlquist, L. Duan, L. Sun, *Nat. Common.* **2021**, *12*, 373.

[8] G. Chen, P. He, C. Liu, X.-F. Mo, J,-J Wei, Z.-W. Chen, T. Cheng, L.-Z. Fu, X.-Y. Yi, *Nat. Catal.*, **2023**, *6*, 949.

[9] G. W. Watt, J. D. Chrisp, *Anal. Chem.* **1952**, *24*, 2006.

[10] H. Y. Liu, H. M. C. Lant, J. L. Troiano, G. Hu, B. Q. Mercado, R. H. Crabtree, G. W. Brudvig, *J. Am. Chem. Soc.* **2022**, *144*, 8449.

[11] Q. Chen, J. Liang, L. Yue, Y. Luo, Q. Liu, N. Li, A. A. Alshehri, T. Li, H. Guo, X. Sun, *Chem. Commun.* **2022**, *58*, 5091.

[12] D. Zhu, L. Zhang, R. E. Ruther, R. J. Hamers, *Nat. Mater.* **2013**, *12*, 836.

[13] C. Adamo, V. Barone, *J. Chem. Phys.* **1999**, *110*, 6158.

[14] S. Grimme, S. Ehrlich, L. Goerigk, *J. Comput. Chem.* **2011**, *32*, 1456.

[15] F. Weigend, R. Ahlrichs, *Phys. Chem. Chem. Phys.* **2005**, *7*, 3297.

[16] F. Weigend, *Phys. Chem. Chem. Phys.* **2006**, *8*, 1057.

[17] A. V. Marenich, C. J. Cramer, D. G. Truhlar, *J. Phys. Chem. B* **2009**, *113*, 6378.
